# Modulation of chronic obstructive pulmonary disease progression by antioxidant metabolites from *Pediococcus pentosaceus*: enhancing gut probiotics abundance and the tryptophan-melatonin pathway

**DOI:** 10.1080/19490976.2024.2320283

**Published:** 2024-03-06

**Authors:** Yiting Liu, Longjie Li, Jing Feng, Bing Wan, Qiang Tu, Wei Cai, Fa Jin, Guiying Tang, Lígia R. Rodrigues, Xiuwei Zhang, Jia Yin, Yunlei Zhang

**Affiliations:** a Department of Respiratory and Critical Care Medicine, Central Laboratory, Translational Medicine Research Center, Department of Pathology, The Affiliated Jiangning Hospital of Nanjing Medical University, Nanjing, P. R. China; b The Key Laboratory of Clinical and Medical Engineering, School of Biomedical Engineering and Informatics, Nanjing Medical University, Nanjing, P. R. China; c CAS Key Laboratory of Quantitative Engineering Biology, Shenzhen Institute of Synthetic Biology, Shenzhen Institutes of Advanced Technology, Chinese Academy of Sciences, Shenzhen, China; d CEB - Centre of Biological Engineering, University of Minho, Braga, Portugal; e Hunan Provincial Key Laboratory of Animal Intestinal Function and Regulation, Hunan International Joint Laboratory of Animal Intestinal Ecology and Health, College of Life Sciences, Hunan Normal University, Changsha, China

**Keywords:** Microbial metabolites, oxidative stress, COPD, gut-lung axis

## Abstract

Chronic obstructive pulmonary disease (COPD), a condition primarily linked to oxidative stress, poses significant health burdens worldwide. Recent evidence has shed light on the association between the dysbiosis of gut microbiota and COPD, and their metabolites have emerged as potential modulators of disease progression through the intricate gut-lung axis. Here, we demonstrate the efficacy of oral administration of the probiotic *Pediococcus pentosaceus* SMM914 (SMM914) in delaying the progression of COPD by attenuating pulmonary oxidative stress. Specially, SMM914 induces a notable shift in the gut microbiota toward a community structure characterized by an augmented abundance of probiotics producing short-chain fatty acids and antioxidant metabolisms. Concurrently, SMM914 synthesizes L-tryptophanamide, 5-hydroxy-L-tryptophan, and 3-sulfino-L-alanine, thereby enhancing the tryptophan-melatonin pathway and elevating 6-hydroxymelatonin and hypotaurine in the lung environment. This modulation amplifies the secretion of endogenous anti-inflammatory factors, diminishes macrophage polarization toward the M1 phenotype, and ultimately mitigates the oxidative stress in mice with COPD. The demonstrated efficacy of the probiotic intervention, specifically with SMM914, not only highlights the modulation of intestine microbiota but also emphasizes the consequential impact on the intricate interplay between the gastrointestinal system and respiratory health.

## GRAPHICAL ABSTRACT

**Figure:**
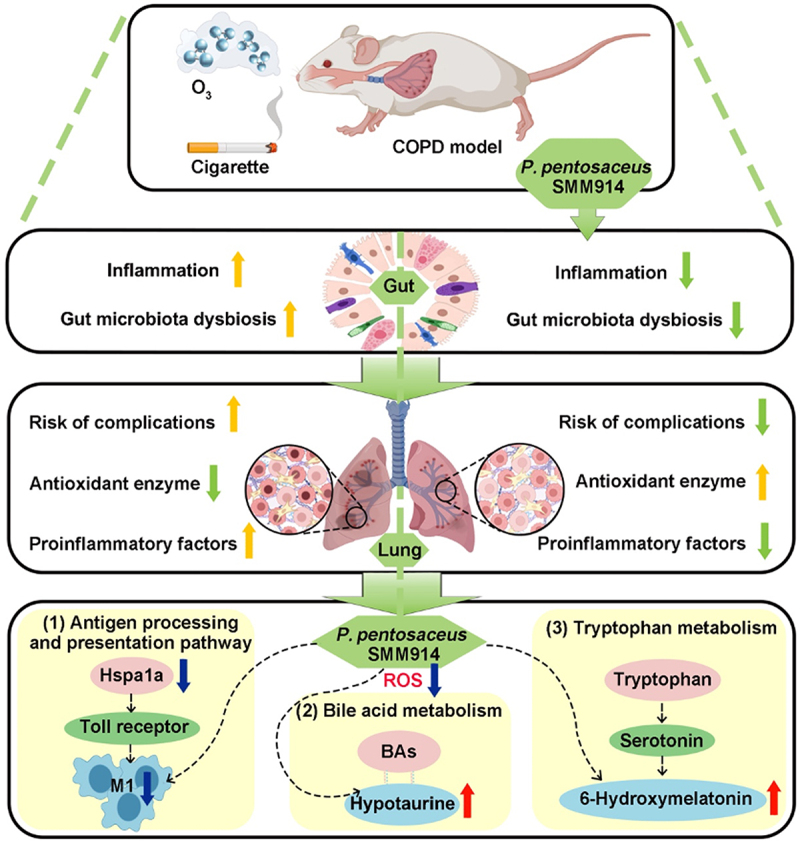

## Introduction

Chronic obstructive pulmonary disease (COPD) is a common respiratory disease that kills millions of people worldwide every year. The current standard of care for COPD includes long-acting β2 agonists, long-acting muscarinic antagonists, and inhaled corticosteroids,^1,2^ which transiently improve airflow limitation and inflammation but do not halt disease progression.^3^ Also, the COVID-19 pandemic presents additional challenges for COPD management, as nebulizer use may increase infection risk.^4^ Cigarette smoking and exposure to airborne pollutants, such as fine particulate matter (PM2.5) and tar, are the primary pathogenic factors in COPD.^5^ These pollutants induce respiratory cell stress and disrupt the balance of reactive oxygen species (ROS) levels in the lungs, contributing to the progression of COPD.^6,7^

Of note, clinical studies have shown that classic antioxidants such as glutathione and the activator of the antioxidant factor Nrf2 have the potential to halt the progression of COPD.^8,9^ Correspondingly, approaches aimed at increasing antioxidant production, and addressing low levels of reactive oxygen species to restore oxidative homeostasis in the lungs would be an optimal approach for COPD management. *Lactobacillus* and *Bifidobacterium*, as natural antioxidants, have been shown to exert protective effects against oxidative stress-related diseases.^10,11^ As the essential components of intestinal microbiota, probiotics possess the ability to strengthen the mucosal immune barrier, regulate the microbial structure of the gut, and produce antioxidant metabolites.^12^ Growing evidence has established the close relationship between gut microbiota and inflammation, neurological disorders, and oxidative stress in the body.^13,14^ The gut-brain axis,^15,16^ gut-liver axis,^17,18^ and gut-lung axis^19,20^ have demonstrated the intricate crosstalk between various organs and the gut microbiota. For instance, in 2017, Hansbro et al. elaborated on the regulatory effects of microbiota on the proximal and distal immune response in the mucosal immune system depending on the similarity of the respiratory tract and intestinal tract in anatomical structure, which preliminarily confirmed the crosstalk between the lung and intestinal microbiota.^20^ Furthermore, intestinal microbial metabolites, such as short-chain fatty acids (SCFAs), could be transported to the lungs *via* the bloodstream, which decreases the expression of pro-inflammatory factors, increases the number of immune cells that migrate to the lungs, and modulates the level of oxidative stress in the lung.^19,21–23^ Although the link between gut microbiota and lung disease remains unclear, the gut-lung interaction has been well established, where the intestinal microbes regulate the levels of oxidative stress and homeostasis in the lung through various pathways.^24,25^

In this study, our objective was to investigate the potential therapeutic effects of orally administered probiotics on COPD and elucidate the molecular mechanisms by which live bacterial therapeutics alleviate oxidative stress in the lung. We specifically utilized *Pediococcus pentosaceus* SMM914 (referred to as SMM914), a probiotic bacterium we previously isolated from sow milk, due to its desirable properties, including antioxidant capabilities.^26^ Through mass spectrometry analysis, we discerned a robust capacity of SMM914 to generate precursors of antioxidant metabolites, including L-tryptophanamide, 5-hydroxy-L-tryptophan, and 3-sulfino-L-alanin. Our animal study demonstrated that oral administration of SMM914 significantly boosts beneficial microbiota and increases endogenous antioxidant metabolites via the tryptophan-melatonin metabolism pathway in the lungs. This leads to a notable reduction in pulmonary oxidative stress, consequently alleviating COPD symptoms in mice. This study highlights the ability of living bacterial therapeutics, exemplified by SMM914, to modulate the gut-lung axis and promote oxidative homeostasis in the lung, particularly in the context of COPD management.

## Results

### Oral SMM914 mitigates the pathogenesis of COPD induced by cigarette smoking

**Figure 1.**
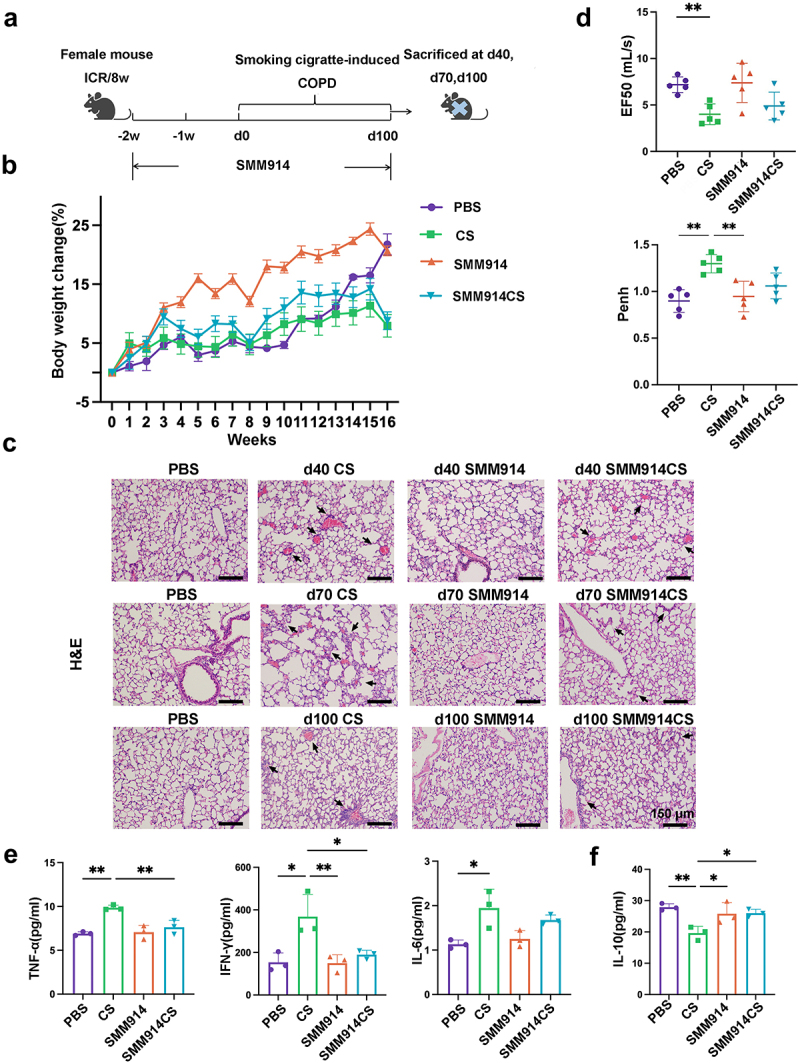
Oral SMM914 attenuates CS-induced COPD. (a) Schematic diagram of the experimental system: mice received the oral gavage of normal saline (PBS and CS group) or SMM914 or SMM914 CS for 14 weeks (1×10^9^ CFU/twice a week) before and after being subjected to cigarette smoking. Time-related comparison from CS exposure (day 40,70, and 100) following smoking exposure. (b) Body weight change percentage. (c) The representative images of lung histopathology. The black arrows indicate damaged areas, characterized with denatured and collapsed epithelial cells, thickened alveolar septa, alveolar damage, and activated inflammatory cell infiltration. *N* = 3. (d) Histological scoring of lung tissues was evaluated in a blinded manner according to INHAND. (e) Lung functions analyses including Penh and EF_50_ were measured after CS exposure for 14 weeks. (f) Serum TNF-α, IFN-γ and IL-6 concentrations after 14 weeks of CS treatment in the four groups described in (a). (g) Serum IL-10 concentrations after 14 weeks of CS exposure in the four groups described in (a).

To explore the potential of SMM914 in attenuating COPD development, SMM914 was orally administrated to female ICR mice (1×10^9^ CFU/twice a week) prior to cigarette smoke induction (Figure 1(a)). Mice were exposed to cigarette smoke for a total of 14 weeks, and compared with the healthy control group (PBS) mice, the former exhibited a significant decrease in body weight. Comparatively, cigarette smoking (CS) induced mice in the SMM914-treated group had an increase in body weight (Figure 1(b)). To ensure whether the CS-induced mice model was established, we validated it through hematoxylin-eosin (H&E) staining and pulmonary function testing (Figure 1(c) and Supplementary Fig.S1). The H&E staining experiment revealed alveolar air-space enlargement and slight neutrophil infiltration in the lungs of these CS mice sacrificed on the 40th and 70th days after smoke exposure. In contrast, the degree of lung damage in the probiotic pre-treated group was significantly reduced compared to the CS group (Figure 1(c)).

On the 100th day of the smoking exposure, we conducted a comprehensive evaluation of lung function and histopathology to determine the severity of COPD. Our findings revealed that the mice exposed to CS developed prominent COPD characteristics, including respiratory distress, airflow limitation, alveolar collapse, congestion, and inflammatory cell infiltration (Figure 1(c), Black arrow). In contrast, the CS-exposed mice treated with SMM914 (SMM914CS) displayed significantly milder lung pathology, with only mild inflammation and alveolar wall enlargement (Figure 1(c,d)). To assess the airway dysfunction induced by cigarette smoke in the mouse model of COPD, we employed noninvasive measures, namely EF_50_ and Penh, which are commonly used indices of bronchoconstriction. EF_50_ is defined as the tidal flow at the midpoint (50%) of expiratory tidal volume and is negatively correlated with the degree of COPD.^27,28^ Penh (enhanced pause) is used as an indicator of bronchoconstriction in conscious mice.^29^ The results demonstrated that the CS group had significantly decreased EF_50_ and increased Penh values compared to the PBS group (Figure 1(e)). Notably, no significant airway obstruction was observed in the SMM914CS group (Figure 1(e)).

To further investigate the effects of SMM914, we analyzed the biomarkers of COPD in mouse serum. Tumor necrosis factor-alpha (TNF-α), Interferon-gamma (IFN-γ), and Interleukin-6 (IL-6), a pro-inflammatory cytokine, have been shown to correlate positively with the severity of COPD.^30^ In this study, we observed a significant upregulation of TNF-α and IFN-γ in the CS group, compared to the PBS group (Figure 1(f)). Also, we observed a significant reduction in the secretion of the anti-inflammatory factor interleukin-10 (IL-10) in the CS group compared to the PBS group (Figure 1(f)), indicating an imbalance in the inflammatory response. However, treatment with SMM914 yielded a different outcome. The SMM914CS group exhibited significantly decreased secretion of TNF-α and IFN-γ, along with increased production of the anti-inflammatory cytokine IL-10 compared to the CS group (Figure 1(f,g)). These findings confirmed the successful construction of CS-induced mouse model of COPD, suggesting that SMM914 effectively reduces the secretion of pro-inflammatory factors while promoting the release of anti-inflammatory cytokines, and alleviating COPD symptoms. Overall, these results highlight the potential of SMM914 as a therapeutic bacterium for modulating the inflammatory response and ameliorating COPD symptoms through the gut-lung axis.

### SMM914 ameliorates cigarette smoke-induced gut microbial dysbiosis

**Figure 2.**
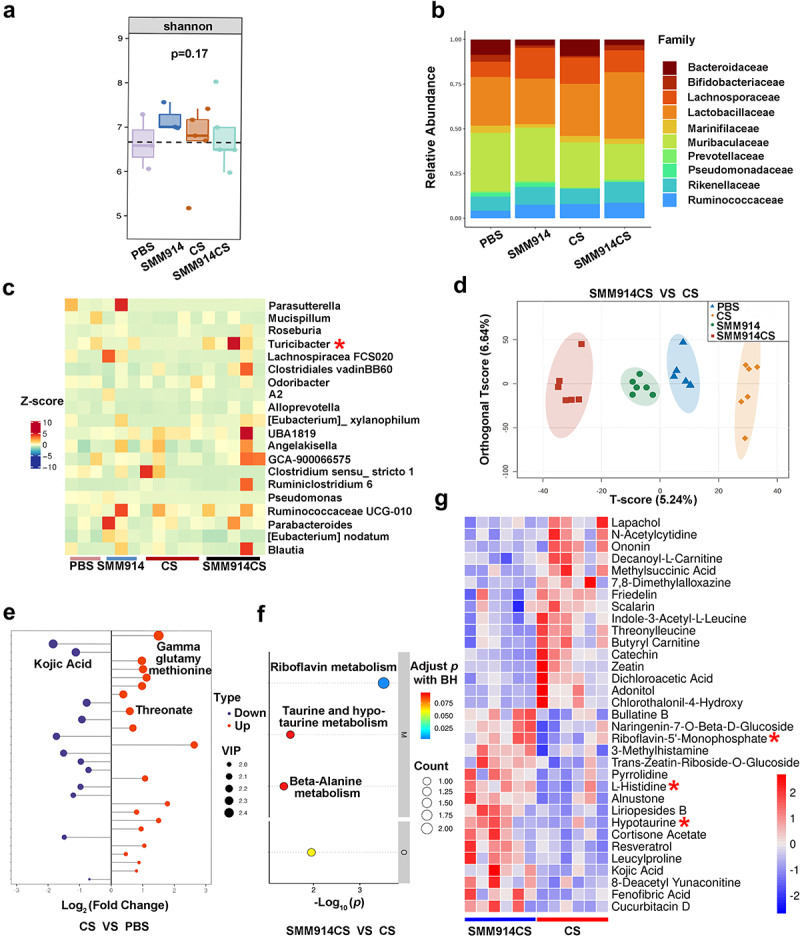
SMM914 provides protection to lung through balancing intestinal microbiota and regulating lung metabolism. (a) 16S rRNA sequencing analysis of stool samples at day 100 after CS exposure described in Figure 1(a). Graph depicts Shannon a-diversity index of grouped data. *N* = 5–6 mice per group. (b) Relative abundance of gut bacterial family in each group after CS exposure as described in Figure 1(a). *N* = 5–6 mice per group. (c) Bacterial genus altered by SMM914 in comparison with the CS group were shown in the random forest plot. (d) Score scatter plot from OPLS-DA model of PBS (*n* = 6), CS (*n* = 6), SMM914 (*n* = 5), SMM914CS (*n* = 6) of mice described in Figure 1(a). (e) Fold change plot of metabolic analysis for lung tissues from CS and PBS groups, respectively. (f) Bubble plot of metabolic pathway analysis for the lung tissues from SMM914CS and CS groups. (g) The hierarchical clustering heatmap of differentially metabolite expression of lungs between SMM914CS and CS groups.

At day 100 after smoke exposure, we conducted fecal 16S rRNA gene sequencing to investigate the gut microbiota in each experimental group and to shed light on the mechanism by which SMM914 reduces smoke-induced COPD. We analyzed the diversity of alpha, and the results demonstrated that the Shannon index of microbiota in the SMM914 group was significantly higher than that in other groups (Figure 2(a)). Two additional indices, Chao and Simpson, corroborate the heightened microbiota diversity in the SMM914CS group compared to the CS group (Supplementary Fig. S2A). The Principal Coordinates Analysis (PCoA) plot offers substantial evidence, demonstrating the impact of smoking treatment on microbiota alterations, with concurrent discernible effects from SMM914 treatment on beta diversity (Supplementary Fig. S2B). According to the analysis of the relative abundance of microbiota in mice, we found that the abundance of *Lachnospiraceae* in the gut of SMM914 group mice was significantly increased, which has potential to supply beneficial butyrate and acetate (Figure 2(b)). In contrast, compared with the SMM914CS group, the relative abundance of *Lactobacillaceae*, and *Bifidobacteriaceae* in the feces of CS group was greatly decreased, while the abundance of *Bacteroidaceae* showed an increasing trend (Figure 2(b)).

At the genus level, we observed significant differences between the experimental groups. Specifically, *Turicibacter* abundance was significantly higher in the SMM914CS group than in the CS group (Figure 2(c)). And, SMM914 treatment significantly increased the abundance of *Roseburia*, which produces SCFAs (Figure 2(c)).^31^ Furthermore, the analysis of intestinal metabolomics data revealed that oral administration of SMM914 to CS-exposed mice resulted in increased secretion of antioxidants such as zeatin, catechin, and isoliquiritigenin (Supplementary Fig. S2C). It may relate to the increased component of *Firmicutes* and *Bacteroidetes* (Supplementary Fig. S2D). This was found to be advantageous in safeguarding intestinal homeostasis and mitigating the detrimental effects of oxidative stress on the intestinal environment. We found significant differences in the intestinal bile secretion between the SMM914CS and CS groups, which is consistent with our 16S rRNA gene sequencing analysis (Supplementary Fig. S3a). Additionally, this augmentation stimulated the transformation of gut microbiota community structure to a potent antioxidant property. Taken together, these results suggest that SMM914 effectively mitigates CS-induced gut microbiota dysbiosis and reduces COPD symptoms by augmenting the abundance of SCFA-producing bacteria.

### SMM914 administration induces systemic elevation of pulmonary antioxidant levels

To further explore the molecular mechanism by which oral administration of SMM914 slowed the progression of COPD in mice, we used metabolomics analysis to detect changes in metabolites in the lungs of mice. In total, 80 compounds with significant differences were detected using orthogonal partial least squares discriminant analysis (OPLS-DA), highlighting the distinct metabolic profiles of the lung of each experimental group (Figure 2(d) and Supplementary Table S1). Threonate, a metabolite of ascorbic acid and aldaric acid metabolism, is upregulated following inflammation.^32^ Here, we observed a significant increase in threonate levels in the CS group compared to the PBS group (Figure 2(e)), indicating the presence of inflammation. Furthermore, the production of γ-glutamethionine, another indicator of inflammation,^33^ was significantly elevated in the CS group, while the antioxidant Kojic Acid was reduced (Figure 2(e)). In contrast, probiotic treatment resulted in a significant increase in Kojic Acid levels compared to the CS group, indicating an improvement in oxidative balance (Figure 2(e)).

The metabolic pathway analysis showed that the riboflavin metabolism of SMM914CS group was significantly altered compared to the CS group, as evidenced by the significant increase in the production of Riboflavin-5’-Monophosphate (FMN) (Figure 2(f, g)). Riboflavin metabolism is a key component of the mitochondrial respiratory chain, and the increase in the amount of FMN catalyzes redox reactions and improves oxidative stress.^34^ According to the mass spectrometry analysis, SMM914 could secrete 3-sulfino-L-alanine and taurine (Supplementary Table S2). Also, we observed differences in taurine and hypotaurine metabolism, with hypotaurine being more abundant in the SMM914CS group (Figure 2(f)). A significant increase in L-histidine expression in the SMM914CS group, has been shown to scavenge hydroxyl radicals and singlet oxygen (Figure 2(g)).^35^ These findings suggest that SMM914 upregulates the expression levels of endogenous antioxidants such as L-histidine, FMN, and hypotaurine, and regulates bile acid balance, resulting in reduced levels of oxidative stress in the body and alleviation of CS-induced lung damage in mice, which attributed to SMM914‘s antioxidant properties and its ability to regulate the gut microbiota.

### Oral gavage of SMM914 mitigates the progression of ozone-induced COPD

**Figure 3.**
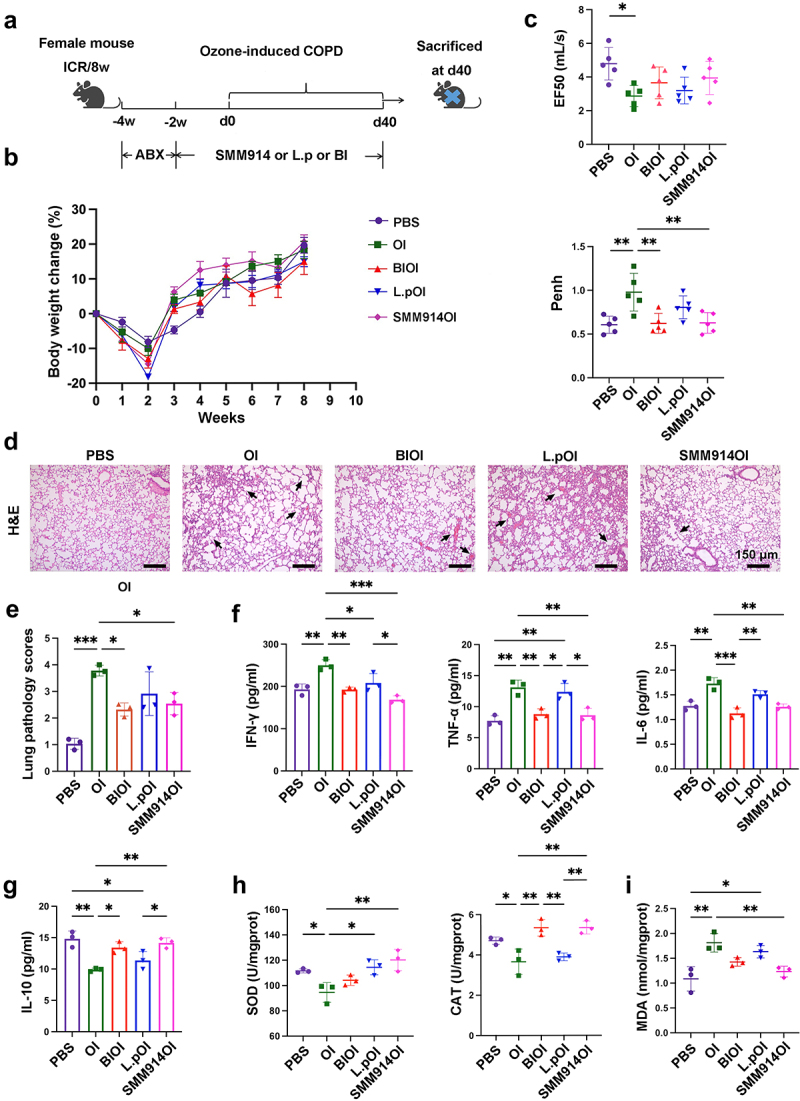
Pretreatment with the probiotic SMM914 attenuated OI-COPD. (a) Schematic diagram of the experimental system: mice received an oral gavage of normal saline (PBS and OI group), BIOI, or SMM914OI or L.pOI for 14 weeks (1 × 10^9^ CFU/twice a week) prior to being subjected to ozone treatment. *N* = 8–10 mice per group. (b) Body weight change percentage. *N* = 8–10 mice per group. (c) Lung function analyses including Penh and EF_50_ were measured after 40 days. (d) The representative images of lung histopathology. The black arrows indicate damaged areas, characterized with denatured and collapsed epithelial cells, thickened alveolar septa, alveolar damage, and activated inflammatory cell infiltration. *N* = 3. (e) Histological scoring of lung tissues was performed in a blinded manner according to INHAND. (f) Serum TNF-α, IFN-γ and IL-6 concentrations after 40 days of ozone treatment in the four groups described in (a). *N* = 3. (g) Serum IL-10 concentrations after 40 days of ozone treatment in the four groups described in (a). *N* = 3. (h) Concentrations of the antioxidant index (SOD and CAT) in the lung from each group at day 40 after OI exposure of mice. *N* = 3. (i) Concentrations of the MDA in the lung from each group at day 40 after OI exposure of mice. *N* = 3.

Due to the complex composition of cigarette smoke, CS-induced modeling of COPD does not solely rely on oxidative stress-induced reactions. Therefore, in order to reveal whether SMM914 alleviates the progression of COPD in mice models through antioxidant activity, we developed a single-factor-induced COPD model using ozone as a strong oxidizing agent. We employed ABX treatment to deplete the resident bacteria in 6 to 8-week-old female ICR mice for two weeks, followed by two weeks of oral SMM914, and then six weeks of ozone exposure (Figure 3(a)). Simultaneously, in order to assess whether probiotics with antioxidant properties possess the capability to attenuate the progression of COPD akin to SMM914, we employed *Lactiplantibacillus plantarum subsp. plantarum* (ATCC 14,917) (L.p) as the control in this experiment. L.p is a common lactic acid bacterium, which is widely used as a natural antioxidant for the removal of reactive oxygen species and free radicals, and oxidative damage repair.^36,37^ Weight changes in each group of mice were monitored throughout the study. We found that the weight of each group of mice decreased after ABX treatment (Figure 3(b)). As the COPD modeling process progressed, the weight changes of PBS and the SMM914 pretreated ozone-induced (SMM914OI) group were almost identical, and the change rate of the ozone-induced (OI) group was slightly lower than that of the previous two groups, while the weight changes of L.p pretreated ozone-induced (L.pOI) group and the Budesonide and Ipratropium bromide pretreated ozone-induced (BIOI) group were less (Figure 3(b)). Next, we utilized lung function tests and H&E staining to assess the lung lesions triggered by ozone in mice. Our analysis revealed that the EF_50_ value of the OI group was significantly lower than that of the PBS group, and the Penh value of the OI group was notably higher than those of the PBS, BIOI, and SMM914OI, indicating the manifestation of COPD-like symptoms, such as respiratory distress and airway obstruction, in CS group mice (Figure 3(c)). Significantly, interventions with SMM914 and BI significantly ameliorated these symptoms, and no significant difference in airway responsiveness between SMM914OI and BIOI (Figure 3(c)). And, we compared the respiratory curves, and saw a more pronounced respiratory resistance in the L.pOI and OI groups (Supplementary Fig. S3B). To further evaluate the degree of lung pathological changes, we analyzed H&E stained lung sections (Figure 3(d) and Supplementary Fig. S3C). Our results revealed that the OI group mice exhibited thickened lung interstitium, collapsed and fused alveolar walls, and inflammatory cell infiltration (Figure 3(d), Black arrow). In contrast, the SMM914OI group showed lower levels of pathological inflammatory cell infiltration and alveolar collapse compared to the L.pOI group, similar to that of the BIOI group (Figure 3(d,e)).

To better comprehend the inflammatory levels in each group of mice, we conducted enzyme linked immunosorbent assay (ELISA) tests for inflammatory markers in the serum of the mice mentioned above. After ozone induction, the inflammatory cytokines IL-6, TNF-α, and IFN-γ significantly increased in the blood. Among them, the expression level of TNF-α and IL-6 was significantly higher than in the PBS, BIOI, and SMM914OI group, and IFN-γ in the OI group was significantly higher than in other groups (Figure 3(f)). In other words, BI and SMM914 exhibited a superior inhibitory effect on the expression of inflammatory cytokines induced by ozone treatment, in comparison to L.p. In terms of the anti-inflammatory cytokine IL-10, the expression of IL-10 in the two groups treated with SMM914 and BI increased compared with the OI group has significant differences, and did not reach significant differences compared with the PBS group (Figure 3(g)). These findings confirm the potent anti-inflammatory abilities of SMM914 and BI, which aligns with the conclusions drawn from the CS-induced COPD mouse model. Moreover, we investigate the levels of main antioxidant enzymes, including Superoxide dismutase (SOD) and catalase (CAT), as well as the marker of lipid peroxidation, malondialdehyde (MDA). These biomarkers offer insights into the alterations occurring within the internal oxidative defense system. The results showed that oral probiotics significantly up-regulated the secretion of SOD in comparison with the OI group, but BI did not improve the enzyme expression (Figure 3(h)). Concurrently, SMM914 and BI treatment significantly upregulated the expression of CAT, whereas L.p. therapy exhibited comparable levels to the OI group, demonstrating no significant differences (Figure 3(h)). These results indicate that oral administration of SMM914 has the potential to resist the production of free radicals caused by mitochondrial damage through enhancing the expression of antioxidant enzymes (Figure 3(h)). In addition, the measurement of MDA content serves as an indicator of cellular damage and aging.^38^ Our findings revealed a significantly higher concentration of MDA in the OI group compared to the PBS and SMM914OI groups (Figure 3(i)). Interestingly, both BI and L.p treatments did not alleviate the elevation of MDA induced by zone treatment (Figure 3(i)). These results suggest that oral administration of SMM914 contributes to the reduction of lipid peroxide levels. These findings provide compelling evidence that oral administration of SMM914 confers substantial antioxidant effects in mice, thereby offering significant protection against oxidative stress-induced damage. Taken together, we confirm that SMM914 has similar therapeutic effects to the current clinical drugs ipratropium bromide and budesonide and could be used as a live probiotic preparation to modulate the progression of COPD.

## SMM914 alters gut microbiota composition toward high antioxidant activity in ozone-exposed mice post-ABX treatment

**Figure 4.**
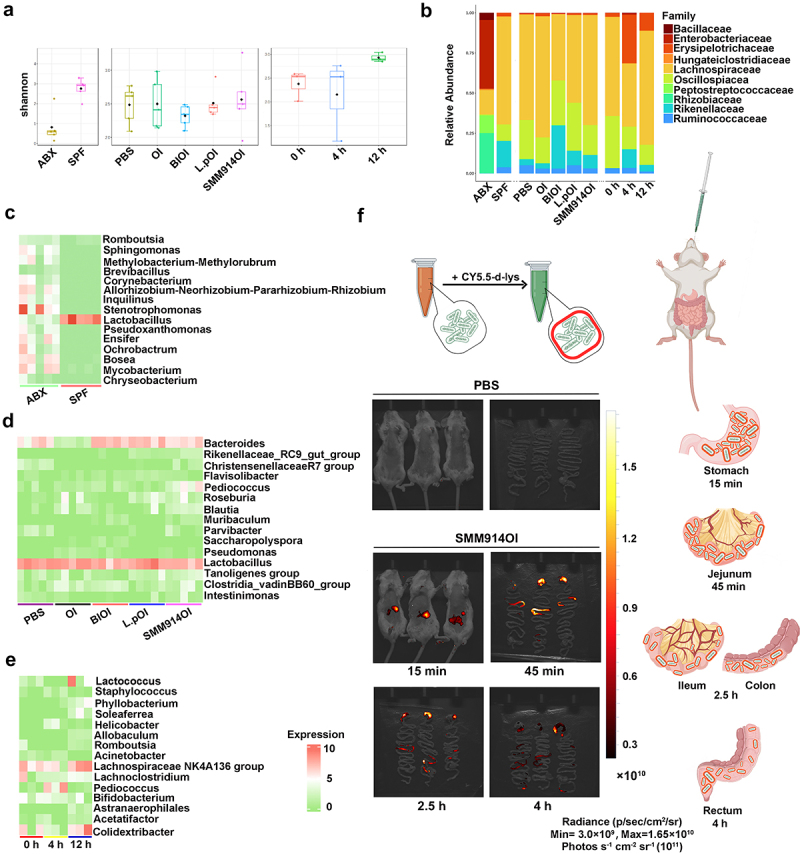
SMM914 alters the intestinal microbiota of the mice with ABX treatment. (a) 16S rRNA sequencing analysis of stool samples at day 40 after OI exposure described in Figure 3(a) graph depicts Shannon a-diversity index of grouped data. *N* = 3–5 mice per group. (b) Relative abundance of gut bacterial family in each group after OI exposure as described in Figure 3(a). *N* = 3–5 mice per group. (c) Bacterial genus altered by ABX treatment in comparison with the PBS group were shown in the random forest plot. (d) Bacterial genus altered by OI exposure and BI, SMM914, and L.P treatment in comparison with PBS were shown in the random forest plot. (e) Bacterial genus changed in four-time points shown in the random forest plot. (f) Live imaging of mice after orally administrating SMM914 at 15min, 45min, 2.5h and 4 h. (the schematic diagram on the right side illustrates the temporal details of SMM914 in the mouse intestine at various time points).

To further explore the effects of SMM914 on intestinal microbes and to reduce the impact of native intestinal microbiota on disease development in mice, we used the ABX treatment. The alpha diversity of microbiota in the feces of mice was analyzed. Shannon and Chao indices revealed a significant reduction in bacterial diversity after two weeks of ABX treatment. Furthermore, the ABX group demonstrated a significant reduction in the Simpson index, indicating the pronounced depletion of dominant gut microbes (Figure 4(a), Supplementary Fig. S4A). Subsequent beta diversity analysis affirmed the observable shifts and depletion within the native symbiotic microbial community following ABX treatment (Supplementary Fig. S4B). The dominant family of microbiota in mouse feces of the ABX group shifted from *Lachnospiraceae* to *Enterobacteriaceae*, *Rhizobiaceae*, and *Peptostreptococcaceae* compared to the pre-treatment ones (Figure 4(b)). Concurrently, the random forest plot illustrated an increase in the abundance of inflammation-inducing detrimental bacterial families such as *Romboutsia*, *Chryseobacterium* and a significant reduction in *Lactobacilli* in the gut post-antibiotic treatment (Figure 4(c)). This disruption of the endogenous gut homeostasis led to a weakened barrier function and increased risk of inflammation. The analysis revealed that the Shannon index data in the SMM914OI group was lower than that in the PBS group but higher than in the remaining three groups (Figure 4(a) and Supplementary Fig. S4B). Next, by comparing random forest plots we found that the abundance of *Bacteroides* was significantly low in the OI group without treatment. *Rikenellaceae_RC9_gut_group, Pediococcus*, *Intestinimonas*,^39,40^ and other SCFA-producing bacteria were enriched in the probiotic treatment group, in which SMM914 had the advantage over the L.p (Figure 4(d)). After the oral administration of SMM914, as the abundance of *Pediococcus* increased, the production of bacterial acidic metabolites mounted up, indicating the further acidification of the gut environment, which suppressed the growth of harmful bacteria and promoted the growth of other bacteria that secrete butyrate and propionate, thereby maintaining the balance of the gut microbiota (Supplementary Fig. S4C).

### SMM914 demonstrates a short in vivo half-life

In order to investigate the effective duration and colonization ability of SMM914 in mice, we utilized *in vivo* imaging to track the bacteria’s distribution in the intestinal tract of live animals. We orally administered Cy5.5-d-lys-labeled SMM914 to mice and immediately performed fluorescence imaging under anesthesia. Compared to the control group, the fluorescence-labeled SMM914 emitted a significant fluorescence signal in the stomach of nude mice. To more accurately evaluate the distribution of SMM914 in the mouse gastrointestinal tract after oral administration, we performed fluorescence imaging of the gastrointestinal system after dissecting the mice. The results showed that 45 minutes after oral administration, the total amount of bacteria mainly stayed in the jejunum. After 2.5 hours later, the fluorescence signal was concentrated in the ileum and colon. By 4 hours, the fluorescence signal mainly existed in the ileum, indicating that the bacteria were about to be excreted with the feces (Figure 4(f)).

To further elucidate the residence time of SMM914 *in vivo*, fecal samples were collected at 0, 4, and 12 hours post oral administration for subsequent 16S rRNA sequencing. The obtained data revealed a pronounced increase in the alpha diversity of the microbiota following the administration of SMM914 in mice (Figure 4(a) and Supplementary Fig. S4A). Analysis through the PCoA plot indicated significant differences between samples from the group treated with SMM914 at 4 hours and the 0-hour group, with this effect diminishing by the 12-hour time point (Supplementary Fig. S4B). The abundance of the dominant family *Lachnospiraceae* decreased and then recovered (Figure 4(b)). The random forest plot showed that approximately 4 hours after oral administration, the abundance of *Pediococcus* significantly increased, while around 12 hours, its abundance decreased significantly (Figure 4(e)). Our previous research results also showed that 16S rRNA sequencing could not detect SMM914 in mouse feces 24 hours after administration.^26^ These results indicate that orally administered SMM914 can persist in the mouse body for approximately 12 hours and is entirely eliminated from the body 24 hours later. Therefore, SMM914 is a short-acting, biologically safe, and non-recombinant probiotic that can be used for long-term health maintenance or disease treatment.

## SMM914 induces upregulation of antioxidant gene expression in the lungs, mitigating ozone-induced oxidative stress

**Figure 5.**
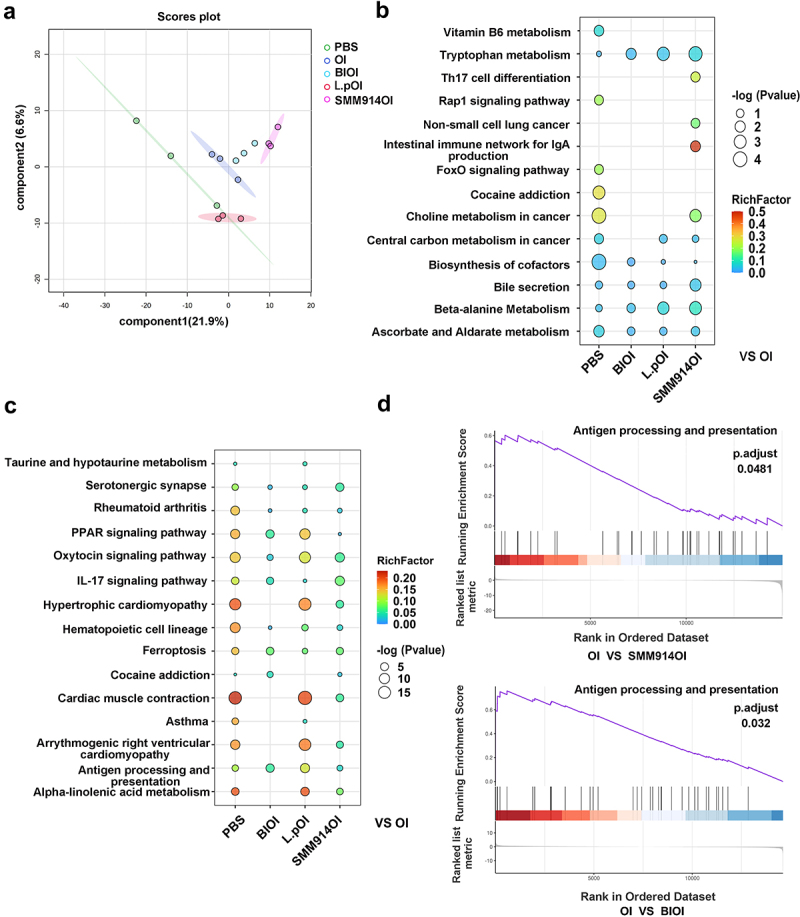
SMM914 decelerates the progression of COPD through modulation of metabolites and gene expression. (a) Score scatter plot from OPLS-DA model of PBS (*n* = 3), OI (*n* = 3), BIOI (*n* = 3), L.POI (*n* = 3), SMM914OI (*n* = 3), of mice described in Figure 3(a). (b) Bubble plot of metabolic pathway analysis for each group in Figure 3(a). The color depth and bubble size indicate ln (p) values and the impact of the pathway. (c) Bubble plot of transcriptomic pathway analysis for each group in Figure 3(a). The color depth and bubble size indicate ln (p) values and the impact of the pathway. (d) Gene set enrichment analysis of OI, SMM914OI, and BIOI.

To further analyze whether SMM914 can alleviate pulmonary oxidative stress caused by OI, we conducted metabolomics analyses on the lung tissues of the five groups of mice mentioned above. The OPLS-DA score plot showed significant differences in metabolites in the lungs of the five groups of mice (Figure 5(a)). The ozone exposure significantly decreased the production of Xanthunenic Acid, which is the tryptophan secondary metabolite (Supplementary Fig. S4D). Compared to the PBS group, ozone treatment significantly increased the levels of several tumor metabolic pathways, such as choline metabolism in cancer, central carbon metabolism in cancer, and greatly altered the secretion of biosynthetic accessory factors in the mice (Figure 5(b)). Additionally, the expression of glutathione in SMM914OI group mice was significantly upregulated in comparison to the OI group mice. Moreover, owing to the effects of ozone treatment on the gut microbiota, choline and endogenous metabolites were abnormally metabolized in the lung tissue of OI group mice (Supplementary Fig. S5A, B). Among the significantly ranked metabolic pathways, we observed that the tryptophan pathway and the bile secretion were significantly increased in the SMM914OI group, while the metabolites of cancer-related pathways (small cell lung cancer, non-small cell lung cancer, gastric cancer) were significantly downregulated due to the intervention of this probiotic (Figure 5(b)). However, the first-line clinical drugs ipratropium bromide and budesonide were observed to significantly reduce the risk of cancer in the ozone-treated mice but did not increase the expression of antioxidant metabolites (such as glutathione) (Supplementary Fig. S5B). Combining 16s rRNA sequencing, ELISA, and metabolomics analysis, we found that L.p treatment did not reduce the oxidative imbalance caused by ozone exposure. While L.p treatment increased the abundance of SCFAs-producing bacteria (Figure 4(d)), it did not inhibit the release of pro-inflammatory factors or increase the expression level of antioxidants (Figure 3(e,f)).

To elucidate the molecular mechanisms underlying SMM914’s efficacy in reducing ozone-induced lung damage in mice, we performed transcriptomic analysis on lung tissue. By comparing gene expression profiles, we found that genes closely related to the progression of COPD,^41^ such as *HHIP*, *RIN3*, *GSTCD*, and *NPNT*, along with *HIF-1α*, a gene related to oxidative stress, were significantly upregulated in the lungs of the mice after 6 weeks of ozone exposure (Supplementary Fig. S5C). However, the expression of these genes was significantly downregulated in the SMM914-treated OI group (Supplementary Fig. S5C). Mapping these genes to the function pathway revealed a significantly increased risk of COPD comorbidities such as asthma, rheumatoid arthritis, hypertrophic or dilated cardiomyopathy, and arrhythmogenic right ventricular cardiomyopathy in OI mice (Figure 5(c)). Meanwhile, the peroxisome proliferator-activated receptor (PPAR) pathway and alpha-linolenic acid metabolism were upregulated, indicating activation of the body’s antioxidant pathways and confirming changes in oxidative levels. KEGG enrichment pathway analysis showed that ozone affects the occurrence of ferroptosis in lung cells and the production of hematopoietic cell lineage (Figure 5(c)). Compared to the PBS group, the expression of *SCL7a11* was abnormally increased in the OI group (Supplementary Fig. S5C), and its overexpression could inhibit ferroptosis but promote the development of lung cancer.^42^ Additionally, OI treatment increased the expression of *CXCL1*. However, in the SMM914OI group, the expression of *SCL7a11* and *CXCL1* was significantly reduced (Supplementary Fig. S5C). Compared to PBS-treated mice, SMM914 administration increased the expression pathway of taurine and hypotaurine (Figure 5(c)), which play an antioxidant role and was consistent with our findings in the CS-induced COPD model (Figure 2(g)). Moreover, the probability of COPD comorbidity risk in the SMM914OI group was lower than that in the OI group, which may be related to the activation of the oxytocin signaling pathway (Figure 5(c)). Additionally, SMM914 may inhibit lung inflammation development by downregulating the expression of the *IL-17ra* gene (Supplementary Fig. S5C). Next, SMM914 administration was found to increase tryptophan metabolites, promote serotonin generation, and further convert to the strong antioxidant 6-Hydroxymelatonin (Supplementary Fig. S5D). Therefore, the *in vitro* experiments were conducted to elucidate the impact of SMM914 on the production of tryptophan pathway metabolites under oxidative stress. Mass spectrometry analysis revealed the generation of L-tryptophanamide and 5-hydroxy-L-tryptophan by SMM914, as outlined in Supplementary Table S2. These metabolites serve as substrates for increasing 6-hydroxymelatonin. Utilizing 5-hydroxy-L-tryptophan as a substrate and employing dopa decarboxylase (DDC) as a catalyst, we introduced serum from both normal and COPD mice to observe variations in serotonin production (Supplementary Fig. S6A-D). Serotonin, a precursor for 6-hydroxymelatonin via the melatonin pathway, exhibited a gradual increase over time in the group with the serum of healthy mice and SMM914 (Supplementary Fig. S6A-D). Notably, a significant disparity in serotonin production emerged between the two groups receiving serum from healthy and COPD mice. SMM914 exhibited the capacity to ameliorate the deleterious effects induced by inflammatory cytokines present in the serum from the mice with COPD (Supplementary Fig. S6A-D). Despite the incubation of SMM914 with COPD mouse serum failing to elevate serotonin levels within the initial hour due to diminished production caused by inflammatory cytokines, a noteworthy observation emerged after 3 hours. The proliferation of the bacterium facilitated an increased availability of substrates, subsequently contributing to the synthesis of serotonin with time extension (Supplementary Fig. S6A). The results suggest that supplementation with SMM914 not only augmented serotonin production but also alleviated the suppressed activity of the DDC enzyme in an inflammatory environment, substantiating the *in vivo* therapeutic effects of SMM914 in alleviating COPD. In comparison with SMM914, L.p was not effective in reducing the risk of COPD comorbidities, and the PPAR pathway remained highly activated in the body (Figure 5(c)). Furthermore, GSEA enrichment pathway analysis indicated that both antigen presentation and processing pathways were significantly altered in the SMM914OI and BIOI groups of mice (Figure 5(d)). *Hspa1a* and *Hspa1b*, as major genes in these pathways, showed a slightly higher trend of inhibition in the BIOI and SMM914OI groups compared to the OI and SMM914OI groups (Supplementary Fig. S5C).

### SMM914 decreases macrophage M1 polarization

**Figure 6.**
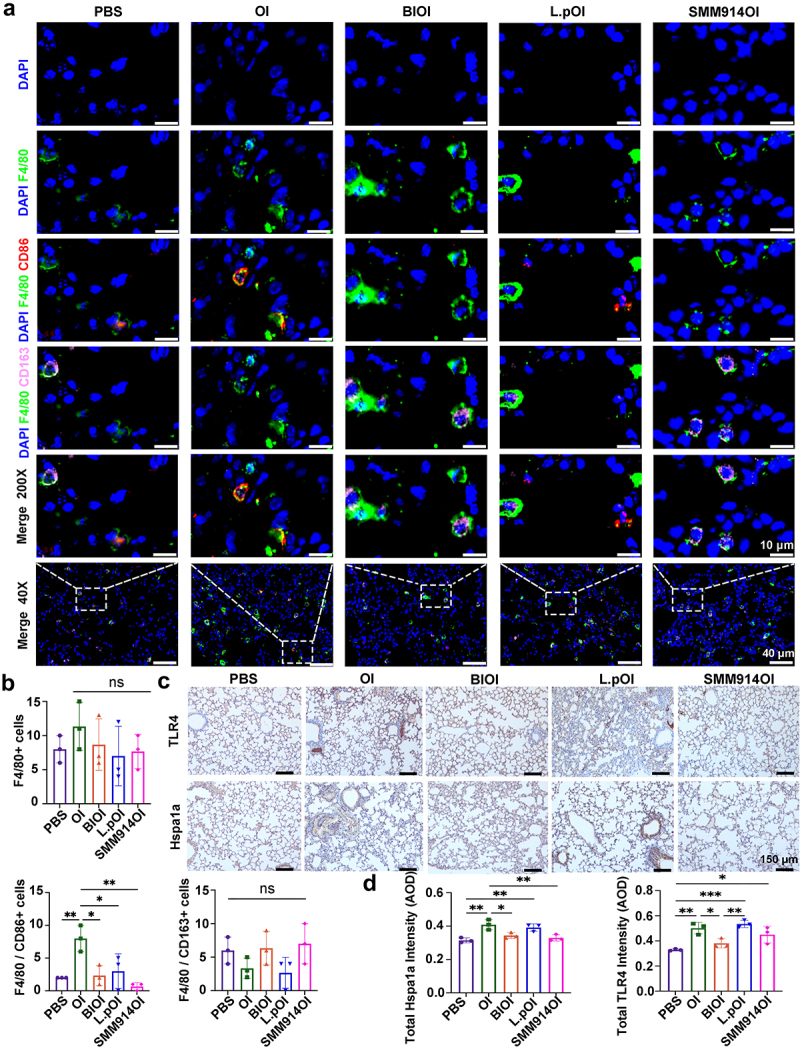
SMM914 attenuates M1 macrophage polarization in the lung. (a) Immunofluorescence staining analysis of M1 (DAPI/F4/80/CD86) and M2 (DAPI/F4/80/CD163) macrophage polarization in each group from Figure 3(a). *N* = 3. (b) Quantification of immunofluorescence staining of macrophages polarized in different directions (F4/80+ cells, F4/80 CD86+ cells, F4/80 CD163+ cells). *N* = 3 (c) Representative IHC staining of Hspa1a and TLR4 in lung sections from five Ozone-induced mice groups (*n* = 3 per group) with corresponding quantification (d). AOD, average optical density.

The number and activity of macrophages increase in the lung tissue of COPD patients, so we used immunofluorescence staining to clarify the effect of oral administration of SMM914 on macrophage polarization in the COPD model. We labeled all macrophages in the mouse lungs with F4/80 and found that the number of recruited macrophages in the OI and L.pOI groups was significantly higher than that in the BIOI and SMM914OI groups, consistent with the results of H&E staining showing significant inflammatory cell infiltration in the OI and L.pOI groups (Figures 3(d) and 6(a)). Furthermore, we used CD86 and CD163 to label M1 and M2-related macrophages, respectively. We observed F4/80/CD86 and F4/80/CD163-positive cells by immunofluorescence. The results showed that M1 pro-inflammatory macrophages activated by F4/80/CD86 were increased in the lungs of the OI group mice (Figure 6(a,b) and Supplementary Fig. S7A, S8A). The increase in the inflammatory response of the body leads to the activation of M1 pro-inflammatory macrophages in the pulmonary alveolar macrophages surrounding blood vessels and bronchi after stimulation by pro-inflammatory factors from bone marrow through blood delivery to the lungs. However, after treatment with BI and SMM914, the overexpression of Hspa1a and Hspa1b was significantly inhibited, and the polarization of macrophages around blood vessels and bronchioles toward M1 pro-inflammatory type was reduced, which was consistent with the results of transcriptome analysis (Supplementary Fig. S5C). Through immunohistochemical staining (IHC), we confirmed that ozone induction increased the expression of Hspa1a and TLR4 in mice (Figure 6(c,d) and Supplementary Fig. S9A, B). At the same time, the upregulation of Hspa1a promoted the differentiation of M1-type macrophages, which is closely related to the severity of inflammation in lung tissue. It is worth noting that although BI treatment reduced the risk of complications of COPD and improved anti-inflammatory cytokine expression by regulating macrophage differentiation, metabolomics analysis revealed that this therapy may have the potential to induce drug addiction and other cancers, increasing the prognostic risk of COPD. Combined analysis of the above non-targeted metabolomics data and transcriptomics confirmed that SMM914 mainly regulates the lung inflammation environment by jointly regulating the tryptophan pathway, bile acid pathway, and antigen presentation and processing pathway, thereby reducing oxidative stress damage to mouse lung tissue.

### Decreased antioxidant enzymes in COPD patients

**Figure 7.**
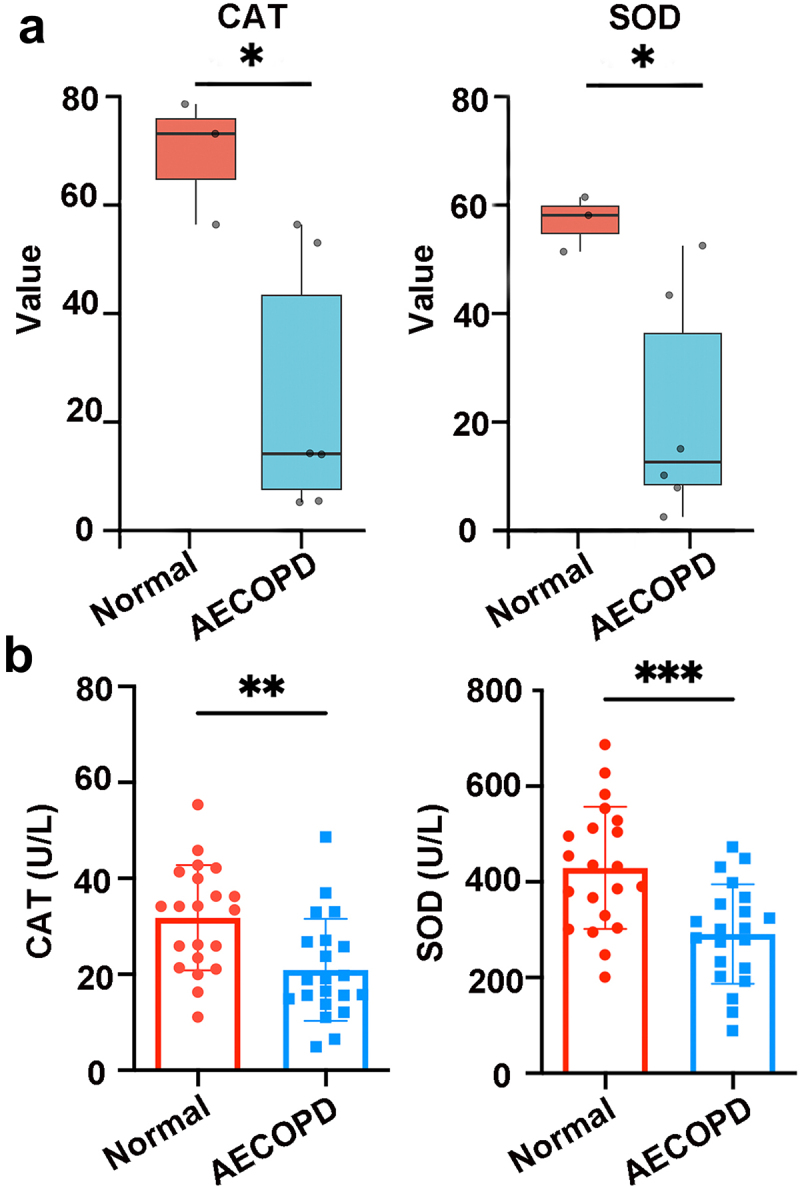
Oxidative stress is clinically relevant in COPD. (a) Values of CAT and SOD in the serum from the database. *N* = 3–6 individuals per group. (b) Concentrations of the antioxidant index (CAT and SOD) in the serum from the normal and AECOPD group. *N* = 21.

In order to evaluate the clinical relevance of our discoveries and validate the role of oxidative stress in COPD, we initially focused on a representative sample of 9 cases obtained from the NCBI – SRA (National Center for Biotechnology Information – Sequence Read Archive) database platform (project number: PRJNA853498). This serum sample consisted of 3 healthy individuals and 6 patients experiencing acute exacerbations of COPD (AECOPD). Our analysis revealed a significant decline in the levels of superoxide dismutase (SOD) and catalase (CAT) during the advanced stages of COPD disease (Figure 7(a)). To further strengthen our findings, we conducted oxidative stress assays using 42 serum samples collected from the Affiliated Jiangning Hospital of Nanjing Medical University. This collection comprised 21 healthy individuals (designated as the “Normal” group) and 21 patients with AECOPD. Consistent with the mouse model of COPD, our results exhibited significantly decreased expression of SOD and CAT in patients with AECOPD (Figure 7(b)). These findings collectively underscore the close association between oxidant stress and the progression of COPD (Figure 3(g)), indicating that increasing the expression of antioxidant enzymes, such as using probiotics, has the potential to alleviate the development of COPD.

## Discussion

In this study, we elucidate the crucial role of probiotic supplementation in mitigating oxidative stress-induced inflammation within the lungs. Employing SMM914, we orchestrated a modulation of the gut microbiota balance in mice with COPD, induced by exposure to either cigarette smoke or ozone. This intervention led to a significant augmentation in the population of beneficial bacteria, producing essential SCFAs and antioxidant substrates. Additionally, it resulted in an elevation of antioxidant enzymes, including SOD and CAT. Concurrently, the antioxidant metabolites produced by SMM914 facilitated a marked elevation of endogenous antioxidants, such as hypotaurine and 6-hydroxymelatonin, within the lung microenvironment. These orchestrated molecular changes collectively bolstered intrinsic antioxidant defenses, thereby fortifying the respiratory system against inflammation. Importantly, this intervention yielded a significant amelioration in the development of COPD in the murine model.

Actually, a large body of research has demonstrated the existence of the gut-lung axis, in which the microbiota in the gut can communicate with organs in both the proximal and distal regions, and exchange metabolites, and regulate the transfer of immune cells.^19,20^ Our analysis of 16S rRNA abundance data indicates that oral administration of SMM914 significantly enriches beneficial bacteria, including *Lachnospiraceae*, *Rikenellaceae_RC9_gut_group*, *Roseburia*, and *Turicibacter*, in the murine gut. *Lachnospiraceae* and *Rikenellaceae_RC9_gut_group*, recognized as primary short-chain fatty acid producers,^43,44^ have demonstrated the ability to generate metabolites beneficial to the host. The increase in *Turicibacter* and *Roseburia* levels is associated with a reduction in harmful bacteria, reinforcing the intestinal mucosal barrier. Specifically, *Turicibacter*, a *Clostridia* class member, exhibits anti-inflammatory properties, conferring resistance to inflammation in inflammatory bowel disease models, secreting metabolites involved in host bile acid metabolism, and regulating immunity.^45,46^ The SMM914-induced accumulation of these probiotics promotes harmful bacteria depletion and anti-inflammatory cytokine secretion, ultimately alleviating COPD progression.

Further, our previous work had confirmed the antioxidant potential of SMM914.^26^ Plasma metabolomics indicated that it can activate the Nrf2-Keap1 antioxidant signaling pathway in piglets, regulate cysteine and methionine metabolism as well as lipid metabolism, and significantly reduce piglet mortality that caused by oxidative stress. Building upon these insights, we designed CS-induced harnessed this unique antioxidant properties of SMM914 to modulate the progression of COPD, a condition closely associated with heightened oxidative stress and a notable reduction in antioxidant enzyme expression.^47^ The observed effects of SMM914 can be attributed, in part, to its inherent capacity for the high production of antioxidant substrates, encompassing but not limited to L-tryptophanamide, 5-hydroxy-L-tryptophan, 3-sulfino-L-alanine, and taurine. In the tryptophan-melatonin pathway, L-tryptophanamide undergoes catalysis by tryptophanamidase to yield tryptophan, while 5-hydroxy-L-tryptophan acts as a precursor to serotonin. After oral administration of SMM914 in mice, the DDC enzyme catalyzes the conversion of 5-hydroxy-L-tryptophan to serotonin, creating favorable conditions for the synthesis of 6-hydroxymelatonin (Supplementary Fig. S6A-D). Recent research has revealed that the activity of the DDC enzyme is responsive to inflammatory stimuli, displaying a negative correlation with the levels of ROS in the surrounding environment.^48^ SMM914 could enhance DDC enzyme activity, leading to an increased production of serotonin (Supplementary Fig. S6A-D). This effect ameliorates the inhibition of the tryptophan metabolism pathway in the inflammatory environment. Another metabolite synthesized by SMM914, 3- sulfino-L-alanine, acts as a precursor to hypotaurine. Hypotaurine, known for its robust antioxidant properties, has been shown to regulate the expression of antioxidant enzymes in mice.^49^ Oral administration of SMM914 serves as a dual mechanism, providing both antioxidant precursors and increasing levels of intermediates. This dual action acts as a source for synthesizing endogenous antioxidants, effectively reducing oxidative stress.

To further elucidate the potential mechanisms underlying the antioxidant effects of SMM914, we employed ABX treatment to deplete intestinal bacteria prior to the intervention. ABX treatment is a well-established approach widely used to investigate how specific probiotics influence disease progression.^50,51^ While ABX treatment may not completely eradicate the entire intestinal microbiota, the use of mice with normal immune functions and intact organs provides a more realistic approximation of the treatment environment compared to germ-free mice, which lack this physiological context. Our investigations demonstrated that SMM914 effectively mitigated lung tissue damage in a COPD mouse model by downregulating oxidative stress-related genes, including *HHIP*, *RIN3*, *GSTCD*, *NPNT*, and *HIF-1α*. Moreover, SMM914 also markedly reduced the expression of CXCL1, a chemotactic factor responsible for guiding neutrophils to inflamed lungs and initiating an immune response.^52^ This reduction was associated with a decrease in neutrophil infiltration in lung tissues following exposure to ozone and CS in the SMM914-treated group (Figures 1(c) and 3(d)). Notably, SMM914 treatment significantly elevated levels of glutathione and 6-hydroxymelatonin, which are integral components of the body’s natural defense mechanisms against oxidative stress. These findings further underscore the potential of SMM914 against oxidative stress (Supplementary Fig. S4B and S4D). In addition, key enzymes in the antioxidant defense system, such as SOD and CAT, play a critical role in scavenging free radicals and protecting cellular structures. Simultaneously, the level of malondialdehyde (MDA) serves as an indicator of cellular damage and aging.^38^ The data of these biomarkers provide important insights for us into the adaptive response of the body to oxidative stress and cellular damage observed in COPD. Our study confirmed that SMM914 treatment significantly enhanced the expression of SOD and CAT, while effectively suppressing the elevation of MDA levels. We believe that the advantageous metabolites produced by SMM914, coupled with its regulatory effects on intestinal microbiota, alleviate oxidative stress-induced damage in the lungs through the gut-lung axis.

Additionally, some studies have shown that microbial involvement in the metabolic regulation of the body is also dependent on the activation of innate immune cells.^51,53,54^ Macrophages are one of the major immune cells involved in the development of COPD, playing an important role in clearing inflammatory cells, secreting cytokines, and delaying disease progression. Hspa1a regulates macrophage-related immune responses by activating the NLRP3 inflammasome.^55^ After OI exposure, the expression of Hspa1a and Hspa1b was significantly upregulated, which stimulated monocytes to express inflammatory cytokines IL-6 and IL-8 by triggering TLR2 and TLR4 receptors, which are closely associated with the severity of inflammation in lung tissue.^4,30^ Here, after oral administration of SMM914 or BI treatment, Hspa1a was significantly reduced. Inhibiting Hspa1a can reduce the degree of oxidative stress damage to the body caused by inflammatory reactions in macrophages during COPD development.^56^ At the same time, TLR activation was suppressed, the expression of the anti-inflammatory cytokine IL-10 was increased, and the probability of alveolar macrophages differentiating into M1 pro-inflammatory macrophages was reduced.^57^ Therefore, inhibition of macrophage polarization toward M1 type, and preventing the activation of Hspa1a, Hspa1b and TLR expression may be potential therapeutic targets for delaying COPD.

In summary, our study provides novel insights into the association between COPD progression and intestinal microbiota disorder, shedding light on the potential mechanisms underlying the cross-talk between the gut and lung. Specifically, we demonstrate that the oral administration of live probiotics, such as SMM914, yields significant benefits in mitigating the progression of COPD. This effect is achieved through the reconfiguration of the intestinal microbial structure and modulation of gut microbiota-derived metabolites, ultimately resulting in the alleviation of oxidative stress in lung tissue and immune cells. Our findings pave the way for a novel avenue in the development of active bacterial therapy, aimed at enhancing the endogenous antioxidant capacity as a means to mitigate oxidative stress-associated diseases.

## Limitations of the study

Several limitations exist in our study. Firstly, we were unable to identify a key pathway or specific metabolites that predominantly contribute to the antioxidant effects of SMM914 in COPD treatment. The mechanisms underlying the favorable effects of probiotics, such as SMM914, are indeed complex. Secondly, although *Pediococcus pentosaceus*, like SMM914, has been approved for direct use as a food additive in China, further preclinical evaluations are required before conducting clinical studies examining the effects of probiotics on COPD patients. Lastly, it is important to acknowledge that the observed alterations in gut microbiota and lungs in mice may not be fully representative of the situation in individuals with COPD. This is particularly significant as the gut microbiota is influenced by various factors, including lifestyle, habits, environment, and diet.

## Materials and methods

### Oxidative stress assay of patients with COPD

A total of 42 participants, including patients diagnosed with COPD and healthy individuals, were recruited for this study. Informed consent was obtained from all participants following the approval of the ethics commission at the Affiliated Jiangning Hospital of Nanjing Medical University, China (project number: 2020-03-008-K01). COPD was diagnosed according to the Global Initiative for Chronic Obstructive Lung Disease (GOLD) criteria: patients with post-bronchodilator FEV1/FVC <7 0%, and < 80% predicted FEV1. Patients were excluded if they had been diagnosed with bronchial asthma, bronchiectasis, pulmonary fibrosis, lung tumors, and tuberculosis. The healthy individuals were recruited from the hospital’s health checkup center. Prior to any treatment, blood samples were collected, and serum was isolated through gradient centrifugation. The SOD and CAT assays were conducted following the protocol that addressed in the “Enzyme activity test”.

### Animal experiments

Female specific-pathogen-free (SPF) ICR mice (age 8 weeks) were purchased from the Jiangsu Laboratory Animal Center (Nanjing, China). All mice were housed in the SPF environment [following a 12-hour light/dark cycle (7:00 am-7:00 pm)], with 1 week acclimatization period. Mice were fed a normal chow diet (Meidicience) and drinking water during the entire experiment. Before modeling, mice were pretreated with *Pedioccus pentosaceus* SMM914 (1×10^9^ CFU/mouse every two days) and L.p (1 × 10^9^ CFU per mouse) for 2 weeks by oral gavage. During the period of modeling, continuously given probiotics until mice were sacrificed. All animal experiments were performed according to the National Institutes of Health Guide for the Care and Use of Laboratory Animals under the supervision of the Animal Ethics Committee of Nanjing Medical University (2304010). The authors further confirm that all experimental animals utilized in this study were ethically approved by the Animal Ethics Committee of Nanjing Medical University and that the study adheres to the ARRIVE guidelines.

### Model of CS-induced COPD

Mice were exposed to cigarette smoke twice a day (six cigarettes per day) for 14 weeks, with body weights being monitored weekly.

### Oral antibiotic treatment

To deplete the gut microbiota, mice were given antibiotics for two weeks, which comprised of deionized water supplemented with ampicillin (0.5 g/L, Aladdin), neomycin (0.5 g/L, Aladdin), metronidazole (0.5 g/L, Sigma), and vancomycin (0.25 g/L; Sigma), before probiotic treatment.

### Ozone-induced COPD model

Mice were exposed to ozone produced by an Ozone Cabinet (WF-F, Wuhan, China) mixed with air for 3 h at a concentration of 2.5 parts per million (ppm) in a sealed Perspex container, twice a week for 6 weeks. Ozone concentration was continuously monitored with the ozone probe. During the corresponding periods, the control groups were exposed to normal air only, twice a week for 6 weeks as well. After the beginning of the Ozone-induced model, mice were provided bronchiectants and hormone drugs (5 μg of ipratropium bromide (Boehringer Ingelheim) and 10 μg of budesonide (Pulmicort)) five times a week by nebulizer (Cofoe, China), until the mice were sacrificed.

### Pulmonary function test

Pulmonary function was accessed by a whole-body plethysmograph system (WBP-8 M, TOW, China). Following the instruction, Tidal volume, breath frequency, inspiration time (Ti), expiration time (Te), minute volume (MV), midexpiratory tidal flow (EF_50_), and Penh were determined by the software (ResMass 1.4.2.8, TOW, China). An average breathing frequency of 230 breaths/min was imposed on the mice.

### Histology and histological scores

To confirm the phenotypes of COPD, freshly harvested lung tissues were fixed in 4% paraformaldehyde overnight before being dehydrated and embedded in paraffin. Paraffin-embedded lung tissues were sectioned at a thickness of 3 μm and stained with hematoxylin and eosin (H&E). Then, they were observed and evaluated by the pathologist under an optical microscope (Olympus). Histological scoring was performed in a blinded manner according to the Internationally Harmonized Nomenclature and Diagnostic (INHAND) Proposal, with severity scores ranging from 0 (no findings) to 4 (severe changes). All the pictures were quantitative analyzed by ImageJ software.

### Serum biochemical test

0.5 mL of the whole blood from each mouse was gathered via a retro-orbital blood collection method using a 10 mm diameter glass capillary. These blood samples were left to stand for 2 h at room temperature before being centrifuged at 4°C for 10 min at 3000 rpm to separate the serum. IL-6, IL-8, IL-10, IFN-γand TNF-α were measured using a fully automated biochemical analyzer (Rayto Chemray 420, China).

### Bacterial labeling

Bacteria were inoculated overnight and transferred to fresh MRS medium at a 2% ratio. When the bacteria had grown to logarithmic phase, CY5.5-d-lys dissolved in the solution containing 0.1% corn oil and 0.9% dimethyl sulfoxide (DMSO) was added at the final concentration of 50 μg/mL and incubated for 6 h at 37°C with shaking at 200 rpm, then the bacteria were washed 3 times with PBS and resuspended in PBS [optical density at 600 nm (OD_600_) = 2.0] for the following analyses.

### In vivo tracing of fluorescence-labeled SMM914

An *in vivo* imaging system (VISQUE Invivo Smart-LF, Vieworks, Korea) and the matched software system Clevue was used for *in vivo* NIR fluorescence imaging of the colonization of SMM914 in the body. 15 ICR mice were orally administrated with 1 × 10^9^ CFU of CY5.5-d-lys labeled SMM914 per mouse. At 15 min, 45 min, and 4 h, near-infrared fluorescence imaging was conducted. Analysis software was used to measure the signal values of gut sites.

### Enzyme activity test

Lung tissue samples were homogenized in saline, followed by centrifugation (2500 × g, 4°C, 10 min) to obtain the supernatant (*n* = 7). Malondialdehyde (MDA), catalase (CAT), and superoxide dismutase (SOD) in lung were determined with commercially available colorimetric diagnostic kits (Nanjing Jiancheng Bioengineering Institute, China) following the manufacturer’s instructions.

### DDC enzyme activity assay

The enzymatic conversion of 5-hydroxy-L-tryptophan to serotonin, mediated by DDC, was investigated. A 50 μl sample, consisting of 20 μl serum (separately obtained from normal or ozone-induced COPD mice), 1 × 10^9^ CFU SMM914, and 2 μM DDC protein diluted in 0.01 M PBS at pH 6.8, was pre-incubated with 50 μl of pyridoxal-5-phosphate (0.5 mM, Aladdin, G2305812) in 300 μl of PBS. Subsequently, 100 μl of 5-hydroxy-L-tryptophan (2 mM, Aladdin, E23101122) was added, and the reaction mixture was incubated at 37°C for 1, 3, 6, and 12 hours. Following the incubation, the reaction solution was subjected to ultrafiltration using 3 K Centrifugal Filters (Merck Millipore, USA) to eliminate enzymes and other macromolecules. The reaction was terminated by heating the solution at 60°C for 15 minutes. The production of serotonin was quantified using a fully automated biochemical analyzer (Rayto Chemray 420, China).

### 16S rRNA gene amplicon sequencing

The hypervariable region V3-V4 of the bacterial 16S rRNA gene was amplified with primer pairs: 338F (5’-ACTCCTACGGGAGGCAGCAG-3’) and 806 R(5’-GGACTACHVGGGTWTCTAAT-3’), according to the standard protocols (Bioprofile, China). The raw 16S rRNA gene sequencing reads were demultiplexed, quality-filtered, and merged by QIIME Dada2 pipeline (version 1.26) with the following criteria: (i)forward reads and reverse reads are truncated at points where the average quality score of < 20, and the truncated reads shorter than 50 bp were discarded, reads containing ambiguous characters were also discarded; (ii)forward and reverse reads overlap by at least 12 bases were assembled, and chimeric sequences were identified and removed. The taxonomy of each ASV representative sequence was analyzed by QIIME feature-classifier classify-sklearn against the 16S rRNA database (Silva v138) using Default parameters.

### Metabolomics analysis

Each 50 mg lung tissue sample was added to 200 μL pre-cooled 100% methanol solution, crushed by tissue grinder, and then add 400 μL 100% methanol solution, ultrasonic mixed, centrifuged supernatant was collected, and dried by vacuum centrifugation. Mass spectrometry analysis was carried out by adding 100 μL methanol-water solution (methanol: water = 1:1, v/v) and centrifugation to analyze the supernatant. Total samples were analyzed using a UPLC-ESI-Q-Orbitrap-MS system (UHPLC, Shimadzu Nexera X2 LC-30AD, Shimadzu, Japan) coupled with Q-Exactive Plus (Thermo Scientific, San Jose, USA) according to the manufacturer’s instructions (Shanghai bioprofile). For liquid chromatography (LC) separation, samples were analyzed using an ACQUITY UPLC®HSS T3 column (2.1 × 100 mm, 1.8 μm) (Waters, Milford, MA, USA). In the sample queue, a QC sample was set up in 5 experimental samples at intervals. It was used to monitor and evaluate the stability of the system and the reliability of the experimental data. The electrospray ionization (ESI) with positive mode and the negative mode was applied for MS data acquisition separately, and analysis by QE Plus mass spectrometer (Thermo Scientific). The raw sequence data were processed using MS-DIAL for peak alignment, retention time correction, and peak area extraction. The raw MS data were processed using MS-DIAL for peak alignment, retention time correction, and peak area extraction. The metabolites were identified by accuracy mass (mass tolerance < 10 ppm) and MS/MS data (mass tolerance < 0.02 Da) which were matched with HMDB, mass bank, and other public databases. In the extracted-ion features, only the variables having more than 50% of the nonzero measurement values in at least one group were kept. Data were mean-centered using Pareto scaling. Models were built on principal component analysis (PCA), and partial least-square discriminant analysis (OPLS-DA). In the mass spectrometry comparison between SMM914 and the control, we used MRS medium as the control.

### Transcriptome data processing and analysis

Total RNA was processed using the Oligo (dT) to enrich and selected the fragments with 300bp after ion disruption. cDNA quantity and labeling efficiency were checked using Agilent 2100 Bioanalyzer and subjected to paired-end (PE) sequencing based on the Illumina HiSeq platform. Differentially expressed genes (DEGs) were identified using DEseq2 based on the following criteria: P-adjust <0.05 and |log2 Fold Change|> 1. GSEA was used to identify significantly different genes in the two groups. For the enrichment analysis, Kyoto Encyclopedia of Genes and Genomes (KEGG) pathways were analyzed.

### Immunohistochemical staining

For IHC, 5 μm lung sections underwent deparaffinization and hydration before antigen retrieval in 10 mM sodium citrate buffer at 98°C for 16 minutes. Endogenous peroxidase activity was quenched with 10% H_2_O_2_ for 20 minutes, and nonspecific antigens were blocked with serum for 30 minutes at room temperature. Subsequently, sections were incubated overnight at 4°C with primary antibodies against TLR4 (sc-293072, Santa Cruz Biotechnology) and Hspa1a (sc-66048, Santa Cruz Biotechnology). After thorough washing in PBS buffer, sections were exposed to HRP-coupled secondary antibodies for 50 minutes. Following this, the developing solution was applied, and incubation occurred in the dark at room temperature for 10 minutes. Control experiments involved omitting the primary antibodies and substituting them with nonimmune rabbit or mouse IgG. Immunohistochemical staining was observed using a microscope and quantitatively analyzed using ImageJ software.

### Immunofluorescence staining of pulmonary macrophage

For immunofluorescence staining, lung tissues were fixed with 4% paraformaldehyde overnight at 4°C and subsequently embedded in optimal cutting temperature compound (OCT). Nonspecific antigens were blocked with serum for 30 minutes at room temperature. To identify M1 and M2 macrophages in the lung tissues, anti-CD86 (GB13585, Servicebio) and anti-CD163 (GB112634, Servicebio) antibodies were used at a dilution of 1:50. Additionally, F4/80 (GB113373, Servicebio) was employed as a pan-macrophage marker at a concentration of 0.5 μg/mL. Nuclei were counterstained with DAPI (ab104139, Abcam). The obtained images were captured and analyzed using an inverted confocal laser scanning microscope (LSM 710, Carl Zeiss, Germany). Quantitative analysis was performed using ImageJ software.

### Statistical analysis

Results represent means ± SEM. Data sets involving more than two groups are assessed by one-way analysis of variance, followed by a non-parametric Kruskal-Wallis test with Newman-Keuls multiple comparisons. Statistical analyses were performed with Prism GraphPad software 9.0 (*p < .05; **p < .01; ***p < .001). *p*-value ˂ .05 was considered statistically significant. To improve sample classification and to establish reliable discriminant models, OPLS-DA was subsequently applied to distinguish beef samples with different contents and to highlight the most significant relevant features of the separation information. The variable importance in the projection (VIP) value for the model was calculated to indicate their contribution to the classification. Variables with VIP value > 1.0 were considered significantly different. The metabolites were identified using the Mouse Metabolome Database (MMDB; https://www.mmdb.ca) based on accurate mass and mass spectrometric fragmentation patterns.

## Supplementary Material

Supplementary meterials clean.docx

## Data Availability

RNA-seq data have been submitted to the National Genomics Data Center database platform (https://ngdc.cncb.ac.cn). They are publicly available under accession number PRJCA017484. SOD and CAT data from NCBI-SRA accession number PRJNA853498, representing RNA-seq data from 6 health and 6 patients with acute exacerbations of COPD, were used.

## Data and materials availability

All data associated with this study are present in the paper or the Supplementary Materials as well as from the corresponding author upon request.
