# Supplementary material for: Modulation of chronic obstructive pulmonary disease progression by antioxidant metabolites from Pediococcus pentosaceus: enhancing gut probiotics abundance and the tryptophan-melatonin pathway
Source: Gut Microbes. 2024 Mar 6;16(1):2320283. doi: 10.1080/19490976.2024.2320283 (PMC10936690; doi:10.1080/19490976.2024.2320283)
Supplement: Supplementary meterials clean.docx [file KGMI_A_2320283_SM5129.docx]

**Supplement Materials**

For the Manuscript titled “**Modulation of chronic obstructive pulmonary disease progression by antioxidant metabolites from *Pediococcus pentosaceus*: enhancing gut probiotics abundance and the tryptophan-melatonin pathway**” by Yunlei Zhang, *et al*.

1. **Supplementary Methods**
   1. **Supplementary Fig. 1**


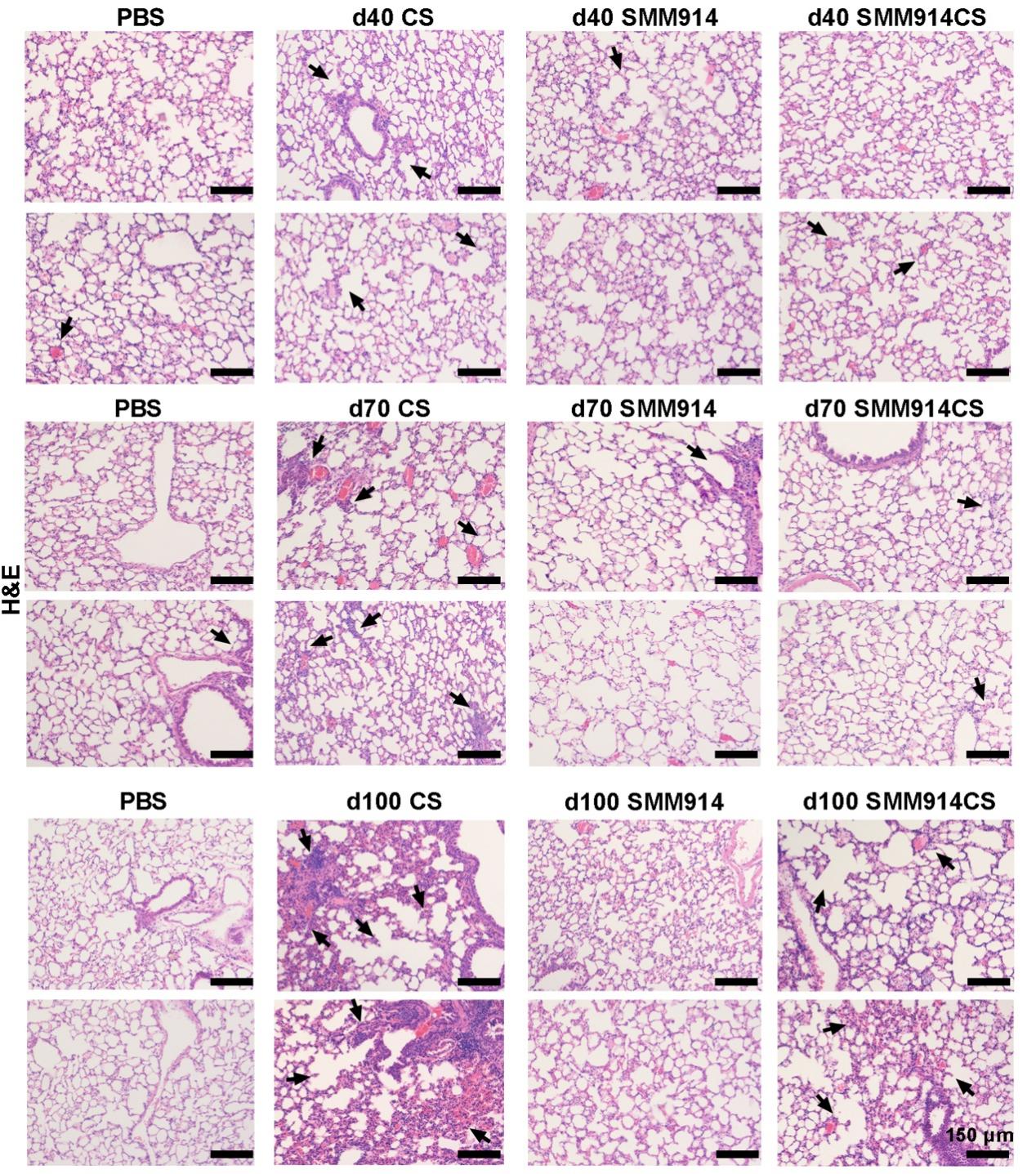


**Supplementary Fig. 1 Additional histopathology images of lungs in CS-induced mice**. The black arrows indicate damaged areas, characterized with denatured and collapsed epithelial cells, thickened alveolar septa, alveolar damage, and activated inflammatory cell infiltration. N = 3.

- 1. **Supplementary Fig. 2**

**
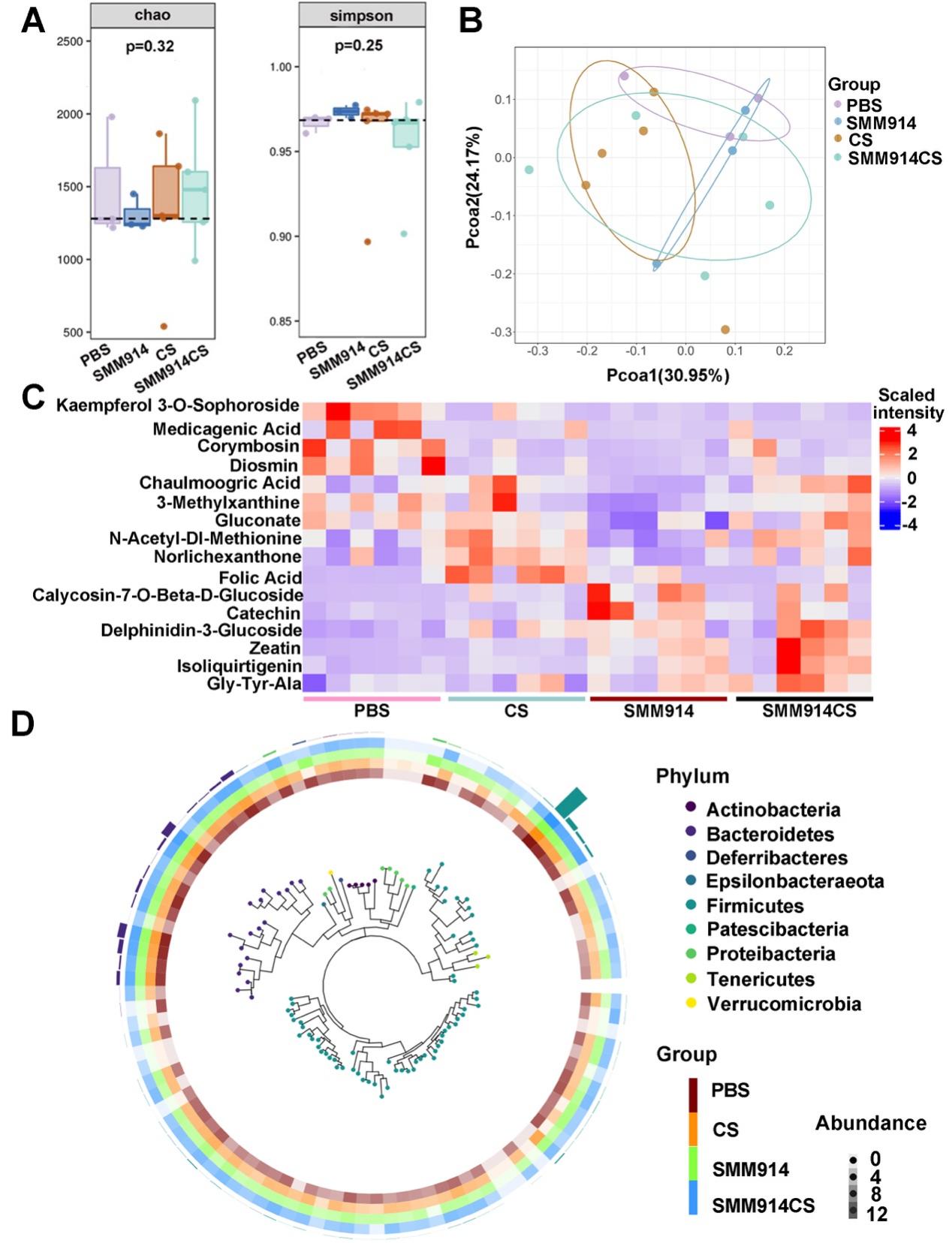
**

**Supplementary Fig. 2** **Oral administration of SMM914 altered intestinal metabolites and flora structure in CS-induced mice.** (A) 16S rRNA sequencing analysis of stool samples at day 100 after CS exposure described in Figure 1a. The graph depicts Chao and Simpson diversity index of grouped data. N = 5 - 6 mice per group. (B) Beta diversity of microbiota in four groups. (C) Heatmap of small intestinal metabolites in four group mice. N = 6. (D) Evolutionary and taxonomic relationships of four groups of mice colonies. N = 5 – 6.

- 1. **Supplementary Fig. 3**


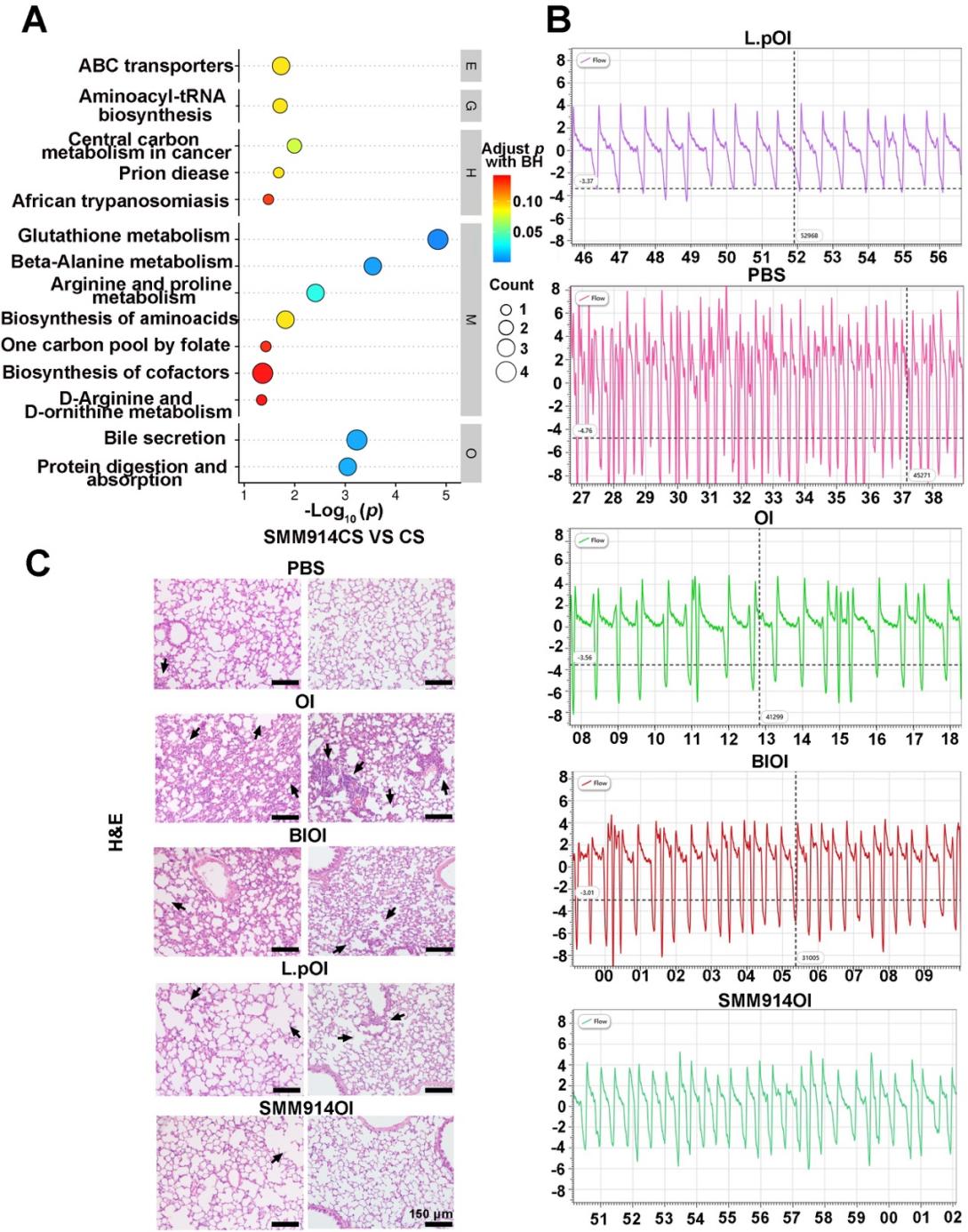


**Supplementary Fig. 3 Oral administration of SMM914 changes intestinal metabolism in CS mice and protects lung function in OI mice.** (A) Bubble plot of metabolic pathway analysis for the intestines in SMM914CS and CS groups. The color depth and bubble size indicate ln (p) values and the impact of the pathway. (B) Additional histopathology images of lungs in Ozone-induced mice. The black arrows indicate damaged areas, characterized with denatured and collapsed epithelial cells, thickened alveolar septa, alveolar damage, and activated inflammatory cell infiltration. N = 3. (C) Comparison of the respiratory curves of five groups of mice. N = 3 - 5.

**1.4．Supplementary Fig. 4**

**
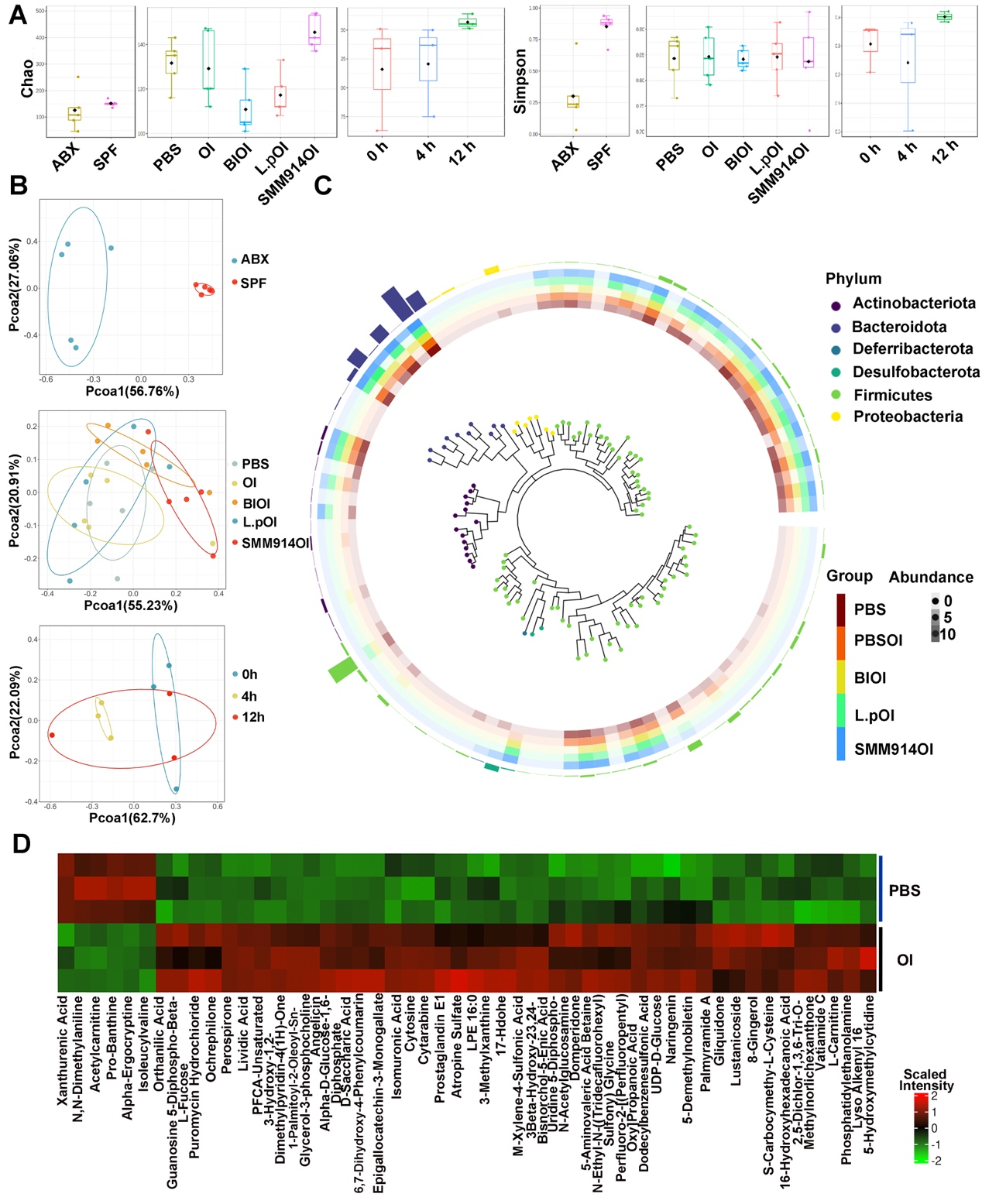
**

**Supplementary Fig. 4 Changes in the structure of the OI mice flora and alterations in metabolites.** (A) 16S rRNA sequencing analysis of stool samples at day 40 after OI exposure described in Figure 3a Graph depicts Chao and Simpson diversity index of grouped data. N = 3 - 5 mice per group. (B) Beta diversity of microbiota in five groups. N= 3 - 5. (C) Evolutionary and taxonomic relationships of five groups of mice colonies. N = 3 - 5. (D) Heatmap of lung metabolites in PBS and OI group. N = 3 – 5.

**1.5. Supplementary Fig. 5**


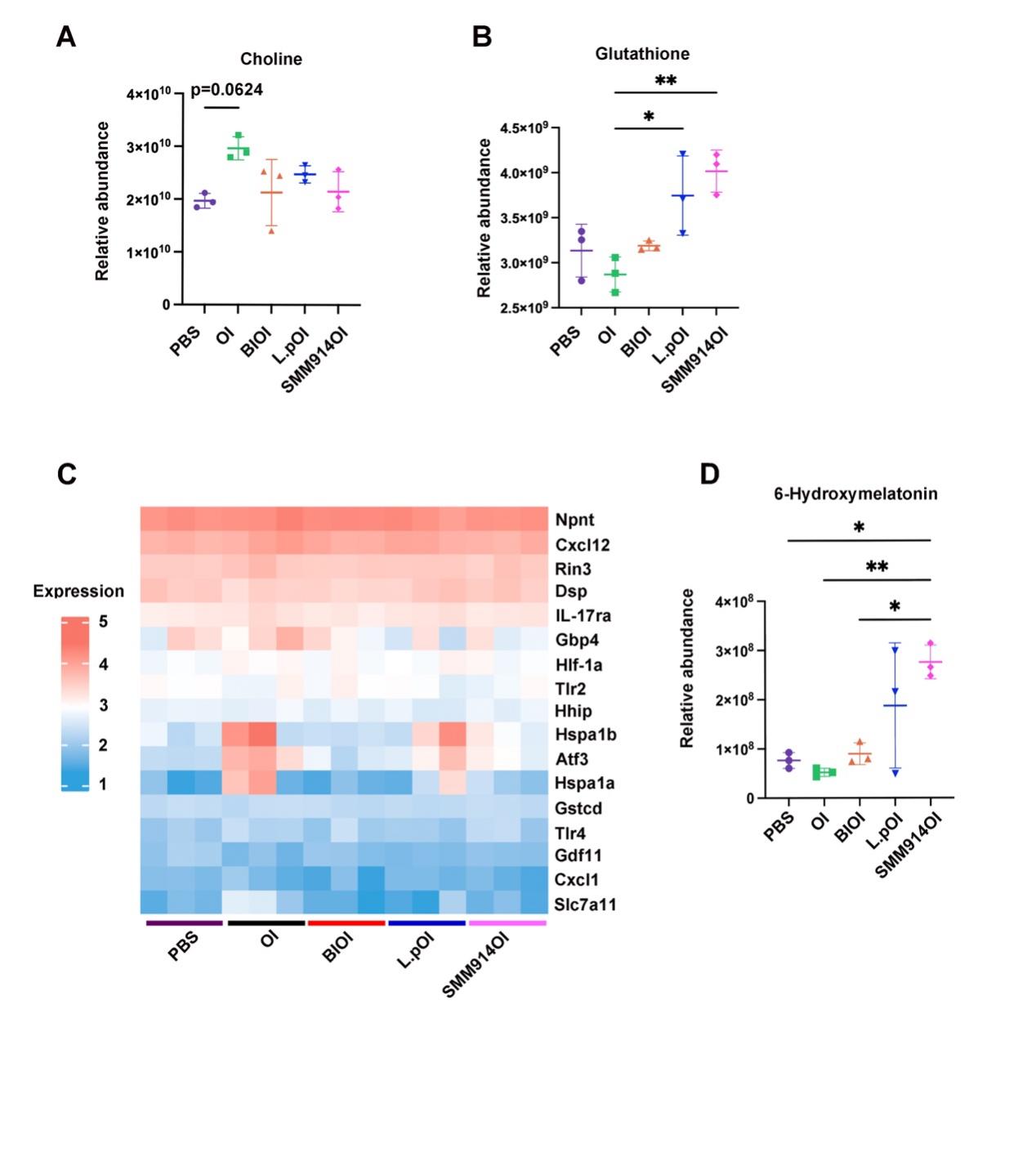


**Supplementary Fig. 5 SMM914 regulates COPD-related genes and increases antioxidant production.** (A) Lung choline concentrations in five groups. N = 3. (B) Lung glutathione concentrations in five groups. N = 3. (C) Significantly differentially expressed genes gene heatmap altered by ozone exposure, BI, L.p, and SMM914 treatment in comparison with PBS group (C) Lung 6-hydroxymelatonin concentrations in five groups. N = 3.

**1.6．Supplementary Fig. 6**

**
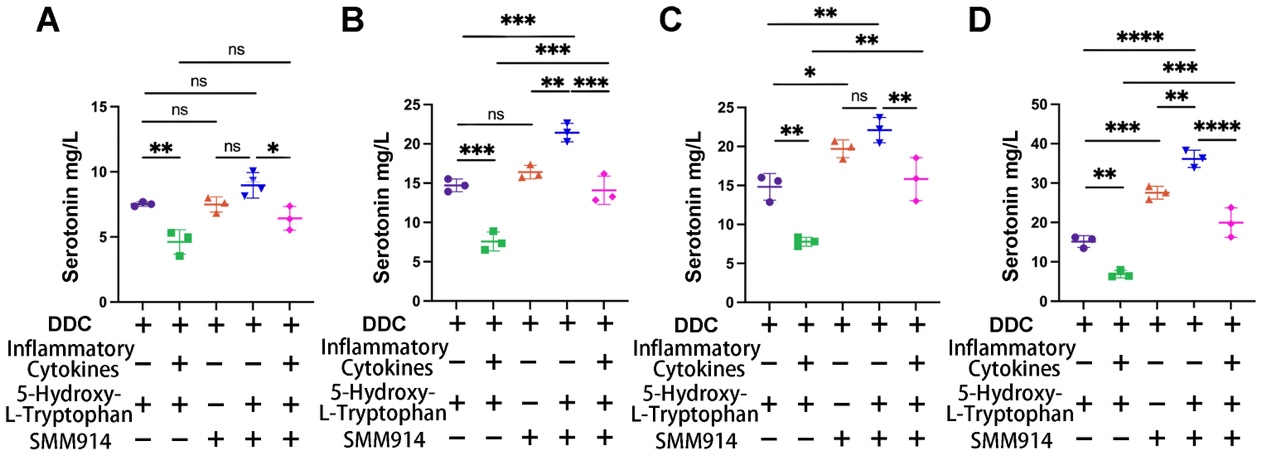
**

**Supplementary Fig. 6 The production of serotonin at four different time points.** (A) The production of serotonin after 1 hour of reaction. (B) The production of serotonin after 3 hours of reaction. (C) The production of serotonin after 6 hours of reaction. (D) The production of serotonin after 12 hours of reaction. Inflammatory cytokines refer to the COPD mouse serum in this group, while non-inflammatory cytokines indicate the normal mouse serum.

**1.7. Supplementary Fig. 7**

**
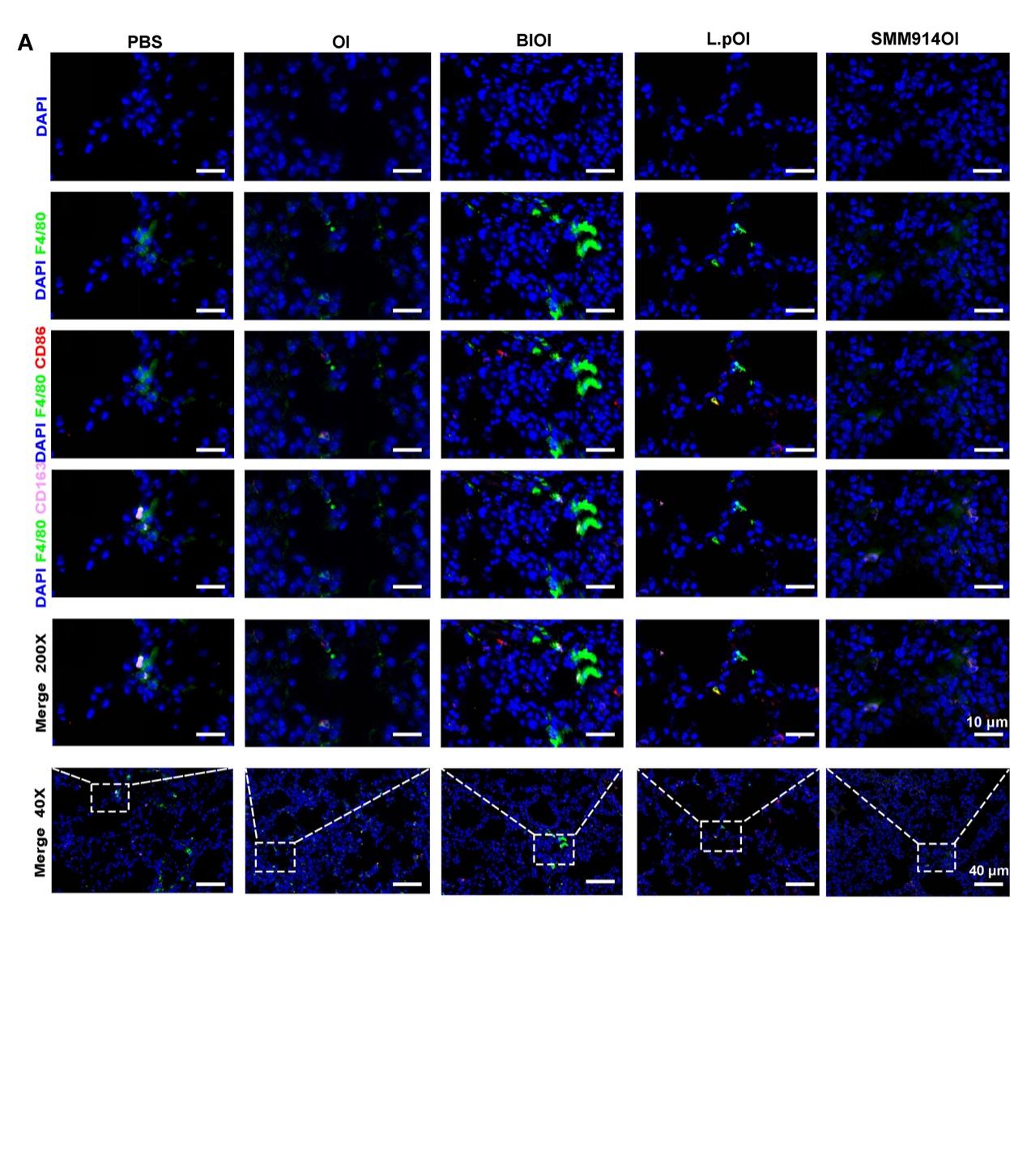
**

**Supplementary Fig. 7 SMM914 attenuates M1 macrophage polarization in the lung.** (A) Additional histopathology images of lungs in Ozone-induced mice.

**1.8. Supplementary Fig. 8**


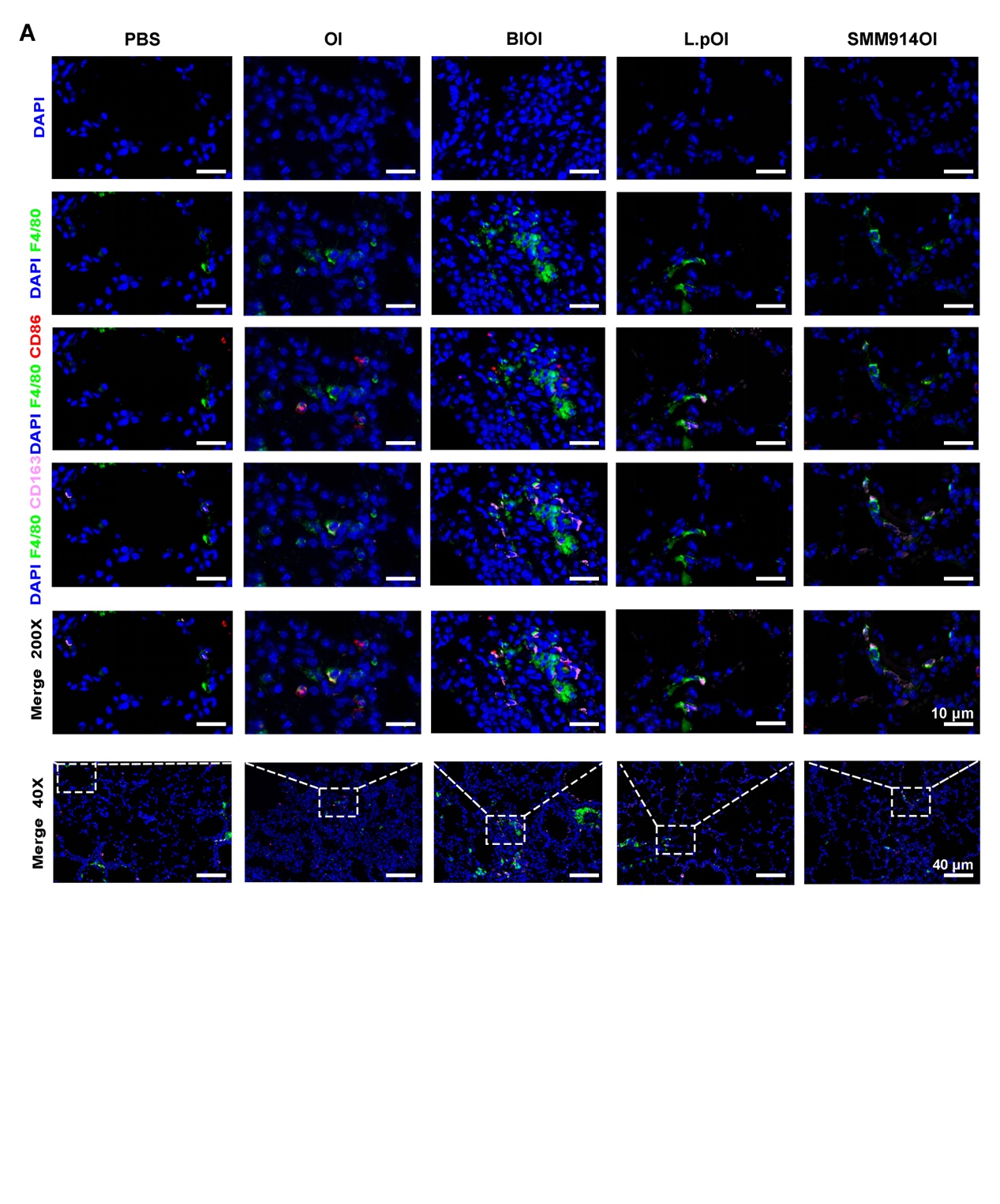


**Supplementary Fig. 8 SMM914 attenuates M1 macrophage polarization in the lung.** (A) Additional histopathology images of lungs in Ozone-induced mice.

**1.9. Supplementary Fig. 9**


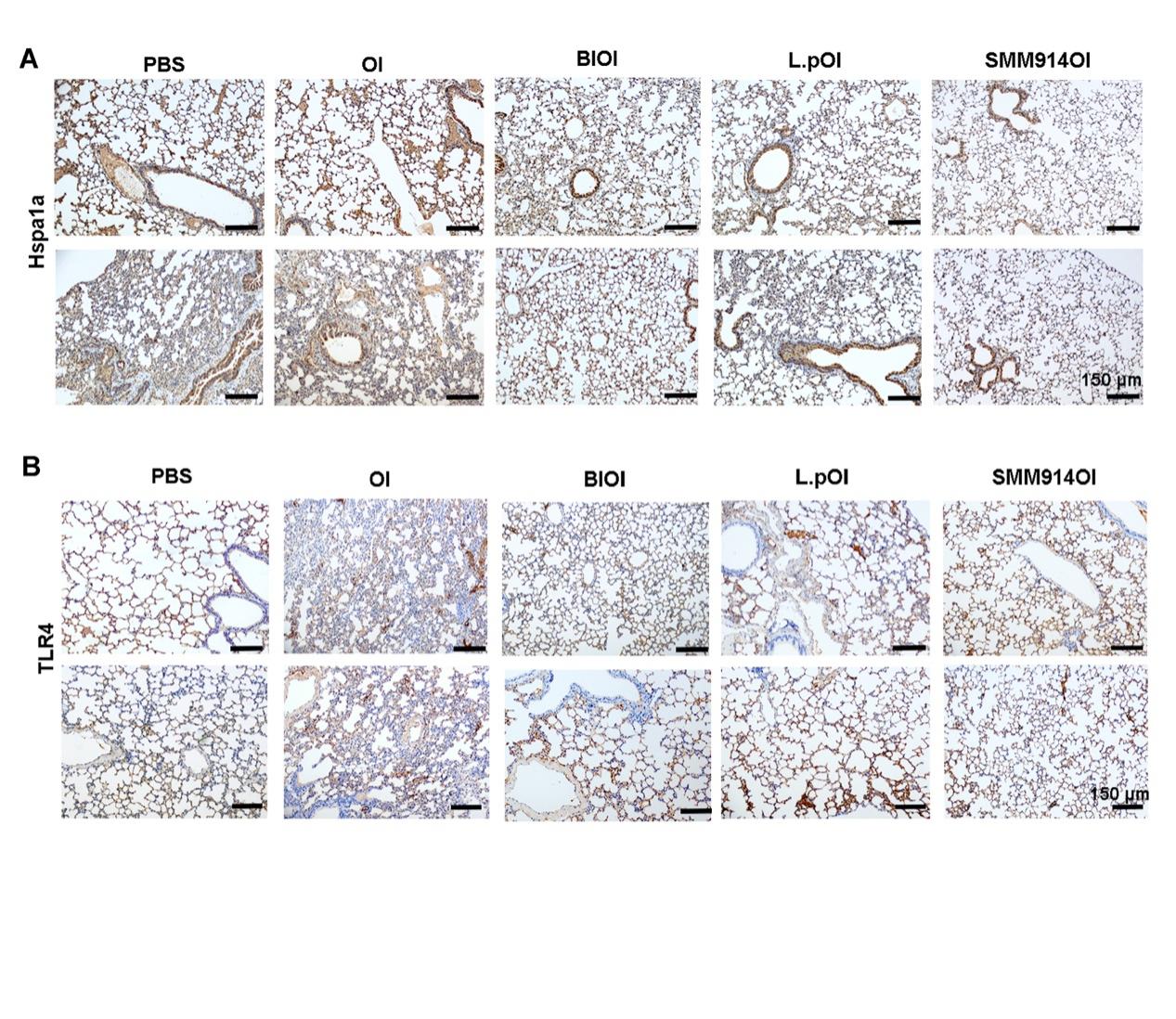


**Supplementary Fig. 9 SMM914 attenuates M1 macrophage polarization in the lung.** (A) Additional immunohistochemical staining of Hspa1a and TLR4 in lung sections in five Ozone-induced mice groups from Figure 3a.

1. **Supplementary Table**

**2.1. Supplementary Table 1. Significantly different metabolites in the four (PBS, CS, SMM914, SMM914CS) group**

| **Significantly different metabolites** | **Numbers** |
| --- | --- |
| Creatine | 1 |
| Sn-Glycero-3-Phosphocholine | 4 |
| Acetylcarnitine | 6 |
| Proline | 7 |
| Isopalmitic Acid | 7 |
| LPC 18:2 | 4 |
| Cytarabine | 1 |
| Hecogenin | 7 |
| O-Propanoylcarnitine | 5 |
| 3-Methylxanthine | 4 |
| N-Methylglutamic Acid | 4 |
| **Significantly different metabolites** | **Numbers** |
| L-Kynurenine | 8 |
| 3-Indoxyl Sulfate | 8 |
| Dichloroacetic Acid | 5 |
| D-Glyceric Acid | 4 |
| Deflazacort | 7 |
| Catechin | 4 |
| Heteratisine | 7 |
| Tobramycin | 5 |
| 5-Demethylnobiletin | 7 |
| 4-Pregnen-20Alpha-Ol-3-One | 8 |
| Fenoldopam | 4 |
| S-Adenosyl-L-methionine | 4 |
| Glycohyocholic Acid | 7 |
| Heliocurassavicine N-Oxyde | 8 |
| Solasodiene | 7 |
| Osajin | 8 |
| Fludioxonil | 4 |
| Griseofulvic Acid | 4 |
| Norethindrone | 7 |
| 10-Hydroxyusambarine | 5 |
| Riboflavin | 1 |
| Guanosine 5'-Diphospho-Beta-L-Fucose | 6 |
| Pyrrolnitrin | 5 |
| Acetaminophen Glucuronide | 4 |
| Ononin | 1 |
| Riboflavin-5'-Monophosphate | 3 |
| Tetrahydroalstonine | 1 |
| Calycosin-7-O-Beta-D-Glucoside | 8 |
| Gamma-Glutaylmethionine | 4 |
| Malvidin | 4 |
| 8-Gingerol | 5 |
| Polanrazine B | 8 |
| PFSA-H | 7 |
| Korseveriline | 7 |
| Naringenin-7-O-Beta-D-Glucoside | 3 |
| DDAO | 7 |
| Mundulone | 1 |
| Chlorothalonil-4-Hydroxy | 8 |
| Karakin | 6 |
| Benoxinate Hydrochloride | 1 |
| Karacoline | 1 |
| **Significantly different metabolites** | **Numbers** |
| Kojic Acid | 7 |
| 5-O-Methyllicoricidin | 4 |
| Methyl-Mappain | 7 |
| Senegenin | 6 |
| Parfumine | 1 |
| Vincamine | 1 |
| Guanosine 5'-Diphosphate-D-Mannose | 4 |
| Bullatine B | 8 |
| Lotaustralin | 5 |
| Ramipril | 7 |
| Pancuronium | 7 |
| Pyrrhoxanthinol | 1 |
| Meclomen | 1 |
| Trans-Zeatin-Riboside-O-Glucoside | 3 |
| Desferrioxamine X1 | 2 |
| 7-Deacetoxy-7-Oxokhivorin | 6 |
| Neoandrographolide | 1 |
| Epothilon E | 6 |
| Hydrocortisonacetate | 8 |
| N-Dodecanoyl-S-Homoserine Lactone | 1 |
| Sodium Glycocholate Hydrate | 1 |
| Fenofibric Acid | 3 |
| Phenylethylamide | 2 |
| Liriopesides B | 3 |
| 3-Pentadecylbenzene-1,2-Diol | 7 |
| Imbricaric Acid | 8 |
| Cimiracemoside D | 3 |
| N8-Acetylspermidine | 1 |

**2.2. Supplementary Table 2. Metabolomics analysis of the group (SMM914 and control)**

| **MetaboName** | | **SMM914-1** | | **SMM914-2** | | **SMM914-3** | | **CONTROL-1** | | **CONTROL-2** | | **CONTROL-3** | |  |  |  |
| --- | --- | --- | --- | --- | --- | --- | --- | --- | --- | --- | --- | --- | --- | --- | --- | --- |
| Choline | | 37845440437 | | 35615786146 | | 35287516110 | | 13354653 | | 4732059.01 | | 1387176.24 | |  |  |  |
| Acetylcholine | | 13724840423 | | 14380337038 | | 14177207251 | | 8802278.19 | | 9416850.12 | | 9149021.33 | |  |  |  |
| 1-Hydroxy-2-Naphthoic Acid | | 14943648173 | | 12260795347 | | 11762943549 | | 9593766.69 | | 8676943.8 | | 8074388.99 | |  |  |  |
| Phenylacetaldehyde | | 1738559529 | | 1784417512 | | 1869757770 | | 2699322.36 | | 3088556.97 | | 1383146.28 | |  |  |  |
| Gedunol | | 111653188.9 | | 104573459.6 | | 106942885.7 | | 143472.11 | | 301402.93 | | 151174.83 | |  |  |  |
| S-Adenosyl-L-Methionine | | 229280037.6 | | 221882927.5 | | 212467321.9 | | 923556.01 | | 84839.89 | | 227734.53 | |  |  |  |
| Tyramine | | 1197831709 | | 1232388212 | | 1261862971 | | 3720324.99 | | 2475988.71 | | 2483666.11 | |  |  |  |
| L-Beta-Homothreonine | | 1202070499 | | 915835022.5 | | 902426186 | | 5159226.99 | | 1580039.22 | | 2830120.9 | |  |  |  |
| Lorazepam | | 57374336.37 | | 41541365.7 | | 40533295.73 | | 206840.97 | | 0 | | 335604.09 | |  |  |  |
| Dihydrogedunin | | 334638689 | | 238167727.1 | | 254433995.4 | | 1608939.59 | | 835187.5 | | 869711.01 | |  |  |  |
| Cryptochlorophaeic Acid | | 39451711.47 | | 27710834.25 | | 26392594.16 | | 258122.04 | | 190037.58 | | 0 | |  |  |  |
| 2,6-Dimethoxyquinone | | 9069198.25 | | 7745139.71 | | 8173492.48 | | 98339.22 | | 2816.65 | | 33429.5 | |  |  |  |
| Thiolutin | | 47765013.27 | | 39849977.99 | | 38281900.24 | | 259722.91 | | 256297.68 | | 259341.18 | |  |  |  |
| Glyceraldehyde | | 4.08088E+11 | | 7.46313E+11 | | 7.9743E+11 | | 11389316988 | | 9294566430 | | 3880785956 | |  |  |  |
| Fludioxonil | | 1658733535 | | 1326506606 | | 1431843777 | | 22947737.16 | | 25350949.55 | | 27443146.24 | |  |  |  |
| (S,R)-Nosapine | | 20597246.97 | | 14435016.43 | | 15959255.17 | | 478050.24 | | 199517.1 | | 219949.27 | |  |  |  |
| Roccellic Acid | | 784735867.9 | | 563968357 | | 599408660 | | 11201751.44 | | 12373974.64 | | 12747151.78 | |  |  |  |
| 4-(beta-Acetylaminoethyl)imidazole | | 1063781688 | | 1530272917 | | 1512983644 | | 84566835.73 | | 2333184.07 | | 1895378.81 | |  |  |  |
| Neostigmine Methyl Sulfate | | 41410919.91 | | 44556376.68 | | 44621385.15 | | 705979.36 | | 1052560.27 | | 1460554.96 | |  |  |  |
|  | |  | |  | |  | |  | |  | |  | |  |  |  |
| **MetaboName** | | **SMM914-1** | | **SMM914-2** | | **SMM914-3** | | **CONTROL-1** | | **CONTROL-2** | | **CONTROL-3** | |  |  |  |
| Glucose 6-phosphate | | 76087723.1 | | 59086026.26 | | 73183521.64 | | 2226371.94 | | 1709194.01 | | 2014257.86 | |  |  |  |
| Ectoine | | 558488516 | | 477605489.7 | | 603751392.2 | | 35784782.32 | | 7786313.99 | | 6197288.17 | |  |  |  |
| Alpha-Guaiaconic Acid | | 46051591.6 | | 34517386.3 | | 35752509.26 | | 939973.49 | | 934001.01 | | 1878574.61 | |  |  |  |
| Makaluvamine O | | 34566187.87 | | 37226096.31 | | 36519275.29 | | 2578861.97 | | 438089.85 | | 484006.93 | |  |  |  |
| Rhodinyl Acetate | | 34500477.51 | | 29065169.72 | | 28260256.25 | | 1181927.9 | | 1515659.21 | | 716860.16 | |  |  |  |
| O-Acetyl-L-Homoserine | | 4505051843 | | 4349229636 | | 4592018181 | | 144268709.4 | | 184095994.3 | | 176569723.1 | |  |  |  |
| Barceloneic Acid A | | 35867888.02 | | 22735552.41 | | 25185891.66 | | 1097553.55 | | 817201.9 | | 1257638.27 | |  |  |  |
| Deoxycarnitine | | 11695382.67 | | 13483007.45 | | 12720133.83 | | 655681.69 | | 382973.91 | | 409412.98 | |  |  |  |
| Histamine | | 600344531 | | 575722101.9 | | 582093392.9 | | 20021329.81 | | 27719290.37 | | 27187477.22 | |  |  |  |
| Pyroglutamylisoleucine | | 1021472794 | | 1035049850 | | 1037106047 | | 46745904.45 | | 44231667.29 | | 43468708.76 | |  |  |  |
| Isofraxidin | | 938195189.2 | | 935140486.7 | | 915218951.7 | | 95250.97 | | 274450.42 | | 127629765.6 | |  |  |  |
| Aniline | | 72830210.43 | | 76386955.79 | | 77682040.2 | | 4485487.02 | | 5276099.79 | | 1455142.51 | |  |  |  |
| Phenylhydrazine | | 1901509656 | | 1948735198 | | 1879560410 | | 107167401 | | 94507568.11 | | 105723664.3 | |  |  |  |
| Candesartan | | 29804018.12 | | 22951257.12 | | 19146127.01 | | 1155778.84 | | 1207033.52 | | 1587962.72 | |  |  |  |
| Isopentenyladenine | | 4366190514 | | 3345963638 | | 3272330270 | | 578553732.1 | | 30367940.07 | | 78351157.94 | |  |  |  |
| Simonyellin | | 590985477.8 | | 465876886 | | 428662632.3 | | 33783308.98 | | 27651619.63 | | 38218057.53 | |  |  |  |
| Pachyrrhizin | | 274151454.1 | | 229400371.6 | | 243803814.7 | | 16286919.55 | | 17022147.44 | | 17208767.8 | |  |  |  |
| Calycosin-7-O-Beta-D-Glucoside | | 127956876.9 | | 96797301.47 | | 97647378.23 | | 10025353.27 | | 7634220.51 | | 7153195.87 | |  |  |  |
| Citraconic Acid | | 3220280040 | | 2577903991 | | 2548022247 | | 235257958.3 | | 223551957.8 | | 222277795.2 | |  |  |  |
| Norberto Lopes | | 4415943130 | | 5007305259 | | 5831337759 | | 547227023.4 | | 352511157.1 | | 375616819.3 | |  |  |  |
| **MetaboName** | | **SMM914-1** | | **SMM914-2** | | **SMM914-3** | | **CONTROL-1** | | **CONTROL-2** | | **CONTROL-3** | |  |  |  |
| Retusin 7-Methyl Ether | | 19121396.16 | | 12075651.29 | | 21039518.35 | | 1823628.84 | | 1842842.39 | | 943969.8 | |  |  |  |
| 7,4'-Dimethoxy-3-Hydroxyflavone | | 9309182228 | | 5098131996 | | 5726963309 | | 72998645.29 | | 54454774.07 | | 1724806023 | |  |  |  |
| Vulpinic Acid | | 779911364.8 | | 648631243 | | 602995894.7 | | 67443585.03 | | 66890537.27 | | 58525165.05 | |  |  |  |
| Methyl 2-Benzamido-3-Phenylpropanoate | | 90490008.72 | | 70276045.43 | | 78174554.33 | | 6362319.04 | | 8851344.19 | | 7545938.91 | |  |  |  |
| 10-Hydroxydecanoate | | 72746135.03 | | 54508772.15 | | 48693310.15 | | 7238292.45 | | 6156014.11 | | 6053488.15 | |  |  |  |
| N-2-Hydroxycyclopentyladenosine | | 211125180.9 | | 162178579.2 | | 160519636.5 | | 1508985.51 | | 1320383.39 | | 58221060.17 | |  |  |  |
| 4-Methylpyrimidine | | 175988031.2 | | 171271619.7 | | 161635243.2 | | 20371244.63 | | 20085662.02 | | 21138341.67 | |  |  |  |
| L-Tryptophanamide | | 4366957714 | | 3490637324 | | 3480304057 | | 67704090.38 | | 546774172.6 | | 836553446.3 | |  |  |  |
| D-Gulonic Acid Gama-Lactone | | 6479934033 | | 5105614736 | | 5121343601 | | 1004300497 | | 579235138.9 | | 587803480.7 | |  |  |  |
| Acitretin | | 44444198.17 | | 61322956.08 | | 63805037.53 | | 8193202.7 | | 8305339.67 | | 6141203.24 | |  |  |  |
| Chloro-Ib-Meca | | 111019769 | | 125350791.6 | | 125239614.2 | | 11804312.05 | | 19694871.1 | | 18404324.77 | |  |  |  |
| Pyroglutamyl-Isoleucine | | 3036574516 | | 2258522716 | | 2285369203 | | 378022831.2 | | 373977723.1 | | 370811879 | |  |  |  |
| Methyl-Beta-Galactopyranoside | | 919410389.1 | | 766541569.9 | | 714643739.3 | | 115367670.3 | | 119692447 | | 129590671.1 | |  |  |  |
| Ornithine | | 5835641574 | | 5108134424 | | 5298490959 | | 931009611.5 | | 792113419.1 | | 824058400.6 | |  |  |  |
| Uridine 5'-Diphosphate | | 4013624.08 | | 4225309.42 | | 3272528.84 | | 553297.69 | | 221541.81 | | 1042714.05 | |  |  |  |
| M-Xylene-4-Sulfonic Acid | | 3835678288 | | 3188883880 | | 1875880198 | | 923727586.3 | | 460017681.4 | | 57738756.59 | |  |  |  |
| Xylose | | 2188714416 | | 1880080485 | | 1918251552 | | 55283728.19 | | 56366844.9 | | 868219594.7 | |  |  |  |
| Guaiacin | | 147081243.7 | | 148197952.6 | | 161360751.2 | | 32394240.36 | | 24360434.48 | | 28860216.37 | |  |  |  |
| O-Succinylhomoserine | | 559272360.4 | | 396576585.5 | | 409029291.9 | | 90468558.06 | | 89798903.69 | | 80419127.65 | |  |  |  |
| **MetaboName** | | **SMM914-1** | | **SMM914-2** | | **SMM914-3** | | **CONTROL-1** | | **CONTROL-2** | | **CONTROL-3** | |  |  |  |
| Dicoumaroyl Spermidine | | 27016183.09 | | 21801757.86 | | 26802948.6 | | 6722163.92 | | 2747316.49 | | 5560993.18 | |  |  |  |
| Tetradec-5-Ynoic Acid | | 40021836.63 | | 32371961.74 | | 31451918.38 | | 6975799.96 | | 6409589.1 | | 7264288.15 | |  |  |  |
| Norharman | | 445966932.5 | | 411759108.3 | | 423730164.7 | | 152696935.6 | | 60146800.72 | | 46818760.08 | |  |  |  |
| Aspernigrin A | | 58180663.79 | | 58364922.95 | | 58454542.21 | | 35114299.84 | | 638017.9 | | 376129.56 | |  |  |  |
| 3-Phosphonopropionic Acid | | 947199838.6 | | 725251290.4 | | 691698022.2 | | 182742709.5 | | 172205191.3 | | 156927128.9 | |  |  |  |
| Ethyl-Beta-Glucuronide | | 5080285420 | | 3980834056 | | 3132767601 | | 806432437.5 | | 1009871248 | | 833824766.4 | |  |  |  |
| L-Citrulline | | 2412851846 | | 2460559440 | | 2415175987 | | 528622185.5 | | 535742548.7 | | 528204498.7 | |  |  |  |
| 4-Isopropylaniline | | 120026655.7 | | 120763323.3 | | 117661733.5 | | 2924219.38 | | 40775956.67 | | 39747901.16 | |  |  |  |
| PFCA-Unsaturated | | 29618546.87 | | 26941480.45 | | 26109839.92 | | 7833624.95 | | 6381240.56 | | 5432158.61 | |  |  |  |
| 6-Hydroxyflavone | | 4859684022 | | 3660443497 | | 3610938199 | | 814819479.5 | | 1077325749 | | 1014079296 | |  |  |  |
| 2',4',6'-Trihydroxydihydrochalcone | | 3126766840 | | 2477527683 | | 2348141220 | | 649710885.8 | | 614148073.8 | | 642308194.5 | |  |  |  |
| Trehalose-6-Phosphate | | 11306532.1 | | 10334974.75 | | 11374917.51 | | 2773368.41 | | 2798630.15 | | 2474722.29 | |  |  |  |
| 2,4,5-Trimethoxybenzoic Acid | | 110894360.1 | | 97746376.62 | | 99348806.74 | | 25598394.67 | | 19294610.21 | | 30264628.32 | |  |  |  |
| 6,4'-Dihydroxyflavone | | 250093983.9 | | 195645548.9 | | 214066528.7 | | 59487452.83 | | 22348894.94 | | 83815891.41 | |  |  |  |
| Camalexin | | 6219994949 | | 4924688282 | | 4917364204 | | 1389080509 | | 1414555538 | | 1282293765 | |  |  |  |
| Isoleucylvaline | | 109669300.2 | | 99833804.61 | | 99057002.55 | | 41224971.05 | | 20326240.32 | | 18318631.76 | |  |  |  |
| (S)-1-Carbamoylpyrrolidine-2-Carboxylic Acid | | 694534850.9 | | 699495992.1 | | 703560810.6 | | 178556951.2 | | 189138949.2 | | 179671862.6 | |  |  |  |
| Apicidin B | | 8074574.29 | | 11010794.6 | | 7328376.7 | | 2425073.62 | | 2245314.7 | | 2327702.33 | |  |  |  |
| **MetaboName** | | **SMM914-1** | | **SMM914-2** | | **SMM914-3** | | **CONTROL-1** | | **CONTROL-2** | | **CONTROL-3** | |  |  |  |
| Alpha-Lobeline | | 632620.42 | | 328783.28 | | 751227.4 | | 241428.65 | | 133271.69 | | 93479.82 | |  |  |  |
| Hydroxymethylglutaric Acid | | 7644233830 | | 6239845365 | | 5794997704 | | 1708197672 | | 1928240565 | | 1882962826 | |  |  |  |
| Sparfloxacin | | 45394812.36 | | 39812351.88 | | 31962319.21 | | 9332462.68 | | 12075994.36 | | 11671252.41 | |  |  |  |
| 16-Hydroxypalmitate | | 58477187.57 | | 45159328.59 | | 39836641.08 | | 13361356.55 | | 14459277.19 | | 12732480.5 | |  |  |  |
| D-Gluconic Acid | | 21375906957 | | 16313182551 | | 16477227872 | | 5236442619 | | 5374528462 | | 4857325621 | |  |  |  |
| PFOH-Perfluoroalkyl Alcohol | | 219244063.3 | | 203329844.1 | | 191238525 | | 66447497.84 | | 59582890.67 | | 49321468.53 | |  |  |  |
| Sinomenine | | 80649345.94 | | 72436375.28 | | 43921092.04 | | 20098512.32 | | 20505637.15 | | 16954622.86 | |  |  |  |
| Isorhynchophylline | | 22116936.32 | | 31286035.65 | | 31893473.59 | | 6230044.06 | | 9882053.44 | | 9029298.61 | |  |  |  |
| Adenine | | 26082517557 | | 21561553355 | | 20378173494 | | 7223552995 | | 6841672403 | | 6230812762 | |  |  |  |
| PFSA-Ether | | 102290151 | | 99448160.35 | | 96704304.28 | | 34029301.17 | | 29433309.03 | | 26886893.1 | |  |  |  |
| Dihydrozeatin | | 193590657.7 | | 163678586.3 | | 147715697.2 | | 55777541.01 | | 51479069.91 | | 46491391.88 | |  |  |  |
| Pyrimidinol | | 613736732.6 | | 489148491.4 | | 472512986 | | 189834596.5 | | 173821571.4 | | 164332788.4 | |  |  |  |
| Cytosine | | 3510155621 | | 3508246639 | | 3482772038 | | 1194442448 | | 1127974125 | | 1198475491 | |  |  |  |
| Bullatine G | | 6167942.61 | | 9691304.91 | | 8406601.38 | | 2761288.51 | | 3125310.91 | | 2606633.34 | |  |  |  |
| (S)-Dihydroorotate | | 345662348.5 | | 272815359.1 | | 276737388.6 | | 110844300.8 | | 107394442.8 | | 102582491.6 | |  |  |  |
| 2-Aminobenzoic Acid | | 121362933.7 | | 98508885.88 | | 108266437.7 | | 43747258.38 | | 42189741.32 | | 32782776.01 | |  |  |  |
| Djenkolic Acid | | 551853127.4 | | 516665374.9 | | 519457322 | | 227949282.8 | | 194696763.6 | | 152793428.8 | |  |  |  |
| Isolaserpitin | | 169409384.7 | | 163378007.3 | | 177154707.2 | | 68118736.05 | | 61611637.62 | | 56281943.59 | |  |  |  |
| Amoxapine | | 12359331.95 | | 11113634.07 | | 10636029.91 | | 1530732.41 | | 5399016.05 | | 5677862.69 | |  |  |  |
| 3-Dehydroshikimate | | 2339565152 | | 2193601092 | | 2180382835 | | 946029944.1 | | 848736814.3 | | 708042406.8 | |  |  |  |
| **MetaboName** | | **SMM914-1** | | **SMM914-2** | | **SMM914-3** | | **CONTROL-1** | | **CONTROL-2** | | **CONTROL-3** | |  |  |  |
| But-3-Enylglucosinolate | | 40519051.05 | | 34209304.4 | | 29952001.03 | | 16255959.43 | | 13259288.93 | | 9670578.4 | |  |  |  |
| 2-Hydroxyisocaproic Acid | | 5992138855 | | 4844612338 | | 4892010217 | | 1920980101 | | 1965444624 | | 2270527164 | |  |  |  |
| Theaflavin-3-Gallate | | 20060902.34 | | 16957657.24 | | 21250674.84 | | 8213461.72 | | 7609920.13 | | 7439169.92 | |  |  |  |
| Cyclopenin | | 186305633.7 | | 137220057.7 | | 144164128.6 | | 65636660.92 | | 62189688.48 | | 60233111.86 | |  |  |  |
| 3-Methyladenine | | 1580619265 | | 1568554620 | | 1608898122 | | 685838508.6 | | 650774469.5 | | 613001674.2 | |  |  |  |
| Stylopeptide 1 | | 26054522.42 | | 22759345.78 | | 26992348.71 | | 12026981.66 | | 11005341.52 | | 10420632.9 | |  |  |  |
| 3-Methylthiopropylamine | | 88282208.11 | | 94238243.31 | | 94441374.33 | | 39397301.36 | | 41079485.67 | | 43602930.66 | |  |  |  |
| Allysine | | 10622257575 | | 9591253864 | | 10566360611 | | 4732393672 | | 4753448400 | | 4349031485 | |  |  |  |
| Ochrolifuanine A | | 1043042551 | | 983786433.1 | | 1079215882 | | 486274857.3 | | 476763853.1 | | 441413832.2 | |  |  |  |
| Kainic Acid | | 140013277.3 | | 157035587.9 | | 164477888.7 | | 67440534.09 | | 72585335.3 | | 71582278.52 | |  |  |  |
| Nordihydrocapsaicin | | 61602328.33 | | 60773015.49 | | 61042376.97 | | 25122647.38 | | 29286648.55 | | 31034698.73 | |  |  |  |
| D-Xylulose | | 108583748.3 | | 94241783.43 | | 97762146.8 | | 50966037.98 | | 47488760.09 | | 41777624.66 | |  |  |  |
| Mefenamic Acid | | 369183978 | | 367131616.1 | | 355336074.1 | | 164674857.9 | | 170153896.9 | | 177050024 | |  |  |  |
| 5-O-Methyllicoricidin | | 23602636.72 | | 21794047.3 | | 21838342.48 | | 14765081.99 | | 14266770.7 | | 3254442.72 | |  |  |  |
| Hydroxysebacic Acid | | 38155340.15 | | 31970784.9 | | 27803057.9 | | 19143733.64 | | 12776361.15 | | 15176229.37 | |  |  |  |
| Placodiolic Acid | | 123373121.7 | | 93245248.4 | | 99283296.51 | | 40850411.01 | | 73040929.58 | | 41155003.82 | |  |  |  |
| N-Acetyl-Dl-Serine | | 991074902.5 | | 816737029.5 | | 756993017.8 | | 398628407.3 | | 446685321.5 | | 417135116.9 | |  |  |  |
| 5-Hydroxymethyluridine | | 457992975.5 | | 338722248.2 | | 356925468.6 | | 171029898.4 | | 171176529.4 | | 231442165.7 | |  |  |  |
| Theobromine | | 81920418.53 | | 58199797 | | 64509094.61 | | 35507299.69 | | 32030860 | | 34772716.5 | |  |  |  |
| (+-)-Baclofen | | 88069544.68 | | 69477372.24 | | 63525329.68 | | 35221995.22 | | 42337833.35 | | 33395904.11 | |  |  |  |
| **MetaboName** | | **SMM914-1** | | **SMM914-2** | | **SMM914-3** | | **CONTROL-1** | | **CONTROL-2** | | **CONTROL-3** | |  |  |  |
| L-Proline | | 4180752402 | | 3365619114 | | 3337116502 | | 1822147122 | | 1925814163 | | 1764879495 | |  |  |  |
| Eicosenoic Acid | | 52421486.26 | | 42977292.85 | | 42203674.42 | | 23324748.95 | | 21451129.61 | | 25206252.19 | |  |  |  |
| Cantil | | 311348160.6 | | 304891678.8 | | 333225612.8 | | 166540796.7 | | 161357603.8 | | 155028368.6 | |  |  |  |
| L-Ornithine | | 3523360125 | | 2916852631 | | 2798940107 | | 1516694414 | | 1616650345 | | 1626781351 | |  |  |  |
| Maleic Acid | | 675131434.4 | | 470819479.3 | | 488335185.5 | | 305304118.3 | | 249380220.3 | | 293151901.4 | |  |  |  |
| Rhetsinine | | 19834168.34 | | 20473827.49 | | 19100896.52 | | 11099817.83 | | 10250090.75 | | 9939778.5 | |  |  |  |
| Glycolithocholic Acid | | 657838271.3 | | 543166682.3 | | 573644768.1 | | 341794254.2 | | 319641998.8 | | 288884305.4 | |  |  |  |
| PFCA-Unsaturated Ether | | 52661076.9 | | 39018102.89 | | 36178796.4 | | 26979848.56 | | 20744620.38 | | 22487880.95 | |  |  |  |
| Galanthamine Hydrobromide | | 39269364.79 | | 29367177.42 | | 24510633.74 | | 18064568.49 | | 17661818.18 | | 15737592.86 | |  |  |  |
| Melezitose | | 159089708 | | 157649096.2 | | 162672087.6 | | 94859227.76 | | 95674835.22 | | 90827697.25 | |  |  |  |
| R(+)-IAA | | 52077292.89 | | 41014107.03 | | 37971532.17 | | 27206699.38 | | 22868885.38 | | 27136985.75 | |  |  |  |
| Morpholine | | 2205839800 | | 2175667765 | | 2426214157 | | 1436603702 | | 1325394251 | | 1306983319 | |  |  |  |
| Ecgonine | | 75574570.86 | | 61608763.18 | | 79745476.55 | | 45549093.88 | | 42034026.19 | | 42110408.07 | |  |  |  |
| Gramine | | 78805679.21 | | 62022983.09 | | 57624708.98 | | 41412787.35 | | 41055503.73 | | 36831503.38 | |  |  |  |
| L-Kynurenine | | 50347013.1 | | 49508543.51 | | 48559787.18 | | 31686514.78 | | 28316179.14 | | 29612621.58 | |  |  |  |
| Corynoxine | | 17822411.86 | | 21546673.36 | | 21360680 | | 11995404.03 | | 13295361.3 | | 12237277.71 | |  |  |  |
| D-Ribose 5-Phosphate | | 23763291.22 | | 16903361.58 | | 18569888.75 | | 12061472.16 | | 12378607.78 | | 12637824.27 | |  |  |  |
| Veratramine | | 1168970403 | | 1053828651 | | 1072490512 | | 718318332.1 | | 685529018.5 | | 661033050.9 | |  |  |  |
| S-Sulfo-L-cysteine | | 14162410.63 | | 11204236.55 | | 10963369.2 | | 9197800.19 | | 5208866.96 | | 8374051.3 | |  |  |  |
| Spectinomycin | | 690444804 | | 517272849.4 | | 511322010.1 | | 398273367.2 | | 376292863.1 | | 304951179.3 | |  |  |  |
| **MetaboName** | | **SMM914-1** | | **SMM914-2** | | **SMM914-3** | | **CONTROL-1** | | **CONTROL-2** | | **CONTROL-3** | |  |  |  |
| Glycocholic Acid | | 77945629.75 | | 81208930.28 | | 92220533.08 | | 55330309.49 | | 48587452.05 | | 54193558.62 | |  |  |  |
| Cezomycin | | 126984223 | | 122098584.2 | | 122131315 | | 75789964.93 | | 80259210.41 | | 77965378.85 | |  |  |  |
| Furosemide | | 6298732.58 | | 4575917.46 | | 4271890.78 | | 3429077.8 | | 3367260.4 | | 2767084.37 | |  |  |  |
| Methoxycinnamic Acid | | 41823453.49 | | 39359400.19 | | 45613342.15 | | 27083803.31 | | 26496969.39 | | 26768973.27 | |  |  |  |
| Ganolactone B | | 38848774.21 | | 56025777.82 | | 53509233.24 | | 30400263.31 | | 30496104.48 | | 34350194.23 | |  |  |  |
| Roquefortine C | | 22012130.66 | | 19185168.35 | | 21752544.11 | | 12164514.26 | | 15076783.37 | | 13218242.97 | |  |  |  |
| Myricitrin | | 5044946.33 | | 6191057.81 | | 6021022.25 | | 2811489.75 | | 4050001.11 | | 4453578.53 | |  |  |  |
| D-(-)-Gulono-Gamma-Lactone | | 2666971683 | | 3389727705 | | 3809879891 | | 2196995273 | | 2117855443 | | 2199032116 | |  |  |  |
| Edpetiline | | 150275867.9 | | 116252199.6 | | 148657079.2 | | 94609701.05 | | 90795660.69 | | 90066673.21 | |  |  |  |
| N-Acetyl-D-Tryptophan | | 22542524.24 | | 22896807.32 | | 22313304.2 | | 15441357.73 | | 16173405.31 | | 13712129.49 | |  |  |  |
| Glycohyodeoxycholic Acid | | 21875133.58 | | 16527435.47 | | 20345465.04 | | 13185150.73 | | 12121942.12 | | 14015297.58 | |  |  |  |
| Adenosine Monophosphate | | 486971491.2 | | 517893067.3 | | 501175179.9 | | 341144028.6 | | 336819072.5 | | 335305713.8 | |  |  |  |
| Dihydroquercetin | | 185321287.5 | | 169277895.7 | | 178575864.5 | | 129713638.5 | | 116485096.5 | | 113003649.3 | |  |  |  |
| Guanosine 5'-Monophosphate | | 183579736.2 | | 193624863.7 | | 197794531.7 | | 129127261.8 | | 133418907.2 | | 129718283 | |  |  |  |
| Porphobilinogen | | 1070007265 | | 916695255.9 | | 909015691.9 | | 691015450.5 | | 657983086.4 | | 650180564.4 | |  |  |  |
| Inosine 5'-monophosphate | | 12178937.8 | | 12612998.62 | | 12648504.86 | | 8386058.35 | | 8871239.72 | | 8680240.81 | |  |  |  |
| Taurodeoxycholic Acid | | 91960950.61 | | 94996448.36 | | 97850845.96 | | 63811147.93 | | 71512847.85 | | 63864843.44 | |  |  |  |
| Aspartic Acid | | 2583507190 | | 2757420137 | | 2686235315 | | 1874305645 | | 1848084875 | | 1894093052 | |  |  |  |
| Salsolidine | | 313025255.5 | | 269273572.1 | | 275434712.1 | | 233871263.5 | | 194024411.1 | | 176143942.6 | |  |  |  |
| Amisulpride | | 696895905.6 | | 567637556.2 | | 540114584.5 | | 433372895.8 | | 425100404.2 | | 439580848.8 | |  |  |  |
| **MetaboName** | | | | **SMM914-1** | | **SMM914-2** | | **SMM914-3** | | **CONTROL-1** | | **CONTROL-2** | | **CONTROL-3** | |  |
| D-Tryptophan | | 108487129.8 | | 117989303.2 | | 131675342.8 | | 83283957.69 | | 89358996.97 | | 85980660.44 | |  |  |  |
| Haploside C | | 21859527.3 | | 24240691.1 | | 18061733.17 | | 16147131.12 | | 15299665.55 | | 15159442.77 | |  |  |  |
| Garcinolic Acid | | 77196132.82 | | 87442989.4 | | 85330770.4 | | 50787505.03 | | 61525282.43 | | 70713052.13 | |  |  |  |
| Demethylzeylasteral | | 106249760.2 | | 134094714.1 | | 129492239.3 | | 81069062.37 | | 90997031.97 | | 100258992.5 | |  |  |  |
| Pseudojervine | | 113476156.7 | | 106425787.3 | | 107735814.9 | | 78980953.65 | | 85893145.96 | | 77820829.58 | |  |  |  |
| Methyl Jasmonate | | 74903721.18 | | 89379058.86 | | 82888925.93 | | 63863686.35 | | 61690832.01 | | 62247050.2 | |  |  |  |
| Rutamarin | | 58354676.79 | | 59881943.68 | | 57009318.37 | | 39846943.55 | | 48538510.35 | | 44828402.52 | |  |  |  |
| Amlexanox | | 72854677.09 | | 58945360.36 | | 58722264.83 | | 45946015.12 | | 50683200.39 | | 49210837.44 | |  |  |  |
| 3-Deoxycaryoptinol | | 56198379.46 | | 55313026.27 | | 54666624.85 | | 43112750.89 | | 44211271.22 | | 40661295.15 | |  |  |  |
| Salmeterol Xinafoate | | 115347389.8 | | 139888630.6 | | 143621130.4 | | 98506392.11 | | 100112931.4 | | 111855274.8 | |  |  |  |
| L-Methionine | | 9566175361 | | 9273667885 | | 9408233169 | | 7528786935 | | 7375458508 | | 7207759107 | |  |  |  |
| Rutaecarpine | | 24241529.31 | | 24070679.86 | | 23307108.23 | | 17134824.46 | | 19630228.85 | | 19449364.11 | |  |  |  |
| 17-Alpha-Ethinylestradiol | | 107083252.6 | | 130220781.9 | | 116216874.5 | | 93441511.23 | | 97917498.42 | | 86585745.06 | |  |  |  |
| Oridonin | | 218849529.4 | | 209631808.1 | | 230657268.5 | | 180046511 | | 168145540.2 | | 175892918.6 | |  |  |  |
| Prolylproline | | 91152360.18 | | 80590629.72 | | 88883821.53 | | 70469294.75 | | 65237642.12 | | 72636449.05 | |  |  |  |
| Nepsilon,Nepsilon,Nepsilon-Trimethyllysine | | 926969438.1 | | 897411224.1 | | 937329725.5 | | 760525306.2 | | 736248830.9 | | 715459925.9 | |  |  |  |
| Costunolide | | 126475532.4 | | 125460790.5 | | 134564143.1 | | 107112999.4 | | 101419432.7 | | 104359190.1 | |  |  |  |
| Caffeine | | 1459382790 | | 1539910466 | | 1532441736 | | 1276130473 | | 1270886870 | | 1237521221 | |  |  |  |
| 2-Octyl-3(2H)-Isothiazolone | | 996833163.6 | | 1048119805 | | 1000327249 | | 821925112.3 | | 896256463.2 | | 887208873.1 | |  |  |  |
| Zeatin | | 107585503.4 | | 106923174.9 | | 97389477.95 | | 92080945.92 | | 84020875.39 | | 92131028.74 | |  |  |  |
| **MetaboName** | | **SMM914-1** | | **SMM914-2** | | **SMM914-3** | | **CONTROL-1** | | **CONTROL-2** | | **CONTROL-3** | |  |  |  |
| Valsartan | | 19075320.79 | | 20935930.35 | | 21559643.82 | | 17235502.92 | | 18129093.6 | | 18198213.56 | |  |  |  |
| 2-Methylpyrrolidine | | 10762027811 | | 10698037462 | | 10737816443 | | 9935006276 | | 9350983705 | | 9546107893 | |  |  |  |
| Nefazodone | | 78870832.04 | | 72239266.84 | | 77962670.13 | | 70919889.05 | | 68831917.43 | | 66111564.44 | |  |  |  |
| Anabasamine | | 6464624987 | | 7042855304 | | 6698364403 | | 6063827557 | | 6091822497 | | 6149878483 | |  |  |  |
| 1-(3-(Trifluoromethyl)Phenyl)Piperazine | | 117331644.9 | | 125176166.1 | | 122837922 | | 115646876.9 | | 110774117 | | 106393922.7 | |  |  |  |
| Disipal | | 438025897.3 | | 447243741.5 | | 456775745.7 | | 400501689.9 | | 413395249.2 | | 412018631.6 | |  |  |  |
| Pseudo-Anisatin | | 117302545.1 | | 109628433 | | 107475832.8 | | 102938201.9 | | 103326669.3 | | 103138316.6 | |  |  |  |
| His | | 6996896796 | | 6812058713 | | 6919809010 | | 6277320841 | | 6559171879 | | 6493191154 | |  |  |  |
| Cytidine 5'-Monophosphate | | 187042002.8 | | 191643182.3 | | 187917077.2 | | 175766726.9 | | 181741933.1 | | 177112386.2 | |  |  |  |
| Diallyl Sulfide | | 9415638708 | | 9699835332 | | 9740756079 | | 10094073009 | | 10014969649 | | 9842015790 | |  |  |  |
| Lysine | | 25829797005 | | 25570410898 | | 26382091864 | | 27854066746 | | 26783539998 | | 27279524500 | |  |  |  |
| Kukoamine A | | 42785136.82 | | 41814928.85 | | 43402945.17 | | 45829720.11 | | 45729621.2 | | 44146195.04 | |  |  |  |
| L-Pipecolate | | 6141260669 | | 6005572670 | | 6342276162 | | 6802197458 | | 6575352525 | | 6434166750 | |  |  |  |
| Cytidine 5'-Diphosphocholine | | 14925338.58 | | 14853456.01 | | 15770931.26 | | 16516359.2 | | 16302788.46 | | 16010526.66 | |  |  |  |
| Fraxin | | 399282980.1 | | 395465444.7 | | 389872721.6 | | 441359918 | | 428395618.9 | | 406293976 | |  |  |  |
| Carbazole | | 173240454.5 | | 170804385.1 | | 176921887 | | 187063857.7 | | 195145964.4 | | 180856067.3 | |  |  |  |
| Leucylproline | | 2824182925 | | 2669093995 | | 2836878738 | | 3047970163 | | 3039729856 | | 2943945378 | |  |  |  |
| Haplamine | | 197386896.5 | | 194117621.2 | | 205825746.9 | | 222649218.1 | | 226035156.6 | | 211800347.6 | |  |  |  |
| Magnoflorine Iodide | | 44901399.87 | | 45335756.78 | | 47061142.26 | | 51488196.49 | | 52309668.37 | | 48514849.08 | |  |  |  |
| **MetaboName** | | **SMM914-1** | | **SMM914-2** | | **SMM914-3** | | **CONTROL-1** | | **CONTROL-2** | | **CONTROL-3** | |  |  |  |
| Atrazine | | 1495230287 | | 1495885785 | | 1523293085 | | 1700582590 | | 1676830247 | | 1648030354 | |  |  |  |
| N-Acetyltryptophan | | 23632088.22 | | 21789633.74 | | 24368602.15 | | 26950456.6 | | 25202428.6 | | 25546776.61 | |  |  |  |
| Proline | | 12430999610 | | 13568449970 | | 13301772327 | | 14457880574 | | 14981308754 | | 14466786803 | |  |  |  |
| Levamisole | | 145919403.7 | | 146754555.6 | | 143549611.4 | | 159656236.4 | | 165642499 | | 164030665.3 | |  |  |  |
| Arteannuin B | | 201618711.9 | | 188289261.9 | | 194785214.2 | | 218431731.6 | | 227260375.1 | | 210307770 | |  |  |  |
| Gluconasturtiin | | 1042209352 | | 1027622514 | | 1043431248 | | 1145928697 | | 1219730830 | | 1144362155 | |  |  |  |
| Albendazole | | 383412162.9 | | 405069633 | | 400916338.3 | | 473554305.5 | | 445061691.3 | | 422916235.1 | |  |  |  |
| Eudesmin | | 19169692 | | 19073567.75 | | 19098197.08 | | 22749316.02 | | 21315666.99 | | 20908550.35 | |  |  |  |
| N4-Acetylsulfathiazole | | 2877035752 | | 2847675818 | | 2933674671 | | 3282958547 | | 3304374507 | | 3223704814 | |  |  |  |
| Gossypol | | 12849980.71 | | 12464509.52 | | 13130930.64 | | 14950691.13 | | 15081983.89 | | 14257119.5 | |  |  |  |
| Corynanthin | | 89814177.41 | | 83134804.49 | | 88602304.01 | | 105866079.9 | | 101627614.5 | | 94113027.87 | |  |  |  |
| Rescinnamine | | 12909582.47 | | 12329615.69 | | 12383705.87 | | 14892719.11 | | 14635184.69 | | 13884382.64 | |  |  |  |
| Ranolazine Dihydrochloride | | 21903744.4 | | 22081537.51 | | 21760728.84 | | 24310362.05 | | 27681249.48 | | 25077230.74 | |  |  |  |
| Glyasperin D | | 24098541.14 | | 26236841.97 | | 24007962.83 | | 30330053.4 | | 29704681.53 | | 28226370.53 | |  |  |  |
| Cloperastine Hydrochloride | | 109641585 | | 126292449.3 | | 135132669.2 | | 157247480 | | 145428423.7 | | 142841093.3 | |  |  |  |
| Hordenine | | 63652233.91 | | 70930116.36 | | 61243247.61 | | 83351557.51 | | 80472251.8 | | 72052417.19 | |  |  |  |
| 5,7-Dihydroxyisoflavone | | 71823256.15 | | 57950901.72 | | 62175274.8 | | 79021478.5 | | 76595180.57 | | 75709282.11 | |  |  |  |
| Styrene | | 180056705.8 | | 189276013.7 | | 183203324.8 | | 217843147 | | 224653172.1 | | 225561007.2 | |  |  |  |
| Lovastatin | | 18466029.89 | | 20165249.84 | | 19162634.1 | | 21062579.15 | | 25503651.95 | | 23588339.04 | |  |  |  |
| Perospirone | | 24255165.29 | | 27484096.31 | | 26728891.46 | | 31329986.49 | | 34629086.57 | | 29303188.01 | |  |  |  |
| **MetaboName** | | | | | **SMM914-1** | | **SMM914-2** | | **SMM914-3** | | **CONTROL-1** | | **CONTROL-2** | | **CONTROL-3** | |
| Daidzin | | 296315846.9 | | 327779311.6 | | 350269781.6 | | 405517471.4 | | 387329851.8 | | 392401359.9 | |  |  |  |
| Santonin | | 2692256343 | | 2989228863 | | 2874303042 | | 3245835709 | | 3559214005 | | 3615003538 | |  |  |  |
| Isovaleric Acid | | 22171791.59 | | 23024111.17 | | 24669167.2 | | 29086706.39 | | 28337670.4 | | 28127629.63 | |  |  |  |
| Paxilline | | 15624649.82 | | 14597739.97 | | 14280391.27 | | 18545873.14 | | 18379382.21 | | 17630806.47 | |  |  |  |
| Isosungucine | | 81980347.22 | | 79123495.37 | | 81355819.07 | | 104670541.5 | | 94959438.62 | | 98637684.85 | |  |  |  |
| Galactitol | | 145756155 | | 137306383.2 | | 127905741.6 | | 172821575.4 | | 163608192 | | 175963087.9 | |  |  |  |
| Stigmasterol Glucoside | | 1737527877 | | 1851664175 | | 1869537526 | | 2280526628 | | 2234370697 | | 2306205786 | |  |  |  |
| Neocuproine | | 438861909.9 | | 457134850 | | 455900969.8 | | 563010201.9 | | 576897526.5 | | 551420385.6 | |  |  |  |
| Vindolinine | | 11727354.41 | | 12070860.98 | | 12668453.15 | | 16391685.1 | | 14906910.23 | | 14756953.05 | |  |  |  |
| Uridine 5'-Monophosphate | | 510240718.6 | | 407818433.7 | | 409445447.2 | | 579990551.8 | | 554005567.6 | | 556340232.4 | |  |  |  |
| Phenethylamine | | 218414775.3 | | 218607029 | | 208021216.5 | | 272599358.7 | | 272068608.3 | | 279694397 | |  |  |  |
| Cytidine-3'-Monophosphate | | 186092252.8 | | 149365715.3 | | 142332627.3 | | 214282247.3 | | 204309408.8 | | 196521447.9 | |  |  |  |
| Benzphetamine | | 293786182.1 | | 360734719 | | 350189266.4 | | 392034936.1 | | 433122789.5 | | 479501466.2 | |  |  |  |
| Cordycepin | | 22524678.54 | | 21220724.88 | | 21226266.02 | | 29858003.79 | | 26841357.04 | | 28080976.53 | |  |  |  |
| N-2-Fluorenylacetamide | | 422194239.1 | | 345209457.2 | | 335765503.3 | | 488058225 | | 501361954.5 | | 455834225.1 | |  |  |  |
| Benzene-1,2,4-Triol | | 267785589.8 | | 213528162.9 | | 230835509.9 | | 327517472.2 | | 331703587.8 | | 274654574.6 | |  |  |  |
| Adenosine 3',5'-Cyclic Monophosphate | | 66031830.82 | | 56404009.89 | | 52674454.04 | | 79673602.74 | | 78036061.55 | | 72901347.68 | |  |  |  |
| L-Alanine | | 7309336364 | | 6168521127 | | 5805105299 | | 8644145371 | | 8645956804 | | 8193595127 | |  |  |  |
| 2,3-Dideoxycytidine | | 3697697435 | | 4048216017 | | 3874896449 | | 4954395800 | | 5199041879 | | 5207978991 | |  |  |  |
| Chlorovirensic Acid | | 27090672.08 | | 23256801.76 | | 20704143.5 | | 33260655.24 | | 30981045.73 | | 30249886.39 | |  |  |  |
| **MetaboName** | | **SMM914-1** | | **SMM914-2** | | **SMM914-3** | | **CONTROL-1** | | **CONTROL-2** | | **CONTROL-3** | |  |  |  |
| 3-Hydroxy-1,2-Dimethylpyridin-4(1H)-One | | 258020358.1 | | 260866193.3 | | 304456998 | | 352931085.7 | | 345844954.4 | | 398777211.1 | |  |  |  |
| Mulberroside F | | 5125631.19 | | 5586834.66 | | 5651766.5 | | 7802624.17 | | 7103419.2 | | 6970342.82 | |  |  |  |
| Otenzepad | | 40518500.36 | | 32813627.42 | | 28306818.09 | | 46840578.83 | | 42554153.54 | | 47031031.69 | |  |  |  |
| 2-Oxobutyric Acid | | 2440470961 | | 1998979674 | | 1940582668 | | 2674625158 | | 2840184436 | | 3073064967 | |  |  |  |
| N-Acetyl-L-phenylalanine | | 333167720.6 | | 253046784.7 | | 236426283.5 | | 363650489.5 | | 368743434.8 | | 379327796.7 | |  |  |  |
| Allantoin | | 400394332.1 | | 326470811.1 | | 318226942.9 | | 474013253.4 | | 479278552.5 | | 459302379.2 | |  |  |  |
| O-Phospho-L-serine | | 90886618.22 | | 83031917.11 | | 78874709.82 | | 114556528.6 | | 114835866.1 | | 113003897.9 | |  |  |  |
| Phenylalanine | | 27227681771 | | 23169781025 | | 21463145550 | | 33099164476 | | 32023472483 | | 32292545470 | |  |  |  |
| Cinnamoylcholine | | 74986419.84 | | 69167505.27 | | 76146714.36 | | 106745041.7 | | 101290449.8 | | 91038749.85 | |  |  |  |
| D-Arabitol | | 12629838903 | | 11654712004 | | 11209721091 | | 15820613651 | | 16032100293 | | 16477966654 | |  |  |  |
| N-Acetyl-L-Aspartic Acid | | 1453621473 | | 1144524377 | | 1115795247 | | 1755297990 | | 1721233224 | | 1594355895 | |  |  |  |
| Isotaxiresinol | | 18965177.4 | | 16354119.41 | | 16950879.92 | | 26719312.74 | | 23219520.55 | | 22178568.41 | |  |  |  |
| N-Homoveratroylhomoveratrylamine | | 67661895.61 | | 72640529.93 | | 70005102.59 | | 106887685.8 | | 100076413.6 | | 83627037.01 | |  |  |  |
| Zileuton | | 472194906.2 | | 384389349.4 | | 371800114 | | 585647384.4 | | 582064626.5 | | 533868482.3 | |  |  |  |
| S-Adenosyl-L-homocysteine | | 141906998.1 | | 117687339.1 | | 123470697.1 | | 179983204.7 | | 150383205.7 | | 206051268.9 | |  |  |  |
| Feruloyl Lactate | | 418446537.1 | | 332210880.7 | | 329184848.8 | | 526258727.7 | | 484128032.8 | | 502369618.8 | |  |  |  |
| N-(2-Aminobenzoyl)Alanine | | 83447405.98 | | 96973051.48 | | 96706437.28 | | 127475441.1 | | 134043727.5 | | 126928259.5 | |  |  |  |
| Ibuprofen | | 17403055.61 | | 12459480.71 | | 11768114.76 | | 21085635.13 | | 17990170.02 | | 19437793.72 | |  |  |  |
| 2,3-Dideoxyuridine | | 590513256 | | 446988031.7 | | 431072088.3 | | 738438140 | | 673607855.4 | | 652232342 | |  |  |  |
| **MetaboName** | | | **SMM914-1** | | **SMM914-2** | | **SMM914-3** | | **CONTROL-1** | | **CONTROL-2** | | **CONTROL-3** | |  |  |
| Pyrrolnitrin | | 2564676025 | | 2297510670 | | 2036305873 | | 3250165031 | | 3288536867 | | 3221853475 | |  |  |  |
| Sulfamethoxazole | | 2080285493 | | 1758577834 | | 1665359913 | | 2695306228 | | 2580505212 | | 2518713257 | |  |  |  |
| Carnosine | | 760618204.8 | | 594212463 | | 594254647 | | 977263077.8 | | 917214161.1 | | 867358090.3 | |  |  |  |
| Myclobutanil | | 55398287.48 | | 56321335.72 | | 56875340.67 | | 79647192.67 | | 83919568.99 | | 76186912.26 | |  |  |  |
| D-Glucosamine 6-Phosphate | | 24025799.4 | | 20703873.66 | | 19130358.71 | | 31440447.08 | | 28312189.34 | | 31227779.94 | |  |  |  |
| 2-Chloro-6-O-Methylnorlichexanthone | | 10947187678 | | 9189449373 | | 8778152191 | | 14215183898 | | 13660227171 | | 13435525483 | |  |  |  |
| Perfluorobutane Sulfonamido Amine | | 65428301.04 | | 59369322.33 | | 51637550.44 | | 79666461.72 | | 90712997.16 | | 82256664.78 | |  |  |  |
| Licochalcone B | | 256982973 | | 199372183.1 | | 181063479.5 | | 311689600 | | 290708918.5 | | 311924339.8 | |  |  |  |
| L-Lysine | | 11756870598 | | 9211786901 | | 9345757836 | | 14922682021 | | 14425708185 | | 14185549995 | |  |  |  |
| Mannose | | 2963788763 | | 2480770278 | | 2362737732 | | 3511394637 | | 3665156958 | | 4042794491 | |  |  |  |
| Salazinic Acid | | 6729314656 | | 5616935155 | | 5337675671 | | 8814144036 | | 8342157125 | | 8263737053 | |  |  |  |
| PFSA-H | | 27584039.65 | | 23152767.39 | | 20736658.46 | | 35850197.53 | | 33320029.9 | | 33646468.87 | |  |  |  |
| Phenacylamine Hydrochloride | | 6318040355 | | 5349282416 | | 4989353583 | | 8328848241 | | 7996980649 | | 7687467698 | |  |  |  |
| Hypoxanthine | | 12562512874 | | 14206624695 | | 13366237478 | | 17569388916 | | 20357313891 | | 19937964858 | |  |  |  |
| Uracil | | 10248493485 | | 8441437491 | | 8638420966 | | 12016883045 | | 12079184493 | | 15427509765 | |  |  |  |
| (S,S)-Tartaric Acid | | 49426448910 | | 38100252406 | | 38550184547 | | 62419028101 | | 60790608170 | | 59232542714 | |  |  |  |
| Imidazole-4-acetate | | 726596270.1 | | 556778672.5 | | 489217062.4 | | 796887715.9 | | 984145629.5 | | 791066206.2 | |  |  |  |
| Paracetamol | | 94094930.03 | | 82842441.08 | | 77820537.33 | | 116362569 | | 123848945 | | 131071794.8 | |  |  |  |
| Anserine | | 52078876.44 | | 42788304.54 | | 40842062.89 | | 68371685.17 | | 64479538.68 | | 65006723.55 | |  |  |  |
| **MetaboName** | | **SMM914-1** | | **SMM914-2** | | **SMM914-3** | | **CONTROL-1** | | **CONTROL-2** | | **CONTROL-3** | |  |  |  |
| Dehydroeburicoic Acid Monoacetate | | 429572142.2 | | 440559491.4 | | 459333579.1 | | 648338044.4 | | 667712856.1 | | 639248548 | |  |  |  |
| Skimmin | | 235415713.2 | | 180300826.3 | | 177096916 | | 309869399.4 | | 286811743.3 | | 275301875.8 | |  |  |  |
| Thymine | | 277936539.2 | | 228182904.4 | | 213483815.8 | | 370747961.3 | | 394269947.9 | | 299583625.3 | |  |  |  |
| Sophoridine | | 106388717.1 | | 106916249.2 | | 105567770.4 | | 166483965.5 | | 146580757.4 | | 159091143.7 | |  |  |  |
| N-Acetyl-L-leucine | | 720902983.5 | | 561666639.6 | | 540378972.2 | | 903779513 | | 908065970.7 | | 892086565.5 | |  |  |  |
| 2-Hyroxybiphenyl | | 238894621.7 | | 422738225.4 | | 409049972.8 | | 552858607.2 | | 524175352 | | 511838395.6 | |  |  |  |
| Lecanoric Acid | | 109290619.2 | | 83064875.48 | | 77075489.83 | | 157005770.8 | | 123483544.7 | | 119616118.4 | |  |  |  |
| Nornicotine | | 22994594.94 | | 22836165.72 | | 21316501.54 | | 33415973.49 | | 34505561.76 | | 31866396.86 | |  |  |  |
| Tetrasaccharides | | 43221393.24 | | 30632736.64 | | 36743898.69 | | 56565898.41 | | 51586205.34 | | 56322566.64 | |  |  |  |
| Dl-3-Aminoisobutyric Acid | | 2099627305 | | 1674883276 | | 1685005683 | | 2777331804 | | 2676680937 | | 2672937117 | |  |  |  |
| Deguelin(-) | | 374322624.6 | | 308282384.4 | | 311413956.3 | | 508977518.9 | | 478897444.3 | | 500931276 | |  |  |  |
| Serine | | 3036147243 | | 2486716399 | | 2383868324 | | 4027984351 | | 4050104799 | | 3875339345 | |  |  |  |
| Oxaloacetate | | 34075599.19 | | 23228328.12 | | 24312205.95 | | 43521300.58 | | 40257310.71 | | 39657626.63 | |  |  |  |
| L-Arginine | | 29782496327 | | 28850954985 | | 30147472896 | | 46841000221 | | 45761568184 | | 42070464239 | |  |  |  |
| 3-Acetylthiazolidine-4-Carboxylic Acid | | 208112051.6 | | 218071681.8 | | 214743427.2 | | 306997684.1 | | 331396378.2 | | 336165709.5 | |  |  |  |
| Vitamine A Acetate | | 8796503.48 | | 7279978.53 | | 5744549.15 | | 11459933.42 | | 10856311.64 | | 10992959.14 | |  |  |  |
| Fipronil Sulfone | | 1699872381 | | 1480831366 | | 1316982120 | | 2322350704 | | 2315325909 | | 2238835338 | |  |  |  |
| Heptadecanoic Acid | | 45241688.24 | | 39518621.82 | | 27092000.16 | | 56627811.03 | | 56897603.67 | | 57930870.82 | |  |  |  |
| Piplartine | | 831025864.9 | | 688984242.9 | | 646176039.6 | | 1070944032 | | 1121402959 | | 1139639689 | |  |  |  |
| 3-Hydroxyanthranilic Acid | | 286164395.4 | | 332745534.6 | | 305018980 | | 433952928.4 | | 512639441.1 | | 476494834.8 | |  |  |  |
| **MetaboName** | | **SMM914-1** | | **SMM914-2** | | **SMM914-3** | | **CONTROL-1** | | **CONTROL-2** | | **CONTROL-3** | |  |  |  |
| Fraxinellone | | 145613474.3 | | 151556680.7 | | 151609034.1 | | 223617940.9 | | 238556747.5 | | 231846516.2 | |  |  |  |
| L-Saccharopine | | 18655561.17 | | 14353211.14 | | 14564205.12 | | 26224473.97 | | 24514902.16 | | 22880962.56 | |  |  |  |
| Acetyl Isogambogic Acid | | 2872251.04 | | 4216603.5 | | 3799585.82 | | 5644044.93 | | 6421942.15 | | 4795153.33 | |  |  |  |
| Indole-3-Acetyl-L-Phenylalanine | | 35420479.23 | | 35645989.1 | | 30831166.88 | | 54405046.42 | | 49468609.29 | | 54067872.98 | |  |  |  |
| Vinetorin | | 183839845.4 | | 144426636.5 | | 147144383 | | 248258653.6 | | 242546616 | | 246205994.4 | |  |  |  |
| Cytidine 5'-Diphosphate | | 7871821.51 | | 5990114.62 | | 5333182.09 | | 10368791.81 | | 10670468.4 | | 8730063.49 | |  |  |  |
| Homovanillic Acid | | 1527464681 | | 1190602780 | | 1201483272 | | 1918326848 | | 1911060967 | | 2254412708 | |  |  |  |
| Parfumine | | 11175897.74 | | 9279121.64 | | 10028341.39 | | 16377135.99 | | 16009758.33 | | 14958815.28 | |  |  |  |
| Reserpine | | 14485409.42 | | 15037371.48 | | 15187423.27 | | 24520484.95 | | 23041340.8 | | 22108491.72 | |  |  |  |
| Quercetin 3-O-Malonylglucoside | | 796279635.4 | | 688592173.9 | | 618248630.9 | | 1109561823 | | 1106493704 | | 1067994844 | |  |  |  |
| 4-Hydroxyquinoline | | 377999263.5 | | 299198935.8 | | 281666548.3 | | 510971724.9 | | 499142789.4 | | 496710297.3 | |  |  |  |
| Obtusaquinone | | 193770246.9 | | 166883642.6 | | 159280544.8 | | 269662643.5 | | 274166206.7 | | 273554619 | |  |  |  |
| Formononetin | | 377922230.5 | | 248862759.7 | | 277381208.3 | | 457450210.6 | | 443209443.4 | | 525707735.7 | |  |  |  |
| Glycyl-L-Proline | | 134349547.1 | | 188118918.6 | | 176719350.2 | | 250390422.6 | | 276035094.1 | | 265451776.6 | |  |  |  |
| 2',4'-Dihydroxychalcone | | 732562444.1 | | 579708898.3 | | 529652547.3 | | 980614384.1 | | 993628978.9 | | 953458873.7 | |  |  |  |
| Atomoxetine | | 311001130.9 | | 324032777.5 | | 296174548.7 | | 444862665.3 | | 508394894.7 | | 531044503.4 | |  |  |  |
| 5-Hydroxy-L-Tryptophan | | 871950436.3 | | 694238543.2 | | 670922646.6 | | 1109359396 | | 1179857086 | | 1286987040 | |  |  |  |
| Gmelinol | | 101790394.6 | | 77172722.77 | | 68259970.16 | | 142078277.6 | | 127205231.1 | | 127383316.8 | |  |  |  |
| L-Leucine | | 37392212225 | | 31794939708 | | 30565230966 | | 53805192061 | | 54888425058 | | 51909037769 | |  |  |  |
| Phosphoric Acid | | 6551552895 | | 5485322614 | | 5286647090 | | 9227951365 | | 9431344593 | | 9255938882 | |  |  |  |
| **MetaboName** | | **SMM914-1** | | **SMM914-2** | | **SMM914-3** | | **CONTROL-1** | | **CONTROL-2** | | **CONTROL-3** | |  |  |  |
| Cymarine | | 9625939.83 | | 8962736.81 | | 9609117.34 | | 13610032.93 | | 16993908.19 | | 14878688.1 | |  |  |  |
| Harmine | | 35466581.68 | | 23570726.09 | | 22099230.62 | | 42230612.93 | | 45569678.31 | | 43340583.7 | |  |  |  |
| N-Isovalerylglycine | | 770036910.3 | | 602755405.4 | | 575676381.7 | | 1052183706 | | 1083119869 | | 1027613845 | |  |  |  |
| Coralyne Chloride | | 19405522.97 | | 15405473.18 | | 15652958.9 | | 25606099.46 | | 25242559.6 | | 31718256.9 | |  |  |  |
| Muramic Acid | | 519776904.9 | | 405348410.1 | | 381946211.5 | | 738137790.2 | | 709940740.4 | | 692238635.2 | |  |  |  |
| Zearalenol | | 195090325.9 | | 154020580.1 | | 168028226.8 | | 330210544.2 | | 260137031.8 | | 276602129.7 | |  |  |  |
| Isoxicam | | 31655646.2 | | 30344961.52 | | 32218336.51 | | 57855982.56 | | 50455749.83 | | 50144117.18 | |  |  |  |
| Parthenolide | | 23089647.48 | | 28011862.82 | | 26247987 | | 38372601.1 | | 47240686.23 | | 44530771.95 | |  |  |  |
| Dethiobiotin | | 206614156.8 | | 180389102.7 | | 154086017.4 | | 291613110 | | 298300064.4 | | 327838966.4 | |  |  |  |
| Conocarpan | | 9211152.74 | | 5072816.55 | | 7053741.13 | | 12918699.1 | | 12627045.61 | | 10874211.36 | |  |  |  |
| Columbamine | | 52324169.06 | | 53637708.64 | | 51075448.57 | | 87318483.11 | | 87980435.45 | | 92801532.72 | |  |  |  |
| Purine | | 5502644292 | | 4550484868 | | 4355533620 | | 8560202139 | | 8472736887 | | 7778191549 | |  |  |  |
| 3-Methylxanthine | | 979451771.6 | | 801923916.3 | | 700899601.6 | | 1475741517 | | 1560430315 | | 1259331634 | |  |  |  |
| Resveratrol | | 766090193.2 | | 625724981.9 | | 598782712 | | 1133220631 | | 1149067749 | | 1164678422 | |  |  |  |
| Dihydro-4,4-Dimethyl-2,3-Furandione | | 245572233.3 | | 202766037.4 | | 177109952.3 | | 348052044.4 | | 376790417.2 | | 359500358.9 | |  |  |  |
| 2-Hydroxybenzaldehyde | | 713356309.3 | | 648554755.2 | | 609048031.5 | | 1116206413 | | 1106617682 | | 1194406591 | |  |  |  |
| Carbamimidothioic Acid | | 29980073.74 | | 20784035 | | 39295738.44 | | 54526068.06 | | 46679521.17 | | 55345622.11 | |  |  |  |
| D-Mannitol | | 144639672.2 | | 158118929.9 | | 186842047.8 | | 322379344.4 | | 272589178.5 | | 256171216.7 | |  |  |  |
| Hippurate | | 50534865.6 | | 44725269.49 | | 45168033.26 | | 77222960.07 | | 79208957.17 | | 88307081.92 | |  |  |  |
| Rebemide | | 306834372.5 | | 332433694.4 | | 354793137.8 | | 656709455.9 | | 600464990.6 | | 481808902.3 | |  |  |  |
| **MetaboName** | | **SMM914-1** | | **SMM914-2** | | **SMM914-3** | | **CONTROL-1** | | **CONTROL-2** | | **CONTROL-3** | |  |  |  |
| Dracorhodin Perchlorate | | 65212218.77 | | 61268984.64 | | 64059814.43 | | 112903281.4 | | 111505236.3 | | 108960360.2 | |  |  |  |
| Sinapate | | 75046755.87 | | 58261031.41 | | 59274675.77 | | 115588138.1 | | 117067249.6 | | 106738296.6 | |  |  |  |
| Ritonavir | | 6266031.48 | | 4696946.54 | | 4242683.54 | | 8525535.3 | | 10567777.8 | | 7720466.79 | |  |  |  |
| Secobarbital | | 13564647.15 | | 10914161.04 | | 9964347.87 | | 19244419.1 | | 21384076.5 | | 20148659.8 | |  |  |  |
| 4-Nitroquinoline 1-Oxide | | 163026537.9 | | 124249168 | | 118108735.4 | | 242114118.1 | | 234019094.4 | | 240934516.1 | |  |  |  |
| Methylnissolin-3-O-Glucoside | | 4343934.49 | | 4036638.79 | | 4641759.57 | | 8542428.06 | | 7457889.6 | | 7092504.47 | |  |  |  |
| Darutoside | | 52703936 | | 54091652.33 | | 51087935.05 | | 95922127.33 | | 96723406.06 | | 88266865.94 | |  |  |  |
| Pyridoxamine | | 144552897.6 | | 146325659.5 | | 143879239.5 | | 247877147.5 | | 267180876.3 | | 262866894.7 | |  |  |  |
| Phthalic Anhydride | | 97489460.31 | | 98522636.72 | | 103665086.2 | | 176950938.4 | | 189259498.9 | | 184371013.5 | |  |  |  |
| Bupropion | | 198401529.2 | | 148312887.7 | | 127733072.1 | | 289624775.4 | | 280112043.1 | | 317329577.7 | |  |  |  |
| 3-Hydroxybenzaldehyde | | 413250054 | | 313161413.1 | | 279954527 | | 578200032.8 | | 771291174.5 | | 535378226.2 | |  |  |  |
| L-2-Aminoadipic Acid | | 1719616566 | | 1540557910 | | 1457604093 | | 2953968071 | | 2888081500 | | 3014106304 | |  |  |  |
| Norepinephrine | | 527966020.4 | | 435055413.6 | | 456822141 | | 895784161 | | 866175978.7 | | 907031562.6 | |  |  |  |
| Phlorobenzophenone | | 81765996.35 | | 67183022.75 | | 67315851.76 | | 140803974.7 | | 128762242.4 | | 139116546.7 | |  |  |  |
| Tyrosine | | 6207286438 | | 6445751714 | | 6521677127 | | 12412391972 | | 12321202619 | | 11973265903 | |  |  |  |
| L-Tyrosine | | 4436229003 | | 3688231250 | | 3646728640 | | 7519097246 | | 7628704970 | | 7452398069 | |  |  |  |
| Malvidin-3-O-Glucoside | | 123500770.4 | | 95634031.5 | | 92366870.75 | | 176914268.8 | | 208252466.8 | | 223979242.8 | |  |  |  |
| D-Glyceric Acid | | 2654694937 | | 2126914382 | | 2060616957 | | 4339769598 | | 4501860397 | | 4564232282 | |  |  |  |
| 5-Methylcytosine | | 135800685.9 | | 138666066.3 | | 134546150.6 | | 284265399.4 | | 282252632.2 | | 242194969.8 | |  |  |  |
| Triacetin | | 51978740.23 | | 48491948 | | 31800471.86 | | 95668143.76 | | 80260709.08 | | 90469317.72 | |  |  |  |
| **MetaboName** | | **SMM914-1** | | **SMM914-2** | | **SMM914-3** | | **CONTROL-1** | | **CONTROL-2** | | **CONTROL-3** | |  |  |  |
| Imatinib | | 52292089.76 | | 40861775.72 | | 41418531.64 | | 89051922.16 | | 96475590.56 | | 89171790.54 | |  |  |  |
| Surugamidea | | 276945868.1 | | 289318005.5 | | 326256771.6 | | 649128164.4 | | 603345483.2 | | 571976681.3 | |  |  |  |
| Oleic Acid | | 2292126652 | | 1901284090 | | 1824525852 | | 4022578053 | | 4089507508 | | 4197950915 | |  |  |  |
| Luvangetin | | 26306884.12 | | 23001264.61 | | 21822040.48 | | 48621393.04 | | 48227106.7 | | 48899692.75 | |  |  |  |
| 9-Trans-Palmitelaidic Acid | | 42946729.95 | | 45387595.06 | | 36936615.84 | | 86798252.02 | | 85370714.94 | | 85094093.73 | |  |  |  |
| 3-(4-Hydroxyphenyl)Lactate | | 56360170.42 | | 39156577.86 | | 78861924.97 | | 119335065.5 | | 123581391.7 | | 116399488.7 | |  |  |  |
| Nomegestrol Acetate | | 14931944.11 | | 13751537.12 | | 15208801.67 | | 29872111.02 | | 29135493.72 | | 31689235.31 | |  |  |  |
| (-)-Citramalic Acid | | 612180358.6 | | 466862261 | | 467704485.5 | | 1050092309 | | 1154753576 | | 991832625.2 | |  |  |  |
| Phenylacetic Acid | | 187516005.4 | | 151232595.3 | | 124255711 | | 305168631.7 | | 359695561.6 | | 292650878.5 | |  |  |  |
| Ononetin | | 90101013.12 | | 66667532.81 | | 64600133.28 | | 152006735.6 | | 147931469.9 | | 158838471.7 | |  |  |  |
| Daidzein | | 143642489.9 | | 112493331 | | 114825345.5 | | 243825445.9 | | 258147590.6 | | 267209118.1 | |  |  |  |
| Corymbosin | | 605981074.5 | | 555466038.3 | | 495892826.2 | | 1163377267 | | 1187809534 | | 1091943879 | |  |  |  |
| Clocortolone Pivalate | | 61408274.39 | | 72701229.93 | | 82205983.04 | | 137136099.9 | | 149710844.7 | | 163304269 | |  |  |  |
| Angeloylgomisin H | | 6603384.44 | | 5079959.14 | | 4971096.19 | | 13844391.54 | | 11211434.94 | | 9912522.42 | |  |  |  |
| 4-Pyridoxic Acid | | 240442647.2 | | 180145484.7 | | 163476165.2 | | 421568236.5 | | 432775915.8 | | 384639539.3 | |  |  |  |
| D-Fructose | | 1492836698 | | 1253678071 | | 1199108012 | | 2934794764 | | 2876282701 | | 2709106820 | |  |  |  |
| Thiamine Monophosphate | | 158972459.9 | | 129930460.4 | | 131062417.9 | | 308409602.4 | | 305545550 | | 299238243 | |  |  |  |
| beta-Ureidopropionic acid | | 1807927473 | | 1856974176 | | 1868977891 | | 3935700527 | | 4084394866 | | 4046212187 | |  |  |  |
| Penitrem A | | 2744268.85 | | 2113804.21 | | 2747172.81 | | 5996465.19 | | 5236653.35 | | 5382038.03 | |  |  |  |
| Benzoic Acid | | 192720561.4 | | 205987752.1 | | 203706098.8 | | 448500251.1 | | 446996161 | | 449233465.5 | |  |  |  |
| **MetaboName** | | **SMM914-1** | | **SMM914-2** | | **SMM914-3** | | **CONTROL-1** | | **CONTROL-2** | | **CONTROL-3** | |  |  |  |
| 3-Aminoacetophenone | | 585423957.2 | | 591196991.8 | | 610398894.5 | | 1358397905 | | 1325449444 | | 1321939277 | |  |  |  |
| 5'-Methylthioadenosine | | 3077746757 | | 2433812546 | | 2534911905 | | 6691700159 | | 5908110587 | | 5656200569 | |  |  |  |
| 2,4-Dinitrophenol | | 651950170.1 | | 612000186.7 | | 535963862.5 | | 1316044218 | | 1507796716 | | 1271009586 | |  |  |  |
| Butyric Acid | | 727273382.2 | | 956667779.3 | | 835182837.6 | | 1886882691 | | 2001893947 | | 1928173244 | |  |  |  |
| Moclobemide | | 163193387.4 | | 197489845.1 | | 50348569.81 | | 329735024.9 | | 323055343.2 | | 318727016.8 | |  |  |  |
| Puwainaphycin C | | 57390161.86 | | 55793888.06 | | 61256587.84 | | 146163096.6 | | 142976691.9 | | 123712413.1 | |  |  |  |
| Chloratranol | | 216241215.2 | | 197053723.7 | | 170156945 | | 448094589.1 | | 499820728.5 | | 434933212.6 | |  |  |  |
| Salicylamide | | 66047070.6 | | 54526890.95 | | 83721041.98 | | 182372798.2 | | 185021231.8 | | 128870508 | |  |  |  |
| Deacetylgedunin | | 47005859.89 | | 36555364.08 | | 3497260.48 | | 70414430.84 | | 76607831 | | 67079768.96 | |  |  |  |
| 1,4-Naphthalene-Dione | | 824163325 | | 510200165.8 | | 712135150.8 | | 1692518858 | | 1619645153 | | 1744117915 | |  |  |  |
| Gentiobiose | | 183169319.9 | | 189081090.5 | | 186741001 | | 435032851 | | 467446396.2 | | 479341885.3 | |  |  |  |
| Methylprednisolone | | 32917566.42 | | 32704433.38 | | 36668323.27 | | 84175321.5 | | 88527865.97 | | 81740605.19 | |  |  |  |
| Glutamine | | 339252659 | | 358846518.5 | | 356244097.1 | | 825041926.7 | | 920247066 | | 904221666.7 | |  |  |  |
| 1,4-Butynediol | | 243865217.9 | | 247647506.3 | | 282002551.7 | | 592394041.7 | | 655497176.7 | | 728591319.2 | |  |  |  |
| Valeramide | | 369429202.1 | | 399007362.7 | | 391370166.8 | | 1011946060 | | 1084592712 | | 930760332 | |  |  |  |
| Indole-3-Carboxylic Acid | | 42411354.21 | | 39263031.87 | | 30677829.36 | | 98834105.86 | | 92632833.27 | | 104471249.6 | |  |  |  |
| Proline-Hydroxyproline | | 1516960108 | | 1182783648 | | 523643116.8 | | 2995739779 | | 2945850245 | | 2682632303 | |  |  |  |
| Licoricidin | | 5008448.81 | | 4241378.58 | | 4047613.63 | | 13094890.08 | | 12150823.62 | | 10925540.15 | |  |  |  |
| Substance P | | 7655611.93 | | 9890238.85 | | 7872698.95 | | 15718277.55 | | 25015261.4 | | 30271504.63 | |  |  |  |
| Cephalochromin | | 14947117.86 | | 9786472.3 | | 10269377.25 | | 35646871.2 | | 33371074.73 | | 30694162.2 | |  |  |  |
| **MetaboName** | | **SMM914-1** | | **SMM914-2** | | **SMM914-3** | | **CONTROL-1** | | **CONTROL-2** | | **CONTROL-3** | |  |  |  |
| Taurine | | 476372913.6 | | 441538499.4 | | 404022714 | | 1252505272 | | 1318924557 | | 1239883850 | |  |  |  |
| Dendrobine | | 74373777.18 | | 79528600.04 | | 74720637.71 | | 222599593.5 | | 202363708 | | 234622854.6 | |  |  |  |
| MCPA | | 593994391.5 | | 525525669.6 | | 426624484.7 | | 1527058271 | | 1690291018 | | 1265621665 | |  |  |  |
| Beclomethasone | | 29994814.71 | | 22674817.41 | | 21496716.7 | | 75706715.45 | | 69063636.82 | | 72063147.87 | |  |  |  |
| 7-Acetoxycoumarin | | 14732727.92 | | 15894819.82 | | 14591819 | | 43454176.74 | | 51709660.32 | | 47016110.6 | |  |  |  |
| Lamalbid | | 24551785.46 | | 18031511.24 | | 20277301.9 | | 65029449.99 | | 63359327.34 | | 71143640.88 | |  |  |  |
| Homatropine Hydrobromide (R,S) | | 67529349.17 | | 66361716.83 | | 70550746.82 | | 223225655.1 | | 217786116.7 | | 210860595.3 | |  |  |  |
| D-Saccharic Acid | | 82072364.3 | | 69949346.62 | | 55218521.66 | | 224017023.9 | | 229258543.7 | | 208546935.8 | |  |  |  |
| Methioninesulfoxide | | 1034042044 | | 811270091 | | 861173689.1 | | 2947797897 | | 2911005414 | | 2864463371 | |  |  |  |
| L-Asparagine | | 1418092974 | | 1134236384 | | 1067970575 | | 3940201581 | | 3906863793 | | 3821812279 | |  |  |  |
| Trinitrotoluene | | 143379371.1 | | 131465764.3 | | 68930461.97 | | 394466822.9 | | 404643834.5 | | 335044692 | |  |  |  |
| 5-Methoxypsoralen | | 520679974.9 | | 435187025.7 | | 374151003.5 | | 1441317491 | | 1662297184 | | 1326462773 | |  |  |  |
| Obtucarbamate A | | 1713631.81 | | 20203207.63 | | 19289778.08 | | 43319321.84 | | 47855880.49 | | 49306999.19 | |  |  |  |
| Guanine | | 214215086.1 | | 174609322.5 | | 174409249.4 | | 650725907.4 | | 686372164.3 | | 611820973.8 | |  |  |  |
| Olmesartan | | 27732267.4 | | 23812633.54 | | 25077389.74 | | 85626107.48 | | 90986654.16 | | 90247727.23 | |  |  |  |
| D-(+)-Pantothenic Acid | | 151858693 | | 125949125.3 | | 133057768.8 | | 482391356.4 | | 478147945 | | 473433941.9 | |  |  |  |
| Fenoldopam | | 120913047.8 | | 107033871 | | 100384417.7 | | 392884094.6 | | 412720792.5 | | 357227169.8 | |  |  |  |
| Indole-3-Acetyl-L-Tryptophan | | 33322239.46 | | 20980292.62 | | 19850033.17 | | 97639999.87 | | 87019497.25 | | 83246762.41 | |  |  |  |
| 2,3-Dihydroxybiphenyl | | 257981160.9 | | 200566792.6 | | 197833363.3 | | 784047434.8 | | 796436158.5 | | 804476379.5 | |  |  |  |
| Glycodeoxycholic Acid | | 3946681.63 | | 3915435.89 | | 3791099.15 | | 12963552.24 | | 15327396.28 | | 15085526.55 | |  |  |  |
| **MetaboName** | | **SMM914-1** | | **SMM914-2** | | **SMM914-3** | | **CONTROL-1** | | **CONTROL-2** | | **CONTROL-3** | |  |  |  |
| Quinolinic Acid | | 61538708.21 | | 17657944.99 | | 1018259.84 | | 92851691.09 | | 99622247.27 | | 106326706.3 | |  |  |  |
| Val-Leu-Pro-Val-Pro | | 139127944.4 | | 119503209 | | 131693681.4 | | 484566706.1 | | 493963061.1 | | 495970783.7 | |  |  |  |
| Rhizocarpic Acid | | 6048465.51 | | 4910101.03 | | 4436761.06 | | 19613459.45 | | 20727292.5 | | 17824061.95 | |  |  |  |
| 6-Methoxyflavonol | | 22121722.23 | | 19700981.72 | | 19764486.58 | | 91128823.89 | | 66165646.42 | | 77324709.34 | |  |  |  |
| Lariciresinol | | 36097019.51 | | 29279841.89 | | 27001105.6 | | 126765036.9 | | 124512250.8 | | 108183005.4 | |  |  |  |
| Ilicicolin A | | 2654642.31 | | 2986676.84 | | 112459255.6 | | 171907250.2 | | 127713026.7 | | 172087095.4 | |  |  |  |
| Caffeic Acid Hexoside | | 68851398.43 | | 52391947.48 | | 46886450.21 | | 220675245.8 | | 228700506.8 | | 227274759.1 | |  |  |  |
| 2'-Deoxyinosine | | 258558620.7 | | 222187151.4 | | 903844916.1 | | 1756188963 | | 1692540536 | | 2427759080 | |  |  |  |
| N-Acetylneuraminate | | 10490884000 | | 8494679179 | | 8285779961 | | 39543715280 | | 38998104613 | | 38942000022 | |  |  |  |
| PFAP-N Pfose | | 7966487.54 | | 11359648.17 | | 6117681.76 | | 29360790.01 | | 46550557.65 | | 36722383.8 | |  |  |  |
| Dihydroresveratrol | | 889312313.8 | | 804216280.2 | | 770190276.3 | | 3719603719 | | 3754826062 | | 3811521999 | |  |  |  |
| Orth0-Aminobenzoic Acid | | 86292743.05 | | 23819088.23 | | 16941326.75 | | 174014076.5 | | 184585700.2 | | 238618077 | |  |  |  |
| Syringic Acid | | 228719928.9 | | 194743441.5 | | 167519566.7 | | 934022369.9 | | 874265786.5 | | 984641166.9 | |  |  |  |
| Salicin | | 15835645.7 | | 16290606.54 | | 15122390.13 | | 65573167.16 | | 88783047.42 | | 71013426.49 | |  |  |  |
| Diferuloyl Glycerol | | 5468169.51 | | 3462086.89 | | 6237862.02 | | 23454641.01 | | 27477673.41 | | 22665637.13 | |  |  |  |
| Octopine | | 34390416.57 | | 33089499.24 | | 34633297.42 | | 180411989.9 | | 173748925.9 | | 167050490.7 | |  |  |  |
| Threonate | | 734282580.4 | | 567846541.6 | | 612659507.8 | | 3038634548 | | 3225982847 | | 3540362855 | |  |  |  |
| Mycosporine Glutaminol | | 121091471.8 | | 85468961.68 | | 94747888.11 | | 530385612.6 | | 492399466.2 | | 555444392.5 | |  |  |  |
| (2R)-6-Methylpiperidine-2-Carboxylic Acid | | 86590189.64 | | 87199269.59 | | 96453683.8 | | 577822933.5 | | 454536606 | | 421863597.4 | |  |  |  |
| **MetaboName** | | **SMM914-1** | | **SMM914-2** | | **SMM914-3** | | **CONTROL-1** | | **CONTROL-2** | | **CONTROL-3** | |  |  |  |
| Dehydrocostus Lactone | | 10235754.89 | | 8763834.06 | | 7978008.52 | | 54005590.66 | | 49217384.1 | | 47602069.41 | |  |  |  |
| Nicotinic Acid Mono Nucleotide | | 2841371.83 | | 3896541.06 | | 7315914.67 | | 21738852.27 | | 25365854.33 | | 36512488.27 | |  |  |  |
| N-Feruloyltyramine | | 9380578.12 | | 6042201.85 | | 0 | | 34196942.67 | | 31955644.16 | | 30069269.8 | |  |  |  |
| N-Fructosyl Isoleucine | | 146141527.2 | | 110649066.6 | | 111378995.5 | | 747410441.6 | | 689649933.5 | | 951520894.2 | |  |  |  |
| Sebuthylazine | | 38146297.12 | | 34830653.15 | | 30722794.4 | | 228457434.3 | | 254637470.4 | | 223497600.1 | |  |  |  |
| 3-Sulfino-L-Alanine | | 10512503.22 | | 5904889.14 | | 5779752.2 | | 53289049.87 | | 51361776.47 | | 51661926.62 | |  |  |  |
| Methohexital | | 278378823.2 | | 254572315.3 | | 298023240.2 | | 2081401538 | | 1972802269 | | 1855029980 | |  |  |  |
| Naproxen | | 10178965.16 | | 9955363.04 | | 9669053.39 | | 72102171.74 | | 71243868.02 | | 75109097.61 | |  |  |  |
| Cetraxate | | 11482442.27 | | 10289981.63 | | 10310312.28 | | 73796617.18 | | 89128674.45 | | 75064396.15 | |  |  |  |
| Ofloxacin | | 1278357456 | | 1127597002 | | 1031997135 | | 8597796578 | | 8720307142 | | 8528723120 | |  |  |  |
| N-Fructosyl Phenylalanine | | 233109627.5 | | 209282634.3 | | 221698804 | | 1990966183 | | 1665528625 | | 1827305382 | |  |  |  |
| Piperlotine A | | 14757195.75 | | 15394446.77 | | 17311121.94 | | 165617052.9 | | 120619982.9 | | 114767287.4 | |  |  |  |
| Acetaminophen Glucuronide | | 106853881.8 | | 95387777.68 | | 91120474.32 | | 967294079.5 | | 535048129.7 | | 1013760081 | |  |  |  |
| Maraviroc | | 323971.17 | | 15297.71 | | 482003.61 | | 1677902.15 | | 3836044.12 | | 1742600.26 | |  |  |  |
| N2,N2-Dimethylguanosine | | 29837628.25 | | 27488821.85 | | 23816321.04 | | 276255692.4 | | 252958276.7 | | 221194687.5 | |  |  |  |
| Xanthine | | 8656387.19 | | 8943092.21 | | 9994780.4 | | 85083404.7 | | 84915987.97 | | 86014949.5 | |  |  |  |
| Clemastine Fumarate | | 28306927.27 | | 28733692.7 | | 30006611.89 | | 274480646.2 | | 284900893.4 | | 261039302.2 | |  |  |  |
| Gibberellic Acid | | 3651127.51 | | 3092283.29 | | 2423038.75 | | 33291581.08 | | 26848234.07 | | 28038290.87 | |  |  |  |
| Quassin | | 4074862.55 | | 4241997.88 | | 2261900.09 | | 34986051.76 | | 35932873.23 | | 31182055.98 | |  |  |  |
| 17alpha-Estradiol | | 284162659.9 | | 288821924.3 | | 282521166.9 | | 2875804497 | | 2886445620 | | 2864283517 | |  |  |  |
| **MetaboName** | | **SMM914-1** | | **SMM914-2** | | **SMM914-3** | | **CONTROL-1** | | **CONTROL-2** | | **CONTROL-3** | |  |  |  |
| Gly-Tyr-Ala | | 16577362.77 | | 19373800.37 | | 14237698.42 | | 169933196.8 | | 156421671.6 | | 181716203.3 | |  |  |  |
| Indole-3-Acetyl-L-Leucine | | 114503113.2 | | 120578085.1 | | 118220450.9 | | 1183365009 | | 1206949057 | | 1203126270 | |  |  |  |
| Aminopyrine | | 14394261.77 | | 12471681.55 | | 13294381.33 | | 145710696.5 | | 138221392.5 | | 129404210.8 | |  |  |  |
| 9-Fluorenone | | 3404213120 | | 2915588092 | | 2749723763 | | 31052512993 | | 31827258997 | | 32385909710 | |  |  |  |
| Sempervirine | | 15906717.48 | | 25795905.55 | | 18932180.76 | | 239581460.3 | | 255968747.4 | | 157151718.1 | |  |  |  |
| L-Histidinol | | 10322505.73 | | 9829520.89 | | 10609809.95 | | 101479598.1 | | 107649511.9 | | 126614049.5 | |  |  |  |
| Hypothallin | | 11477586.81 | | 9302805.28 | | 8925272.36 | | 103731582 | | 100169295.4 | | 131192065.5 | |  |  |  |
| FT-OH | | 311439579 | | 255189936.7 | | 242495337.9 | | 3145317579 | | 3090034871 | | 3277094166 | |  |  |  |
| Calycanthine | | 12991548.51 | | 12157214.33 | | 11800589.03 | | 144592443.2 | | 154911434.7 | | 140870094.7 | |  |  |  |
| Picrolichenic Acid | | 36210789.15 | | 30116153.62 | | 28622619.18 | | 394181222.4 | | 403964876.3 | | 350493953.6 | |  |  |  |
| Carminic Acid | | 1214853.04 | | 1146353.21 | | 1656696.88 | | 18645192.3 | | 16203365.62 | | 16282721.57 | |  |  |  |
| 2-Methylglutaric Acid | | 242137563.4 | | 202050389.7 | | 203355357.9 | | 2814662300 | | 2780536426 | | 2773030286 | |  |  |  |
| Bicuculline (+) | | 862797.33 | | 812117.55 | | 1038378.89 | | 14027224.38 | | 14476555.37 | | 9231440.2 | |  |  |  |
| L-Prolyl-L-Isoleucine | | 379762373.7 | | 255902753 | | 257923187.7 | | 4196775152 | | 4239497225 | | 4141079997 | |  |  |  |
| Clobetasol Propionate | | 0 | | 2512181.28 | | 0 | | 13350177.67 | | 11588122.65 | | 13556338.42 | |  |  |  |
| Psicose | | 666666622.1 | | 596309788.6 | | 788869530.9 | | 10952770834 | | 10868038167 | | 10620555573 | |  |  |  |
| Duartin (-) | | 49321640.69 | | 50914438.41 | | 66920546.75 | | 937100468.3 | | 780568040.5 | | 996839655.2 | |  |  |  |
| Nevaltophin D | | 1587264.14 | | 1413300.1 | | 1506682.15 | | 32896211.67 | | 24928121.41 | | 23383565.86 | |  |  |  |
| Linderalactone | | 24237776.45 | | 24389277.55 | | 25079197.71 | | 439451626.7 | | 466190353 | | 459190283.6 | |  |  |  |
| Coumaroylquinic Acid | | 7519755.15 | | 6682264.61 | | 6790123.06 | | 130921674.4 | | 138303249.8 | | 131765875.6 | |  |  |  |
| **MetaboName** | | **SMM914-1** | | **SMM914-2** | | **SMM914-3** | | **CONTROL-1** | | **CONTROL-2** | | **CONTROL-3** | |  |  |  |
| 5,7-Dimethoxy-4'-Hydroxyflavanone | | 278559091.8 | | 3322354.54 | | 5371095.79 | | 1764015612 | | 1781229153 | | 1965453976 | |  |  |  |
| Tephrosin | | 2937824.59 | | 832331.16 | | 3138772.34 | | 42965625.94 | | 47879379.28 | | 43462680.44 | |  |  |  |
| Niacin | | 12632661.91 | | 15247691.21 | | 14048495 | | 277139290.6 | | 275373027 | | 264639171.1 | |  |  |  |
| Khivorin | | 2730696.81 | | 1402824.36 | | 1440271.67 | | 42775443.98 | | 37207512.35 | | 28700856.48 | |  |  |  |
| Serylleucine | | 119667955.4 | | 112296396.2 | | 113118128.2 | | 2300396180 | | 2306893021 | | 2214454122 | |  |  |  |
| 11A-Acetoxyprogesterone | | 344201.68 | | 1615597.36 | | 2398528.22 | | 34037180.17 | | 29248953.51 | | 26526087.49 | |  |  |  |
| Lappaconitine | | 707453.44 | | 916092.29 | | 517153.81 | | 16722282.76 | | 19724986.34 | | 15689362.06 | |  |  |  |
| 3-Hydroxycolensoic Acid | | 7299768.92 | | 4770091.08 | | 7038092.11 | | 161644930.6 | | 168018175 | | 141253992.7 | |  |  |  |
| Haematoxylin | | 84408157.55 | | 54338722.45 | | 59056546.88 | | 1501165774 | | 1574202139 | | 1858943072 | |  |  |  |
| Pregnenolone Acetate | | 1204584.1 | | 1186820.04 | | 1422941.86 | | 29832618.97 | | 36358890.59 | | 32969293.17 | |  |  |  |
| Denticulatin B | | 1506523.42 | | 1550098.29 | | 1450293.65 | | 39119818.73 | | 43709315.06 | | 39313982.38 | |  |  |  |
| Ala-Leu | | 38244644.65 | | 37971041.35 | | 36506307.4 | | 1067204953 | | 1043521766 | | 1032003520 | |  |  |  |
| Nafadotride | | 22133182.27 | | 20829807.77 | | 22681892.75 | | 668592708.9 | | 614386106.6 | | 568790686.5 | |  |  |  |
| D-(+)-Malic Acid | | 183174817.8 | | 155273482.4 | | 142355772.9 | | 4881061136 | | 4733988174 | | 4395820004 | |  |  |  |
| Crotonoside | | 984511 | | 0 | | 0 | | 6324785.17 | | 10765429.42 | | 11889521.94 | |  |  |  |
| D-Glucuronic Acid | | 174422017.8 | | 125083587.6 | | 125475963.9 | | 4306647900 | | 4230151609 | | 4240806212 | |  |  |  |
| Ramipril | | 547371.3 | | 535877.68 | | 836123.26 | | 20288283.83 | | 20769415.89 | | 17568022.94 | |  |  |  |
| Lysergol | | 2206515.08 | | 2420860.75 | | 2514654.39 | | 80293018.79 | | 73537720.93 | | 65955819.79 | |  |  |  |
| Volemitol | | 1759977.61 | | 2609913.43 | | 2309021.23 | | 67702373.69 | | 71327017.48 | | 69436264.56 | |  |  |  |
| Celastrol | | 1898942.79 | | 2949908.43 | | 2135164.05 | | 76997437.5 | | 73433700.97 | | 68707815.37 | |  |  |  |
| **MetaboName** | | **SMM914-1** | | **SMM914-2** | | **SMM914-3** | | **CONTROL-1** | | **CONTROL-2** | | **CONTROL-3** | |  |  |  |
| 7,8-Dihydroxyflavone | | 3958150.9 | | 2985724.64 | | 3031654.96 | | 118885241.6 | | 102099594.2 | | 117615886.9 | |  |  |  |
| D-Glucosamine | | 14728383.45 | | 15114047.45 | | 18420239.38 | | 566697954 | | 574526935.6 | | 546030440.1 | |  |  |  |
| Scrobiculin | | 8913826.3 | | 6061176.78 | | 6065631.72 | | 270855063.3 | | 258286258 | | 207139111.1 | |  |  |  |
| Leucylalanine | | 32215386.04 | | 30750518.36 | | 31352383.91 | | 1264087396 | | 1241571439 | | 1262530606 | |  |  |  |
| Threonylleucine | | 62094945.1 | | 61766785.69 | | 64476689.93 | | 2595523514 | | 2518440353 | | 2448487042 | |  |  |  |
| Mepacrine | | 955549.25 | | 506813.64 | | 789468.86 | | 34209238.85 | | 34074400.58 | | 33849294.32 | |  |  |  |
| Guanosine | | 19841375.21 | | 16440605.73 | | 14818665.72 | | 797713678 | | 795881046 | | 763881091.7 | |  |  |  |
| 1-Methylguanosine | | 982987.04 | | 1190017.51 | | 272974.6 | | 41331048.98 | | 38973704.41 | | 34813488.28 | |  |  |  |
| Trans-5-O-Caffeoylquinic Acid | | 19936656.79 | | 15511065.88 | | 24441708.09 | | 813850412.5 | | 702617003.8 | | 1405695493 | |  |  |  |
| Gly-Leu | | 7813767.46 | | 5711913.35 | | 4699525.68 | | 314365159.6 | | 318499283.1 | | 271970403.1 | |  |  |  |
| Gly-Ile | | 55721052.57 | | 55745636.47 | | 56257650.93 | | 2813794946 | | 2908851359 | | 2751124233 | |  |  |  |
| Succinic Acid | | 66426161.73 | | 60087052.42 | | 67024082.7 | | 3033650171 | | 3715190106 | | 3474322437 | |  |  |  |
| Ala-Val | | 39493252.75 | | 29150703.05 | | 29631512.82 | | 1751647471 | | 1737911590 | | 1727319306 | |  |  |  |
| Ellipticine | | 2518687.33 | | 1969772.15 | | 1802420.92 | | 112073222 | | 117658216.3 | | 117409772.3 | |  |  |  |
| Gly-Tyr | | 5653499.12 | | 4982456.92 | | 5624583.01 | | 257255316.9 | | 358863535.4 | | 361052334.9 | |  |  |  |
| Alanylphenylalanine | | 3593545.59 | | 3668738.29 | | 5424035.82 | | 253935243.4 | | 258280598.7 | | 256020752.3 | |  |  |  |
| 5-O-Caffeoylquinic Acid Methyl Ester | | 7144269.45 | | 3487242.38 | | 6264364.94 | | 308937266.6 | | 335732695.2 | | 411629857.8 | |  |  |  |
| Glipizide | | 364988.99 | | 1195719.63 | | 2044287.28 | | 80008428.16 | | 82654524.9 | | 77283713.35 | |  |  |  |
| Isoleucylglutamine | | 28535703.95 | | 31821655.36 | | 32773123.71 | | 2166305724 | | 2230677695 | | 2133084328 | |  |  |  |
| Inosine | | 16103174.56 | | 15055376.45 | | 16739427.21 | | 1133403788 | | 1124269134 | | 1109557531 | |  |  |  |
| **MetaboName** | | **SMM914-1** | | **SMM914-2** | | **SMM914-3** | | **CONTROL-1** | | **CONTROL-2** | | **CONTROL-3** | |  |  |  |
| Lobaric Acid | | 663069.29 | | 570755.23 | | 165650.1 | | 30963247.57 | | 30364007.58 | | 39054860.46 | |  |  |  |
| Trehalose | | 5319909.84 | | 3892577.07 | | 5191123.99 | | 346068524.6 | | 348899026.5 | | 357539346.8 | |  |  |  |
| Cirsimaritin | | 1960499.1 | | 1149456.89 | | 970850.19 | | 90248699.69 | | 102180791.8 | | 111780035.5 | |  |  |  |
| Acetylportentol | | 7479900.86 | | 4953658.97 | | 5071629.55 | | 488767330.2 | | 477818989.4 | | 466618640 | |  |  |  |
| Ranolazine | | 552886.13 | | 0 | | 421526.67 | | 36108711.92 | | 34042960.04 | | 21455311.65 | |  |  |  |
| Isoformosanine | | 2788238.07 | | 2478590.17 | | 3115186.79 | | 284258155 | | 281113155.4 | | 279360816 | |  |  |  |
| Isomajdine | | 1105162.71 | | 428905.9 | | 330830.37 | | 63046631.05 | | 65666452.8 | | 60060314.09 | |  |  |  |
| Syringaresinol | | 194583.26 | | 181590.81 | | 143854.06 | | 15711814.9 | | 20177938.72 | | 17371254.63 | |  |  |  |
| Sinensetin | | 91142.16 | | 125631.91 | | 128409.56 | | 13946608.97 | | 12466962.71 | | 13040917.49 | |  |  |  |
| Scalarin | | 171011.98 | | 62696.15 | | 334233.42 | | 22106200.55 | | 23173423.09 | | 21563340.97 | |  |  |  |
| 5,7-Dihydroxyflavanone | | 54397243.1 | | 1757505.61 | | 3827598.45 | | 2834149935 | | 1708947372 | | 2970906029 | |  |  |  |
| Melibiose | | 1522427.78 | | 2088760.53 | | 2145355.39 | | 312402090.7 | | 245630477.1 | | 239161256 | |  |  |  |
| Gly-Val | | 11909162.22 | | 9150282.02 | | 8218348.9 | | 1646028017 | | 1659731826 | | 1232337775 | |  |  |  |
| 1-Methyladenosine | | 4732370.22 | | 2925433.84 | | 4854847.79 | | 756411402.7 | | 689165974.8 | | 661933289 | |  |  |  |
| Tyr-Tyr-Tyr | | 1508987.06 | | 485042.88 | | 1112632.68 | | 188177063 | | 168150594.6 | | 188918431.4 | |  |  |  |
| Cycloveratrylene | | 56248.17 | | 104263.63 | | 0 | | 8863640.04 | | 9894164.72 | | 9577295.84 | |  |  |  |
| Isomaltulose | | 417749.87 | | 1561475.19 | | 2010909.44 | | 240077035 | | 258746711 | | 244580587.3 | |  |  |  |
| Diacetylsongorine | | 246711.33 | | 0 | | 61864.5 | | 20153451.5 | | 18597664.5 | | 19084739.04 | |  |  |  |
| Uridine | | 17456752 | | 10325396.83 | | 12595911.55 | | 3567149841 | | 3518678859 | | 3523291779 | |  |  |  |
| Prolylyphenylalanine | | 239548.74 | | 1306804.74 | | 106665.27 | | 154891162.8 | | 151415412.4 | | 147390686.1 | |  |  |  |
| **MetaboName** | | **SMM914-1** | | **SMM914-2** | | **SMM914-3** | | **CONTROL-1** | | **CONTROL-2** | | **CONTROL-3** | |  |  |  |
| Atranorin | | 936143.36 | | 595509.46 | | 295900.12 | | 148757364.3 | | 211768733.2 | | 233920600.8 | |  |  |  |
| Isoleucylisoleucine | | 4826460.46 | | 4645952.38 | | 4327554.78 | | 1682223424 | | 1718913166 | | 1659394481 | |  |  |  |
| Colchicine | | 501441.38 | | 1199307.81 | | 658310.6 | | 304221160.4 | | 292488663.9 | | 270112184.6 | |  |  |  |
| Methanesulfonate | | 7795875.98 | | 5261524.67 | | 5284931.88 | | 2284404393 | | 2306493026 | | 2465934836 | |  |  |  |
| Leucylvaline | | 6423996.73 | | 10374353.8 | | 8658273.58 | | 4443056743 | | 4353554604 | | 4166952766 | |  |  |  |
| Baohuoside I | | 86734.51 | | 310148.35 | | 267897.1 | | 134283725.8 | | 125883410.5 | | 124939096 | |  |  |  |
| Oregonin | | 123601.09 | | 0 | | 0 | | 28409042.04 | | 25550580.65 | | 23377676 | |  |  |  |
| (-)-Podophyllotoxin | | 0 | | 351902.73 | | 366808.29 | | 249233510.7 | | 228228652.3 | | 112586101.6 | |  |  |  |
| Alpha-Mangostin | | 206849.45 | | 0 | | 0 | | 71446284.13 | | 72062675.6 | | 64439791.19 | |  |  |  |
| Carbetamide | | 499743.42 | | 0 | | 0 | | 199659979.8 | | 205368101.6 | | 180475983.4 | |  |  |  |
| Hydroxygardnutine | | 233782.44 | | 262587.72 | | 812120.38 | | 543995824.6 | | 583501325.7 | | 546652254.3 | |  |  |  |
| Adenosine | | 13716806.56 | | 14035609.9 | | 12337887.63 | | 28478142465 | | 30830290600 | | 30474902705 | |  |  |  |
| N-Acetylaspartylglutamate | | 348889.9 | | 496502.04 | | 255624.32 | | 774915856.7 | | 749509832.1 | | 989904114.6 | |  |  |  |
| Flunisolide | | 0 | | 0 | | 0 | | 8820865.88 | | 9059411.2 | | 10805017.15 | |  |  |  |
| Taurolithocholic Acid | | 0 | | 0 | | 0 | | 12230046.91 | | 12863877.05 | | 12235698.91 | |  |  |  |
| Digoxigenin | | 0 | | 0 | | 0 | | 17344030.55 | | 18258738.33 | | 17342043.61 | |  |  |  |
| Kuwanon C | | 0 | | 0 | | 0 | | 51696778.17 | | 54135455.88 | | 49335501.35 | |  |  |  |
| Benidipine | | 0 | | 0 | | 0 | | 55722835.44 | | 51770533.89 | | 58273619.27 | |  |  |  |
| Epigallocatechin-3-Monogallate | | 0 | | 0 | | 0 | | 62571199.02 | | 54825275.56 | | 66905137.11 | |  |  |  |
